# Comparison between moderate-load and high-load exercises in the rehabilitation of runners with Achilles tendinopathy: Protocol for a blind randomized controlled trial

**DOI:** 10.1371/journal.pone.0342934

**Published:** 2026-03-02

**Authors:** Magno Jackson Moreno, Anne-Sofie Agergaard, Mateus Antônio Albuquerque Costa, Rene Brüggebusch Svensson, S. Peter Magnusson, Rodrigo Scattone Silva

**Affiliations:** 1 Postgraduate Program in Physical Therapy, Federal University of Rio Grande do Norte, Natal, Brazil; 2 Brazilian Tendinopathy and Sports Injuries Research Group (BRATSI), Health Sciences College of Trairi, Federal University of Rio Grande do Norte, Santa Cruz, Brazil; 3 Institute of Sports Medicine Copenhagen, Department of Orthopedic Surgery, Copenhagen University Hospital - Bispebjerg and Frederiksberg, Copenhagen, Denmark; IRCCS Istituto Ortopedico Rizzoli, ITALY

## Abstract

**Background:**

Achilles tendinopathy is one of the most frequent conditions affecting runners. Exercise-based interventions are the main treatment options for Achilles tendinopathy; however, the ideal exercise dosage remains unknown.

**Objective:**

The objective of this study is to investigate the effects of a moderate-load exercise intervention compared to a high-load exercise intervention in runners with Achilles tendinopathy in terms of pain, symptom severity, quality of life, muscle strength, and function.

**Methods:**

Sixty amateur runners will be randomly allocated to one of two groups: high-load exercise group (HLG) and moderate-load exercise group (MLG). The HLG will start the treatment with calf exercises with 55% of 1 repetition maximum (1RM) and progressively increase the exercise load in 12 weeks to 90% 1RM. The MLG will perform exercises with 55% 1RM during the 12 weeks of intervention. The RM load will be reassessed every 2 weeks, and the total training volume (total repetitions and tendon time under tension per week) will be the same in both groups. Both groups will also perform strengthening exercises for the quadriceps and gluteal muscles. The primary outcome will be symptom severity (Victorian Institute of Sport Assessment−Achilles), which will be evaluated at baseline, at week 6, at week 12 (end of intervention), and 6 months after the intervention. Pain will be evaluated using the visual analogue scale during the same time points. Secondary outcomes will include peak isometric strength of ankle, knee, and hip muscles, function of plantar flexor muscles, quality of life, and perception of improvement. Results will be analyzed under the intention-to-treat principle using generalized estimating equations (GEE) and Bonferroni-adjusted post hoc tests.

**Discussion:**

This will be the first randomized controlled trial comparing high-load exercises versus moderate-load exercises in individuals with Achilles tendinopathy applying equal training volume.

**Trial registration number:**

RBR-4vwy5xj

## Introduction

Achilles tendinopathy is a common musculoskeletal condition affecting both active and sedentary individuals [1]. The incidence of Achilles tendinopathy is 2–3 per 1,000 individuals in the general population and is even more common in physically active individuals [2]. In a cohort study with 3,379 participants carried out among recreational runners, the incidence of Achilles tendinopathy was 4.2%, and of these, 64% had mid-portion Achilles tendinopathy [3]. In elite distance runners, the prevalence of Achilles tendinopathy in their lifetime can be as high as 52% [4]. Males have also been shown to be more affected by Achilles tendinopathy than females [3]. In addition to being highly prevalent, Achilles tendinopathy can have a substantial impact on an individual’s long-term health and quality of life [5].

Currently, interventions involving exercises with gradual load progression for clinical improvement and positive tendon adaptation are considered the first-line treatment for Achilles tendinopathy [1,6,7]. The classic Alfredson eccentric training protocol remains one of the most popular interventions among clinicians [8]. However, patient recovery rates with traditional interventions, such as the Alfredson protocol, seem to be far from ideal [9–12]. A few studies report that between 20‒40% of patients experience unsatisfactory results after 10 years of treatment [9,10] and that only 39% of patients treated with the Alfredson protocol are asymptomatic after 5 years [11]. A recent systematic review also concluded that there is low quality of evidence that the Alfredson protocol yields superior results to wait-and-see and passive interventions [12]. Therefore, there is a need for research on the effects of other treatment approaches that investigate the effects of other rehabilitation interventions for Achilles tendinopathy with long-term evaluations.

A few studies have evaluated the variation in progressive loading parameters [13,14], such as the number of repetitions, sets, frequency, and load, to find an ideal exercise dose and load magnitude during interventions in patients with tendinopathy [15]. Recently, Arampatzis et al. [16] suggested that when comparing exercise programs with the same volume, but with different loads, only protocols with greater load produce adaptations in the tendons, such as increased stiffness and increased cross-sectional area in people with healthy Achilles tendons. It is believed that this occurs because high-magnitude contractions cause deformation (strain) of the tendon within the range considered ideal (between 4.5 and 6%) for positive mechanical and morphological adaptations [16]. High-load exercises (90% of 1 repetition maximum (1RM)), in addition to improving strength and generating hypertrophy, can cause an effective mechanical stimulus in the Achilles tendon that stimulates beneficial adaptations in the tissue, causing improvement in the mechanical properties of healthy Achilles tendons [16,17]. The same research group has observed, in a non-randomized controlled study, that an intervention involving isometric exercises performed at 90% 1RM resulted in more structural adaptations in the Achilles tendon of patients with Achilles tendinopathy when compared with the Alfredson protocol and with passive treatments [18]. However, recent evidence suggests that tendon structural adaptations also occur in tendinopathic patellar tendons in response to moderate-load progressive exercises (55% 1RM) [13,19,20].

To our knowledge, however, the effects of exercises with different load magnitudes on individuals with Achilles tendinopathy remain unknown. The primary objectives of the study will be to verify the short-term (6 and 12 weeks) and long-term (6 months) effects of moderate-load calf exercises compared to high-load calf exercises in amateur runners with Achilles tendinopathy in terms of pain and severity of symptoms. The secondary objectives will be to verify the effect of the interventions on strength, muscle function, and quality of life in runners with Achilles tendinopathy.

## Materials and methods

### Study design and trial status

This is the protocol for a randomized, blind, parallel group (1:1) controlled trial comparing two exercise programs for runners with Achilles tendinopathy. The study will be carried out at the Department of Physiotherapy at the Federal University of Rio Grande do Norte (UFRN) and will follow the guidelines of the Standard Protocol Items: Recommendations for Interventional Trials (SPIRIT) (S1 File) [21]. Participants will be randomized into one of two groups: high-load group (HLG) and moderate-load group (MLG). This clinical trial will be reported following the Consolidated Standards of Reporting Trials (CONSORT) guidelines [22] and the protocol was registered on the Brazilian Registry of Clinical Trials (ReBEC). The recruitment of participants began on June 4^th^, 2024. It is expected that the last participants will be recruited by October 31^st^, 2025. The final data collections will take place in July 2026, and results should be completed by September 2026.

The project (S2 File) was approved by the Research Ethics Committee of Federal University of Rio Grande do Norte, Health Sciences College of Trairi – UFRN/FACISA (process 6729527; CAAE 78022924.3.0000.5568), based on protocol version 2.0, dated March 12, 2024. Evaluators will obtain participants’ signatures on the Informed Consent Form and on the authorization form for voice and image recording. The individuals pictured in “S2_File_ Protocol_approved_by_ethics committee” (Figs 3, 8, 10, 11, 14), “S3_File_Assessment”, and “S4_File_Treatment_Protocol” have provided written informed consent (as outlined in PLOS consent form) to publish their image alongside the manuscript. The results will be disseminated through publications in scientific journals, social media, and presentations at scientific events.

### Eligibility criteria

Recreational male runners aged 18–60 years with midportion Achilles tendinopathy will be included. The clinical diagnosis of midportion Achilles tendinopathy will be performed by registered physical therapists with more than 4 years of practice experience in musculoskeletal physiotherapy. The diagnosis will follow the following criteria: localized pain in the midportion of the Achilles tendon (two to six centimeters above the tendon insertion into the calcaneus) with a minimum duration of three months and a minimum intensity of 3 on the Visual Analogue Scale (VAS), pain during loading activities such as running, and pain on palpation [7,23–25].

Individuals will be excluded if they present (1) exclusively insertional Achilles tendinopathy, (2) a washout period of less than 4 weeks from other treatments, (3) use of corticosteroid injections in the Achilles tendon region, or use of fluoroquinolone antibiotics in the last 12 months [26], (4) other injuries of the affected lower limb in the last 6 months, (5) musculoskeletal surgery on the spine or lower limbs over the previous 12 months, (6) history of Achilles tendon rupture, and (7) systemic diseases that may interfere with tendon health (i.e., rheumatoid arthritis or diabetes) [23].

Participants’ recruitment will be carried out through an active search of registrations of running groups active in the city of Natal, Rio Grande do Norte (Brazil). Online ads in social media (Instagram, WhatsApp, Telegram, Facebook) and printed banners at traditional race locations in the city will be used as advertisement strategies. Runners experiencing Achilles tendon pain will be invited for clinical assessment of possible eligibility.

After eligibility is established, for sample characterization purposes, each participant’s level of physical activity will be assessed using the short version of the International Physical Activity Questionnaire-Short Form (IPAQ-SF) [27], and for the assessment of kinesiophobia, the Brazilian Portuguese version of the Tampa Scale of Kinesiophobia (TSK) will be used [28] at the baseline evaluation.

### Sample size calculation

To determine the study sample size, a calculation was made considering a confidence level (α) of 95%, a power (β) of 80%, and a difference between groups in the Victorian Institute of Sport Assessment-Achilles (VISA-A) at 12 weeks of 14 points [24]. The standard deviation used in the calculations was obtained from a similar clinical trial that used this same variable as the primary outcome [23]. Using this information, calculations for linear mixed models indicated a sample size of 54 individuals (27 per group). Considering a loss of 10%, we reached a total number of 60 individuals, with 30 in each group.

### Randomization and strategies for minimizing bias

The randomization of participants will be generated using a sample randomization system (block of four) creating a list of random numbers (http://www.randomization.com). An independent researcher who will not be involved with assessments, interventions, or data analysis will be responsible for generating the list. From this list, a set of opaque envelopes will be created and sealed, ensuring allocation concealment.

The evaluators will remain blind to the groups and will perform all study assessments. To ensure blinding, they will not participate in the interventions, and participants will be instructed not to discuss details of their intervention with the other participants or the evaluator. Participants will be randomized at the beginning of the first in-person intervention session. The interventions will be carried out at the Department of Physiotherapy of the Federal University of Rio Grande do Norte (UFRN) and/or local gyms and will be supervised by experienced physiotherapists.

### Outcomes

The main primary outcome of the study will be symptom severity, which will be measured by the VISA-A questionnaire. Pain will be assessed by the VAS where 0 represents no pain and 10 represents the worst pain imaginable [29]. Pain will be assessed during the heel rise test and during the single-leg drop vertical jump test [30,31] (described below). Participants will also be asked about their worst pain in the previous week [23]. Symptom severity will be evaluated with the Brazilian Portuguese version of the VISA-A questionnaire [32]. Improvements greater than 3 points in the VAS [33] and greater than 14 points in the VISA-A are considered clinically meaningful [24,33]. Symptom severity and pain will be assessed before the interventions (baseline), at week 6, at week 12 (end of the interventions), and 6 months after the end of the interventions (follow-up).

Secondary outcomes will include peak isometric strength of hip and knee extensors and ankle plantar flexors, function of the plantar flexor muscles (heel rise test and single-leg drop vertical jump test), quality of life, and perception of improvement or worsening of the participants’ condition.

A handheld dynamometer (Lafayette Instruments, IN, USA) will be used to measure the peak isometric strength of the hip extensors, knee extensors, and ankle plantar flexors [34]. Inelastic straps will be used to stabilize the participants and to fixate the dynamometer, to eliminate the effect of the evaluator’s strength in the tests [34,35]. A detailed description of the assessments is presented in the Supplementary Material (S3 File). For each test, one practice and 3 valid trials will be performed. Each trial will consist of 5s maximal contraction, with 15s of rest between trials. The peak-force values produced during each of the 3 trials will be recorded. Peak-force values (kg) will be converted to Newtons (kg x 9.81) to achieve a unit of force. Newtons will then be converted to torque values [force (N) x action length of the segment (m)]. The action length of the thigh, shank, and foot will be measured as previously described (S3 File) [35], for the calculation of the hip extensor torque, knee extensor torque, and ankle plantar flexor torque, respectively. Finally, the torque data will be normalized against each participant’s body mass. The average normalized peak torque values obtained during the 3 trials will be used for analysis [34,35].

To evaluate the function of the plantar flexor muscles, two tests will be carried out: the heel rise test and the single-leg drop vertical jump test [31,36]. A detailed description of the tests is presented in the S3 File. The Calf Raise application will be used to measure the maximal number of repetitions and the total work (Joules) during the heel rise test [37], which will be used for analysis. Three repetitions of the single-leg drop vertical jump test will be performed, and the average height will be used for analysis (S3 File). Immediately after the performance of the heel rise test and the single-leg drop vertical jump test, participants will be asked to rate the Achilles tendon pain that they felt during the test in the 0–10 VAS [36].

**Fig 1:**
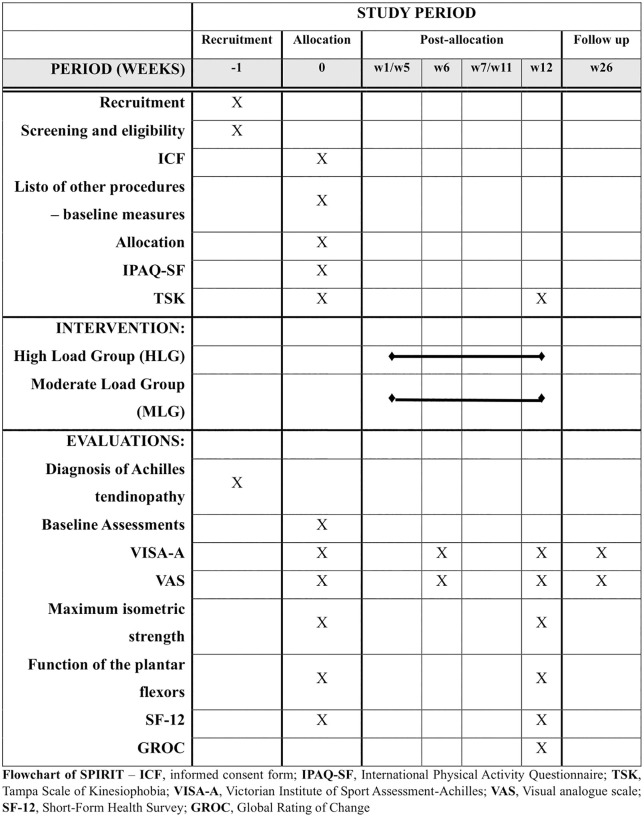
Flowchart of SPIRIT.

The Global Rating of Change (GROC) will be used to assess the participant’s perception of improvement or worsening after the interventions [38]. Changes of 4 points or more have previously been considered clinically important in patients with Achilles tendon pain [38,39]. Finally, to assess the impact of Achilles tendinopathy on participants’ quality of life, the Short-Form Health Survey (SF-12) will be used [40,41]. Secondary outcomes will be assessed before the interventions (baseline) and immediately after 12 weeks of interventions, except for the GROC, which will be evaluated only at the 12-week timepoint. The SPIRIT flowchart (Fig 1) illustrates the sequence of activities that will be performed during the study.

### Interventions

Participants of both groups will be submitted to 12 weeks of exercise-based progressive loading interventions in phases, following the recommendations of recent studies [7,42,43]. Specifically, the exercise protocol of both groups will be divided into three phases: 1) Slow-velocity progressive load exercises (3 seconds for the concentric phase and 3 seconds for the eccentric phase) with double-leg support (weeks 1–4), 2) Slow-velocity progressive load exercises in single-leg support (weeks 5–8), and 3) Plyometric exercises and running-specific strengthening exercises (weeks 9–12). The interventions of both groups are described in detail in the Supplementary Material (S4 File) following the recommendations of the Template for Intervention Description and Replication (TIDieR) [44].

Participants will be divided into two groups that will have equivalent total exercise volumes for the triceps surae: HLG which will begin triceps surae exercises with 55% 1RM in the first and second weeks, progressing to 65% 1RM in the third and fourth weeks, 75% 1RM in the fifth and sixth weeks, 85% 1RM in the seventh and eighth weeks and, 90% 1RM from the ninth week until the end of the 12 weeks of treatment; MLG which will perform triceps surae exercises with 55% 1RM during all 12 weeks of intervention (Table 1).

**Table 1:** Triceps surae exercise protocols for both groups.

	Weeks 1–2	Weeks 3–4	Week 5	Week 6	Week 7	Week 8	Weeks 9–12	Total Reps
HLG % 1RM	55	65	75	75	85	85	90	1,054
HLG Reps	15	15	10	8	8	6	4	
HLG Sets	3	3	3	4	4	4	5	
MLG % 1RM	55	55	55	55	55	55	55	1,053
MLG Reps	17	15	12	10	8	7	6	
MLG Sets	3	3	3	3	3	3	3	

HLG, high-load group; MLG, moderate-load group; RM, repetition maximum; Reps, repetitions.

In addition to specific exercises for the triceps surae, both groups will receive strengthening exercises for the muscles of the kinetic chain (i.e., quadriceps and gluteus maximus), which are synergists of the triceps surae during running and have been shown to have strength deficits in individuals with Achilles tendinopathy [45,46]. These exercises will be performed following the recommendations for strength training in resistance-trained individuals from the American College of Sports Medicine (ACSM) [47]. Specifically, these exercises will be performed in the following manner: in the first phase (1^st^ − 4^th^ weeks), participants will perform the exercises in 3 sets of 12 repetitions with 60% 1RM, in the second phase (5^th^ − 8^th^ weeks) they will perform the exercises in 3 sets of 10 repetitions with 70% 1RM, and in the third phase (9^th^ − 12^th^ weeks) they will perform the exercises in 3 sets of 8 repetitions with 80% 1RM [47] (S4 File).

Every two weeks, during the supervised session, a 10RM test will be performed to estimate 1RM and adjust training loads of all exercises accordingly [48,49]. In exercises where resistance will be provided by an elastic band, two levels of elastic resistance lower than 1RM will be established as the initial load, and the load will progress by increasing 1 level of elastic resistance [50].

Participants of both groups will be instructed to refrain from performing intense physical activities that may overload the Achilles tendons (i.e., daily running) during the duration of the study. If they are unwilling to do so, they will be asked to refrain from such activities for the first 3 weeks of treatment [23]. After this period, they will be allowed to resume their running routine using the pain monitoring model as a guide, i.e., avoid running distances/intensities that cause their pain to increase to more than 4/10 during or in the day after the run [51].

Interventions will take place 3 days a week on alternating days for twelve weeks. At least one of the weekly treatment sessions will be carried out with supervision [13]. Supervision will be carried out in person by experienced physical therapists trained in the clinical trial protocols. For the other two weekly non-supervised sessions, participants will receive illustrated material with guidance on the exercise parameters they should perform (S4 File). Adherence to exercises during non-supervised sessions will be monitored through a training diary, where participants will record the exercises performed and the training load.

In Phase 3 (9^th^ − 12^th^ weeks), participants will perform plyometric exercises during the supervised training session, given that individuals with Achilles tendinopathy have been shown to exhibit deficits in muscular power [52]. Plyometric training is considered one of the most effective modalities to counteract this impairment [53]. In the remaining two weekly sessions, participants will continue with the Phase 2 exercise protocol, with progressive adjustments to load, sets, and repetitions, as outlined in Table 1.

### Monitoring for adverse events

All participants will use a training diary to record the non-supervised sessions. Training records will include information on training intensity (repetitions, sets, and loads), sessions, soreness during training (VAS), and deviations from planned intervention protocols [13].

If any adverse events occur during or after the intervention, the participant will be advised to contact one of the physical therapists responsible for the intervention. The adverse events will be registered in detail and described at the conclusion of the study. Participants will be instructed not to perform any other type of treatment and not to use analgesic medications during the study period. If any pain medication or analgesic modality is used during the intervention period, the participants will be instructed to take note of the quantity and dosage of each medication and the parameters of the analgesic resource, reporting this information in the post-intervention assessments [54].

### Statistical analysis

The data will be analyzed by a researcher who will remain blind to group allocation. The Statistical Package for the Social Science software (version 17.0; SPSS Inc, Chicago, IL) will be used to analyze the results. To assess the normality of data, the Shapiro-Wilk test will be used. Outcome measures will be analyzed using generalized estimating equations (GEE) to identify main effects of group and time, and group-by-time interactions. Bonferroni-adjusted post hoc tests will be used when appropriate. The results will be presented as mean differences and 95% confidence intervals (CI). All data will be analyzed using the intention-to-treat principle, and in the event of missing data, a regression-based imputation technique will be used [55].

## Supporting information

S1 File SPIRIT checklist. (DOCX)

S2 File Protocol approved by the ethics committee. (DOCX)

S3 File Detailed Assessment Description. (DOCX)

S4 File Detailed Treatment Protocol. (DOCX)
