# Supplementary material for: Comparison between moderate-load and high-load exercises in the rehabilitation of runners with Achilles tendinopathy: Protocol for a blind randomized controlled trial
Source: PLoS One. 2026 Mar 2;21(3):e0342934. doi: 10.1371/journal.pone.0342934 (PMC12952620; doi:10.1371/journal.pone.0342934)
Supplement: S2 File — (DOCX) [file pone.0342934.s002.docx]

UNIVERSIDADE FEDERAL DO RIO GRANDE DO NORTE

PROGRAMA DE PÓS-GRADUAÇÃO EM FISIOTERAPIA

**Comparação entre exercícios resistidos de carga moderada e exercícios resistidos de alta carga na reabilitação de corredores com tendinopatia de Aquiles: Ensaio controlado randomizado e cego**

Autor: Magno J. Moreno

Natal/RN 2023

Magno J Moreno

Comparação entre exercícios resistidos de carga moderada e exercícios resistidos de alta carga na reabilitação de corredores com tendinopatia de Aquiles: Ensaio controlado randomizado e cego

Proposta de dissertação como requisito para obtenção do título de doutor em Fisioterapia, na subárea de avaliação e intervenção no sistema musculoesquelético.

**Orientador:** Prof. Dr. Rodrigo Scattone da Silva

Natal/RN 2023

LISTA DE FIGURAS

[**Figura 1**: Posição de teste para avaliação do torque de extensão do quadril (a),](#_bookmark10) [torque extensor do joelho (b) e torque flexor plantar do tornozelo (c) (Scattone Silva](#_bookmark10) [et al., 2016). .....1](#_bookmark10)6

[**Figura 2:** Teste de elevação na ponta do pé (SILBERNAGEL et al., 2011).....................1](#_bookmark11)7

[**Figura 3:** Teste de salto unipodal vertical máximo. medida da altura do chão para a](#_bookmark12) [ponta dos dedos das mãos do participante com o membro superior elevado (a);](#_bookmark12) [início do salto de uma caixa de 20 cm (b); preparação para o salto vertical com](#_bookmark12) [apenas um dos pés (c), ....1](#_bookmark12)8

[**Figura 4:** Fluxograma do SPIRIT – VISA-A, Victorian Institute of Sport Assessment-](#_bookmark13) [Achilles; EVA, Escala visual analógica; TSK, Tampa Scale of Kinesiophobia; IPAQ-](#_bookmark13) [SF, International Physical Activity Questionnaire; SF-36, Short-Form Health Survey;](#_bookmark13) [GROC, Global Rating of Change ….](#_bookmark13)19

[**Figura 5:** Posição inicial do exercício de elevação do calcanhar na ponta dos pés](#_bookmark18) [bipodal sentado saindo da dorsiflexão máxima do tornozelo (a); Posição final do](#_bookmark18) [exercício de elevação na ponta dos pés bipodal sentado terminando em flexão](#_bookmark18) [plantar máxima do tornozelo (b). .....2](#_bookmark18)2

[**Figura 6:** Elevação do calcanhar na pontas dos pés bipodal: posição inicial em uma](#_bookmark19) [dorsiflexão máxima (a); Posição final em uma flexão plantar máxima (b). .....2](#_bookmark19)2

[**Figura 7:** Posição inicial o exercício de extensão do joelho na cadeira extensora (a);](#_bookmark20) [Posição final do exercício de extensão do joelho na cadeira extensora (b). .....2](#_bookmark20)3

[**Figura 8:** Posição inicial e final para o exercício de ostra com 60° de flexão do](#_bookmark21) [quadril (a); Posição intermediária para o exercício de ostra com 60° de flexão do](#_bookmark21) [quadril (b). .....2](#_bookmark21)3

[**Figura 9:** Elevação do calcanhar sentado unipodal: posição inicial em dorsiflexão](#_bookmark23) [máxima do tornozelo (a) e posição final em flexão plantar máxima do tornozelo (b).](#_bookmark23) [Elevação do calcanhar na ponta do pé unipodal: posição inicial em dorsiflexão](#_bookmark23) [máxima do tornozelo (c) e posição final em flexão plantar máxima do tornozelo (d).](#_bookmark23)....[.......................................................................................................................................................2](#_bookmark23)4

[**Figura 10:** Posição inicial para o exercício de extensão do joelho na cadeira](#_bookmark24) [extensora (a). Posição final para o exercício de extensão do joelho na cadeira](#_bookmark24) [extensora (b)........2](#_bookmark24)4

[**Figura 11:** Exercício de *deadlift* unipodal: Posição inicial (a), posição final (b)..... .........2](#_bookmark25)5

[**Figura 12:** Saltos bipodais rápidos. ..2](#_bookmark27)6

[**Figura 13:** Saltos unipodais alternados rápidos. ..2](#_bookmark28)6

[**Figura 14:** Exercício de corrida na parede: Posição inicial de pé (a); Posição para](#_bookmark31) [iniciar a alternância entre os pés de apoio](#_bookmark31) ..28

LISTA DE TABELAS

[**TABELA** 1: Protocolos de exercícios de tríceps sural de ambos os grupos ...2](#_bookmark16)0

LISTA DE ABREVIATURAS E SIGLAS

ACSM American College of Sports Medicine

CONSORT *Consolidated Standards of Reporting Trials*

EVA Escala Visual Analógica

GAI Grupo de Alta Intensidade

GMI Grupo de Moderada Intensidade

GROC Global Rating of Change

IC Intervalo de Confiança

IPAQ-SF International Physical Activity Questionnaire-Short Form

TSK Tampa Scale of Kinesiophobia

MCID Diferença Clinicamente Importante

ReBEC Registro Brasileiro de Ensaios Clínicos

RM Repetição Máxima

SEM Erro padrão da média

*SF-12 Short-Form Health Survey*

SPIRIT *Standard Protocol Items: Recommendations for Interventional Trials*

SPSS Statistical Package for the Social Science

UFRN Universidade Federal do Rio Grande do Norte

VISA-A Victorian Institute of Sport Assessment-Achilles

**SUMÁRIO**

[RESUMO](#_bookmark0) 8

[ABSTRACT](#_bookmark1) 9

[INTRODUÇÃO 1](#_bookmark2)0

METODOLOGIA..........................................................................................................12

[Desenho do estudo 1](#_bookmark3)2

[Critérios de elegibilidade 1](#_bookmark4)2

[Cálculo amostral 1](#_bookmark5)3

[Randomização e estratégias para minimização de viés 1](#_bookmark6)3

[Avaliações 1](#_bookmark7)4

[Intervenções](#_bookmark14) 19

[**FASE 1 (do início da intervenção até o final da quarta semana)** 2](#_bookmark17)1

[**FASE 2 (da 5ª semana até o final da 8ª semana)** 2](#_bookmark22)4

[**FASE 3 (da 9ª semana até o final da 12ª)** 2](#_bookmark26)5

[REFERÊNCIAS 3](#_bookmark37)1

[ANEXOS](#_bookmark38) 38

**RESUMO**

**Introdução:** A tendinopatia de Aquiles apresenta alta incidência na população em geral e uma incidência ainda maior na população ativa e em atletas. Entre corredores, é uma das condições de saúde mais frequentes, causando grande impacto na qualidade de vida desses indivíduos. Apesar de sua importância, as abordagens atuais de tratamento apresentam resultados insatisfatórios em acompanhamentos de 5 a 10 anos. **Objetivo:** O objetivo primário do estudo será verificar os efeitos a curto (6 e 12 semanas) e a longo prazo (6 meses) de uma intervenção de exercícios resistidos de carga moderada [55% de 1 Repetição Máxima (55%1RM)] em comparação a exercícios resistidos de alta carga (90% de 1RM) em corredores com tendinopatia de Aquiles em termos de dor e severidade de sintomas. **Método:** Sessenta corredores amadores serão alocados aleatoriamente em dois grupos: grupo de exercícios de alta intensidade (GAI) e grupo de exercícios de moderada intensidade (GMI). O GAI iniciará o tratamento com exercícios de tríceps sural com 55%1RM na primeira e segunda semana, 65%1RM na terceira e quarta semana, 75%1RM na quinta e sexta semana, 85%1RM na sétima e oitava semana e, a partir da nona semana até o final das 12 semanas de tratamento, realizará os exercícios com 90%1RM. O GMI realizará exercícios com 55%1RM durante as 12 semanas de intervenção. A carga de RM será reavaliada a cada 2 semanas e o volume de treinamento total será idêntico em ambos os grupos. Ambos os grupos farão exercícios de fortalecimento para quadríceps e glúteos e continuarão realizando as atividades físicas/esportivas com a estratégia do modelo de monitoramento da dor. Serão avaliadas a dor (EVA) e severidade dos sintomas (VISA-A), força isométrica máxima de músculos do tornozelo, joelho e quadril (dinamômetro manual), função dos flexores plantares (testes funcionais), qualidade de vida (SF-12) e a percepção de melhora dos participantes (GROC). Desfechos primários (dor e severidade de sintomas) serão avaliados antes da intervenção (baseline), na semana 6 (metade da intervenção), na semana 12 (final da intervenção) e 6 meses após a intervenção. Os demais desfechos serão avaliados no baseline e ao final da intervenção. Os resultados serão avaliados utilizando-se o princípio da análise por intenção de tratar e serão comparados por análises de variância de modelo misto (ANOVA 2-way) com medidas repetidas (grupo x tempo) e o post hoc de Bonferroni.

**Palavras-chave:** Tendão de Aquiles; tendinite; carga, fisioterapia, biomecânica.

**ABSTRACT**

**Introduction:** Achilles tendinopathy has a high incidence in the general population and an even higher incidence in active individuals. Among runners, it is one of the most frequent health conditions, causing a great impact on the quality of life of these individuals. Despite its importance, the current treatment options lead to insufficient results in 5-10 year follow-ups. **Objective:** The primary objective of the study will be to investigate the short-term (6 and 12 weeks) and long-term (6 months) effects of a moderate load resistance exercise intervention [55% of 1 Repetition Maximum (55%1RM)] in comparison to high-load resistance exercise (90% of 1RM) in runners with Achilles tendinopathy in terms of pain and symptom severity. **Methods**: Sixty amateur runners will be randomly allocated into two groups: Group of high intensity exercises (GHI) and group of moderate intensity exercises (GMI). The GHI will start the treatment with triceps surae exercises with 55%1RM in the first and second week, 65%1RM in the third and fourth week, 75%1RM in the fifth and sixth week, 85%1RM in the seventh and eighth week and, from the ninth week until the end of the 12 weeks of treatment, you will perform the exercises with 90%1RM. The GMI will perform exercises with 55%1RM during the 12 weeks of intervention. The RM load will be reassessed every 2 weeks and the total training volume will be identical in both groups. Both groups will do strengthening exercises for quadriceps and gluteal muscles and will continue to perform physical/sports activities using the pain monitoring model strategy. Pain (VAS) and symptom severity (VISA-A), maximum isometric strength of ankle, knee, and hip muscles (handheld dynamometer), function of plantar flexors (functional tests), quality of life (SF-12) and perception of improvement (GROC). Primary outcomes (pain and symptom severity) will be evaluated before the intervention (baseline), at week 6, at week 12 (end of intervention) and 6 months after the intervention. The other outcomes will be evaluated at baseline and at the end of the intervention. Results will be analyzed using the intention-to-treat principle and will be compared by mixed-model analysis of variance (2-way ANOVA) with repeated measures (group x time) and Bonferroni post hoc.

**Keywords:** Achilles tendon; tendinitis; load; physiotherapy, biomechanics.

**INTRODUÇÃO**

A tendinopatia da porção média do tendão de Aquiles apresenta uma incidência entre 2 e 3 pessoas a cada 1.000 indivíduos na população em geral e é ainda mais frequente na população ativa (DE JONGE et al., 2011). Em um estudo coorte com 3379 participantes realizado entre corredores recreativos a incidência de tendinopatia do Aquiles foi de 4,2% e destes, 64% apresentaram tendinopatia de Aquiles na porção média (CHEN et al., 2023).

Além de ser considerada, entre corredores, a condição de saúde mais frequente, sua incidência cumulativa ao longo da vida entre ex-corredores de longa distância é de 52% (KUJALA; SARNA; KAPRIO, 2005). A presença de dor (SLEESWIJK VISSER et al., 2021), incapacidade e severidade dos sintomas (RABUSIN et al., 2021), comprometimento da função muscular (HASANI et al., 2021; MCAULIFFE et al., 2019) e a cinesiofobia (ALGHAMDI et al., 2021; CHIMENTI et al., 2021) são encontrados frequentemente nesses pacientes, o que pode causar afastamento dos atletas de sua atividade esportiva impactando na qualidade de vida (DE VOS et al., 2021; MALLIARAS, 2022; SLEESWIJK VISSER et al., 2021; VAN DER VLIST et al., 2021).

Atualmente as intervenções envolvendo exercícios com progressão de carga gradual para uma adaptação positiva do tendão são consideradas o tratamento de primeira linha para a tendinopatia de Aquiles (DE VOS et al., 2021; ESCRICHE- ESCUDER; CASANÃ; CUESTA-VARGAS, 2020; VAN DER VLIST et al., 2021).

Diversas variações dessa intervenção foram investigadas nas últimas três décadas em diferentes tipos de tendinopatias dos membros inferiores com resultados positivos (ESCRICHE-ESCUDER; CASANÃ; CUESTA-VARGAS, 2020). Podemos destacar as intervenções com exercícios excêntricos (ALFREDSON; PIETILÄ; LORENTZON, 1998), exercícios isométricos (O’NEILL et al., 2019), exercícios de contração concêntrica e excêntrica (Silbernagel et al., 2001) e o treino lento de alta carga (do inglês, *heavy slow resistance training ‒* HSRT) (BEYER et al., 2015; KONGSGAARD et al., 2009).

Apesar de essas intervenções com exercício progressivo serem o padrão ouro para o tratamento da tendinopatia de Aquiles, nem todos os pacientes alcançam a recuperação total, com estudos reportando que entre 20*‒*40% relatam resultados insatisfatórios após 10 anos de tratamento (JOHANNSEN; JENSEN; WETKE, 2018; LAGAS et al., 2022) e apenas 39% estão assintomáticos após 5 anos (VAN DER PLAS et al., 2012). Diante disso, é necessária a busca de outras abordagens de tratamento objetivando uma reabilitação com resultados em longo prazo.

Uma série de estudos tem avaliado a variação de valências do fortalecimento (AGERGAARD et al., 2021; BREDA et al., 2021; STEVENS; TAN, 2014), repetições, séries, frequências e carga, para encontrar uma dose ideal de exercício e magnitude de carga durante as intervenções em pacientes com tendinopatia. Recentemente, Arampatzis et al. (2020) sugerem que, comparando-se programas de exercícios com o mesmo volume, mas com cargas diferentes, somente protocolos com maior carga produzem adaptações nos tendões, como aumento da rigidez e aumento da área transversal em pessoas com tendões de Aquiles saudáveis.

Sabe-se que, em pessoas com tendões de Aquiles saudáveis, exercícios de carga moderada [(55% de 1 repetição máxima (RM)] provocam melhora na força de flexão plantar e hipertrofia muscular do tríceps sural, mas não causam adaptações no tendão de Aquiles (ARAMPATZIS; KARAMANIDIS; ALBRACHT, 2007; ARAMPATZIS; MERSMANN; BOHM, 2020; BOHM et al., 2014). Acredita-se que isso ocorra porque contrações dessa magnitude não provocam deformação (*strain*) do tendão dentro da faixa que é considerada ideal (entre 4,5 e 6%) para haver adaptações mecânicas e morfológicas positivas. Em contrapartida, foi demonstrado que exercícios de alta carga (90% de 1 RM), além de melhorar a força e gerar hipertrofia, conseguem provocar um estímulo mecânico efetivo no tendão de Aquiles que estimulam adaptações benéficas no tecido, causando melhora nas propriedades mecânicas de tendões de Aquiles saudáveis (ARAMPATZIS; KARAMANIDIS; ALBRACHT, 2007; ARAMPATZIS; MERSMANN; BOHM, 2020; BOHM et al., 2014;LAZARCZUK et al., 2022). No entanto, o efeito de exercícios com diferentes magnitudes de cargas em indivíduos com tendinopatia de Aquiles permanece desconhecido.

Os objetivos primários do estudo serão verificar os efeitos a curto (6 e 12 semanas) e a longo prazo (6 meses) de uma intervenção de exercícios resistidos de carga moderada (55%RM) em comparação a exercícios resistidos de alta carga (90%RM) em corredores amadores com tendinopatia de Aquiles em termos de dor e severidade de sintomas. Os objetivos secundários serão verificar o efeito das intervenções na função muscular e na qualidade de vida de corredores com tendinopatia de Aquiles.

**MÉTODO**

**Desenho do estudo**

Este é um protocolo de ensaio controlado randomizado e cego com grupos paralelos (1:1) comparando dois programas de exercícios para corredores com tendinopatia de Aquiles. O trabalho será realizado no Departamento de Fisioterapia da Universidade Federal do Rio Grande do Norte (UFRN), e seguirá as diretrizes do *Standard Protocol Items: Recommendations for Interventional Trials* (SPIRIT) (CHAN et al., 2013). Os participantes serão randomizados em dois grupos: o grupo de alta intensidade (GAI) e o grupo de moderada intensidade (GMI). Esse ensaio clínico será reportado de acordo com as diretrizes do *Consolidated Standards of Reporting Trials* (CONSORT) (SCHULZ; ALTMAN; MOHER, 2010) e o protocolo será registrado na plataforma de Registro Brasileiro de Ensaios Clínicos (ReBEC).

**Critérios de elegibilidade**

Serão incluídos corredores amadores com 18 a 60 anos do sexo masculino, com diagnóstico clínico de tendinopatia da porção média do tendão de Aquiles. O diagnóstico seguirá os seguintes critérios: dor localizada na região da porção média do tendão de Aquiles (de dois a seis centímetros acima da inserção do tendão no calcâneo), dor durante a corrida, espessamento do tendão, rigidez matinal e dor à palpação (DE VOS et al., 2021; HABETS et al., 2021).

Serão excluídos indivíduos que apresentarem (1) tendinopatia de Aquiles exclusivamente insercional, (2) período de *washout* inferior a 4 semanas de outros tratamentos, (3) uso de injeções de corticosteroides na região do tendão de Aquiles ou uso de antibióticos da classe das fluoroquinolonas nos últimos 12 meses (LEWIS; COOK, 2014), (4) outras lesões do membro inferior afetado nos últimos 3 meses, (5) cirurgia musculoesquelética na coluna ou membros inferiores nos últimos 12 meses,

(6) história de ruptura do tendão de Aquiles e (7) doenças sistêmicas que possam interferir na reabilitação (por exemplo, artrite reumatoide ou diabetes) (HABETS et al., 2021).

O recrutamento da amostra será realizado através da pesquisa ativa de cadastros dos grupos de corridas ativos na cidade de Natal, Rio Grande do Norte (Brasil). Serão realizadas divulgações pela internet (Instagram, WhatsApp, Telegram, Facebook) e nos locais tradicionais de corrida na cidade, através de banners. Os corredores que apresentem dor no tendão de Aquiles serão convidados para avaliação clínica de possível elegibilidade.

Para fins de caracterização da amostra, o nível de atividade física de cada participante será avaliado utilizando-se a versão curta do *International Physical Activity Questionnaire-Short Form (IPAQ-SF)*. Trata-se da versão validada para o português do Brasil (PINTO et al, 2005), de um questionário composto por oito questões que permitem estimar o tempo despendido durante uma semana em diferentes dimensões de atividade física (caminhadas e esforços físicos de intensidades moderada e vigorosa) e de inatividade física (posição sentada) (**ANEXO 1**). Será realizado o produto entre a duração (minutos/dia) e a frequência (dias/semana) dessas atividades relatadas pelos participantes (MATSUDO et al., 2001).

Além disso, para a avaliação da cinesiofobia, a versão brasileira do questionário *Tampa Scale of Kinesiophobia (TSK)* será utilizada (SIQUEIRA; TEIXEIRA-SAMELA; MAGALÃES, 2007). A escala consiste em 17 questões que avaliam cinesiofobia e a intensidade dos sintomas (**ANEXO 2**). O escore final varia de 17 a 68 pontos, sendo que, quanto maior a pontuação, maior a cinesiofobia (SIQUEIRA; TEIXEIRA-SAMELA; MAGALÃES, 2007; VLAEYEN et al., 1995).

**Cálculo amostral**

Para determinar o tamanho da amostra do estudo, foi feito o cálculo considerando um nível de confiança (α) de 95%, um poder (β) de 80%, e uma diferença clinicamente importante (MCID) no *Victorian Institute of Sport Assessment- Achilles* (VISA-A) com um valor de 14 pontos (LAGAS et al., 2021). O desvio padrão foi obtido através do ensaio clínico semelhante que usou essa mesma variável como desfecho primário (HABETS et al., 2021). Usando essas informações, foi calculado um tamanho da amostra de 54 indivíduos, sendo 27 para cada grupo. Levando em consideração uma perda de 10%, chegou-se a um número total de 60 indivíduos, sendo 30 em cada grupo.

**Randomização e estratégias para minimização de viés**

A randomização dos participantes será gerada utilizado um sistema de randomização amostral (bloco de quatro) criando uma lista de números aleatórios

([http://www.randomization.com](http://www.randomization.com/)). O responsável pela geração da lista será um pesquisador independente que não estará envolvido com as avaliações, intervenções ou análise dos dados. A partir desta lista, será criado um conjunto de envelopes opacos que serão selados, garantindo o sigilo de alocação.

As intervenções serão realizadas no Departamento de Fisioterapia da Universidade Federal do Rio Grande do Norte (UFRN) e/ou academias de ginástica locais e serão supervisionadas por fisioterapeutas experientes. O investigador principal permanecerá cego quanto aos grupos e realizará todas as avaliações do estudo. Para seu cegamento, ele não participará das intervenções e os participantes serão orientados a não comentarem qual tipo de intervenção irão realizar nem com os outros participantes nem com o avaliador.

**Avaliações**

***Desfechos primários***

Os desfechos primários do presente estudo serão a dor e a severidade dos sintomas dos participantes. Os desfechos primários serão avaliados antes das intervenções (baseline), na semana 6 (metade das intervenções), na semana 12 (final das intervenções) e 6 meses após o fim das intervenções.

Para avaliar a incapacidade e severidade dos sintomas no tendão de Aquiles será utilizado o questionário *Victorian Institute of Sport Assessment-Achilles* (VISA- A) em sua versão traduzida e validada para português do Brasil (DE MESQUITA et al., 2018) (**ANEXO 3**). O VISA-A é uma ferramenta válida, confiável e de fácil aplicação para medir resultados em estudos de intervenção em tendão de Aquiles (MARTIN et al., 2018; ROBINSON et al., 2001). O questionário consiste em oito questões sobre dor e função durante a vida diária e atividades esportivas. A pontuação final fica entre 0 e 100, onde pontuações mais altas representam menor dor e menor incapacidade. Melhoras maiores do que 14 pontos no VISA-A são consideradas clinicamente significativas (LAGAS et al., 2021).

Para avaliação da dor será utilizada a escala visual analógica (EVA), onde 0 representa nenhuma dor e 10 representa a pior dor imaginável (PRICE et al., 1983). O participante será questionado sobre a pior dor na última semana (HABETS et al., 2021), a dor durante o teste de elevação na ponta do pé e o teste de salto unipodal vertical máximo (SILBERNAGEL et al., 2011; HANLON et al., 2021). Diminuições ≥ 3 pontos da escala são considerados clinicamente relevantes (CHIMENTI et al., 2021).

***Desfechos secundários***

Os desfechos secundários incluirão a força isométrica máxima de extensão do quadril e do joelho e de flexão plantar do tornozelo, a função dos músculos flexores plantares, a qualidade de vida e a percepção de melhora ou piora do quadro dos participantes. Os desfechos secundários serão avaliados antes das intervenções (baseline) e imediatamente após as 12 semanas das intervenções.

Para a medida da força isométrica máxima de extensão do quadril, extensão do joelho e flexão plantar do tornozelo usaremos um dinamômetro portátil (Lafayette Instruments, IN, EUA). Além disso, cintas inelásticas serão utilizadas para estabilização dos participantes e na fixação do dinamômetro, para eliminar o efeito da força do avaliador nos testes (SCATTONE SILVA et al., 2016).

A força de extensão do quadril será avaliada com o participante em decúbito ventral com o quadril em posição neutra nos 3 planos e o joelho em 90° de flexão. Uma cinta fixada à maca passará ao redor da pelve do participante para estabilização. O dinamômetro será posicionado imediatamente proximal a fossa poplítea do membro avaliado e será fixado por uma segunda cinta inelástica presa a maca. Será permitido que o participante use as extremidades superiores para estabilizar o tronco na maca (SCATTONE SILVA et al., 2016). O participante receberá a orientação de “fazer força tentando mover o pé em direção ao teto” (**FIGURA 1a**).

A força de extensão do joelho será avaliada com o participante em decúbito dorsal com o joelho flexionado a 30°. Será usado um rolo não deformável abaixo do joelho na altura da fossa poplítea com o intuito de manter essa angulação. Será usada uma cinta inelástica fixada à maca que passará ao redor dos maléolos lateral e medial para a fixação do dinamômetro manual à frente do tornozelo, no ponto médio entre os maléolos. O participante será orientado a cruzar os membros superiores sobre o tronco e “empurrar tentando estender o joelho” (**FIGURA 1b**) (SCATTONE SILVA et al., 2016)

A força de flexão plantar do tornozelo será avaliada com o participante em decúbito ventral com as articulações do membro inferior avaliado em posição neutra.O dinamômetro manual será posicionado na face plantar da cabeça dos metatarsos fixado por uma cinta inelástica que passará ao redor da maca. Será dada a orientação para que o participante “faça força máxima para levar a ponta do pé para baixo” (**FIGURA 1c**) (SCATTONE SILVA et al., 2016).

Em todas as avaliações de força isométrica máxima serão realizadas quatro repetições. A primeira repetição servirá para familiarização do participante ao teste e as três repetições seguintes serão utilizadas para a análise dos dados. Em cada repetição o participante será orientado a manter a força máxima por 5 segundos e entre cada repetição haverá um descanso de 15 segundos (SCATTONE SILVA et al., 2016).


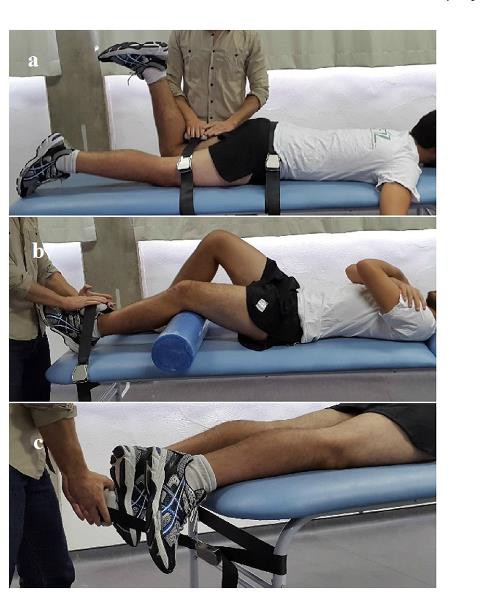


**Figura 1**: Posição de teste para avaliação do torque de extensão do quadril (a), torque extensor do joelho (b) e torque flexor plantar do tornozelo (c).

Para a avaliação da função dos músculos flexores plantares serão realizados dois testes, o teste de elevação na ponta do pé (*single-leg heel rise test)* e o teste de salto unipodal vertical máximo (*single-leg drop vertical jump test)* (SILBERNAGEL et al., 2006). O teste de elevação na ponta do pé avalia o número de elevações na ponta do pé que o participante consegue realizar em apoio unipodal (**FIGURA 2**). Para esse teste, o participante será posicionado em apoio unipodal sobre uma rampa com inclinação de 10° com um marcador de 24mm abaixo do maléolo lateral. Em seguida, será orientado a usar o apoio de dois dedos na parede, na altura dos ombros, para manter o equilíbrio. Será utilizado um metrônomo para manter a frequência de 30 elevações na ponta do pé por minuto. Para a medida do trabalho total da atividade usaremos o aplicativo Calf Raise (FERNANDEZ et al., 2023). O participante será instruído a subir o mais alto possível em cada elevação do calcanhar e, em seguida, abaixar o calcanhar até a posição inicial mantendo joelhos estendidos e o tronco reto. O teste será finalizado quando o participante não conseguir continuar, não conseguir manter a frequência ou não realizar uma elevação completa do calcanhar (SILBERNAGEL et al., 2006).


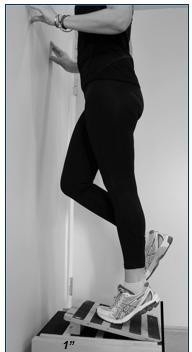
**Figura 2:** Teste de elevação na ponta do pé

O teste de salto unipodal vertical máximo *(single-leg drop vertical jump)* (**FIGURA 3**) será iniciado com a medida da altura do chão para a ponta dos dedos das mãos do participante com o membro superior elevado, erguendo a mão o mais alto possível (**FIGURA 3A**). Após essa medida, será dado ao participante um papel adesivo (*post-it*) para que ele segure com os dedos e, ao realizar o salto, cole o papel no ponto mais alto que ele conseguir alcançar. Para a realização do salto os participantes serão orientados a ficar de pé sobre uma perna em uma caixa de madeira de 20 cm de altura e serão instruídos a se deixarem cair para o chão e logo em seguida realizar um salto vertical o mais alto possível e neste momento, colar o papel adesivo, possibilitando o registro da altura máxima alcançada com o salto (SILBERNAGEL et al., 2011).


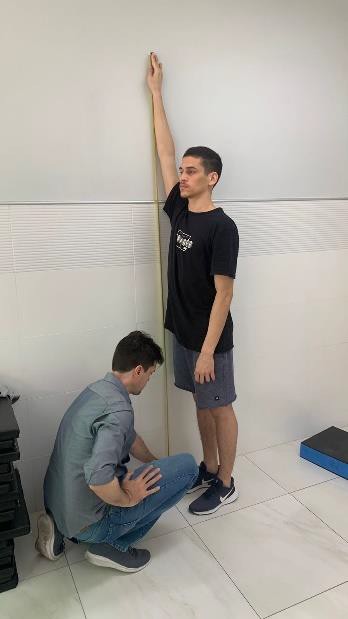

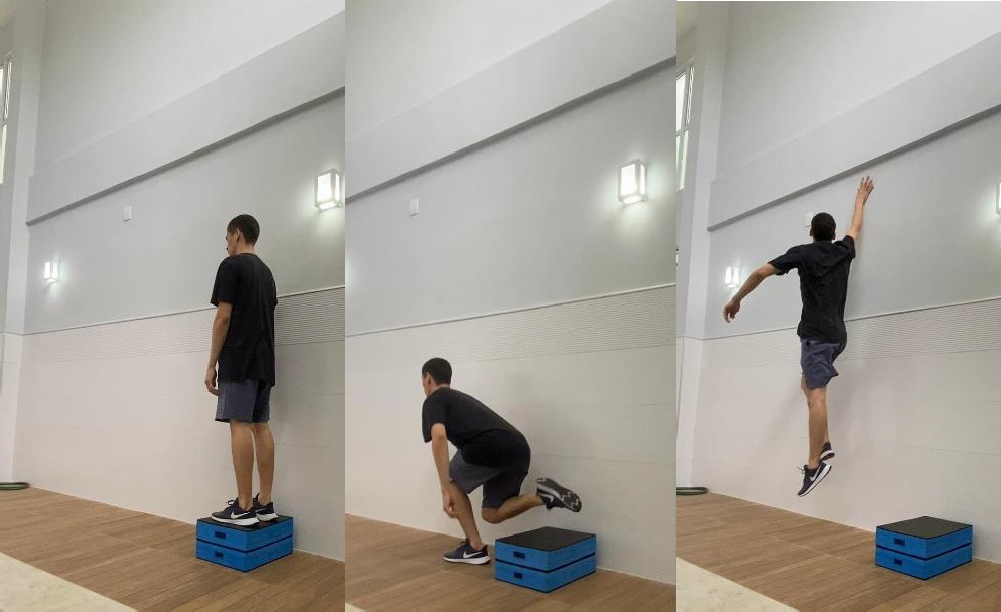


**a**

**b**

**c**

**d**

**Figura 3:** Teste de salto unipodal vertical máximo. medida da altura do chão para a ponta dos dedos das mãos do participante com o membro superior elevado (a); início do salto de uma caixa de 20 cm (b); preparação para o salto vertical com apenas um dos pés (c), salto vertical máximo com apenas um dos pés (d).

Para avaliar o impacto da tendinopatia de Aquiles na qualidade de vida dos participantes utilizaremos a versão validada para o português do Brasil do *Short- Form Health Survey (SF-12)*(CAMALIER, 2004) que consta de 12 questões que representam os 8 domínios do *Form Health Survey*, ele apresenta uma pontuação final de 0 a 100, no qual zero corresponde a pior estado geral de saúde e 100 a melhor estado geral de saúde (**ANEXO 4**) e que pode ser aplicado de forma válida e confiável em um intervalo de tempo menor (WARE; KOSINSKI; KELLER, 1996).

A *Global Rating of Change* (GROC) será utilizada para avaliar a percepção de melhora ou piora do participante após as intervenções (JAESCHKE; SINGER; GUYATT, 1989). Esta escala caracteriza-se por ser uma [do tipo Likert de 15 pontos](https://www.sciencedirect.com/topics/medicine-and-dentistry/likert-scale), variando de -7 (muito pior) a +7 (muito melhor), com 0 indicando nenhuma alteração (**ANEXO 5**). Alterações de 4 pontos ou mais foram previamente consideradas clinicamente importantes em pacientes com dor no tendão de Aquiles (McCormack et al., 2015). O fluxograma do SPIRIT (**FIGURA 4**) ilustra a sequência das atividades que serão desempenhadas durante a realização da pesquisa.

|  | Recrutamento | Alocação | | Pós-alocação | | | | Follow up |
| --- | --- | --- | --- | --- | --- | --- | --- | --- |
| **PERÍODO (SEMANAS)** | **- 1** | **0** |  | **S1/S5** | **S6** | **S7/S11** | **S12** | **S26** |
| Recrutamento | X |  | |  |  |  |  |  |
| Triagem e elegibilidade | X |  | |  |  |  |  |  |
| TCLE |  | X | |  |  |  |  |  |
| Lista de outros procedimentos – medidas de baseline |  | X | |  |  |  |  |  |
| Alocação |  | X | |  |  |  |  |  |
| IPAQ - SF |  | X | |  |  |  |  |  |
| TSK |  | X | |  |  |  | X |  |
| INTERVENÇÂO |  |  | |  |  |  |  |  |
| Grupo de exercícios de alta intensidade (GAI) |  |  | | X | X | X | X |  |
| Grupo de exercícios de intensidade moderada (GMI) |  |  | | X | X | X | X |  |
| AVALIAÇÕES |  |  | |  |  |  |  |  |
| Diagnóstico da tendinopatia do Aquiles | X |  | |  |  |  |  |  |
| Avaliações de baseline |  | X | |  |  |  |  |  |
| VISA–A |  | X | |  | X |  | X | X |
| EVA |  | X | |  | X |  | X | X |
| Força isométrica máxima |  | X | |  |  |  | X |  |
| Função dos flexores plantares |  | X | |  |  |  | X |  |
| SF-12 |  | X | |  |  |  | X |  |
| GROC |  | X | |  |  |  | X |  |

**Figura 4:** Fluxograma do SPIRIT – TCLE, termo de consentimento livre e esclarecido; IPAQ-SF, International Physical Activity Questionnaire; TSK, Tampa Scale of Kinesiophobia; VISA-A, Victorian Institute of Sport Assessment-Achilles; EVA, Escala visual analógica; SF-12, Short-Form Health Survey; GROC, Global Rating of Change.

**Intervenções**

As intervenções ocorrerão durante 3 dias na semana por doze semanas. Pelo menos uma das sessões de tratamento semanais serão realizadas de forma individual e supervisionada. As supervisões serão realizadas presencialmente por fisioterapeutas com experiência na área de ortopedia que passarão por um treinamento específico sobre as atividades desenvolvidas no ensaio clínico. Para as outras duas sessões semanais, os participantes receberão um material com orientações sobre quais os exercícios deverão realizar durante as sessões sem supervisão presencial e como deverão realizar o monitoramento da dor.

Os participantes serão orientados a não realizar atividades físicas que sobrecarreguem os tendões durante as primeiras 3 semanas de tratamento (HABETS et al., 2021). Após esse período, eles serão liberados para a prática de atividade esportiva seguindo o modelo de monitoramento de dor de Silbernagel et al., (2007). O modelo de monitoramento da dor (**FIGURA 5**) estabelece que durante atividades de vida diária, atividades laborais, físicas e esportivas é aceitável um nível de dor que chegue até 5 em uma escala de dor de 0 a 10, contanto que nas próximas 24h após a atividade, os sintomas diminuam (SILBERNAGEL; CROSSLEY, 2015). Nesse contexto, os participantes serão orientados a evitar atividades que gerem dor maior do que 5/10 (HABETS et al., 2021; SILBERNAGEL et al., 2007; SILBERNAGEL; CROSSLEY, 2015)

Os participantes serão divididos em dois grupos que terão volumes totais de exercícios equivalentes para o tríceps sural e o tendão de Aquiles: o grupo de alta intensidade (GAI) que iniciará os exercícios de tríceps sural com 55%1RM na primeira e segunda semana, 65%1RM na terceira e quarta semana, 75%1RM na quinta e sexta semana, 85%1RM na sétima e oitava semana e, a partir da nona semana até o final das 12 semanas de tratamento, realizará os exercícios com 90% de 1RM; e o grupo de moderada intensidade (GMI) que realizará exercícios de

tríceps sural com 55% de 1 RM durante todas as 12 semanas de intervenção (**TABELA 1**):

**Tabela 1:** Protocolos de exercícios de tríceps sural de ambos os grupos

|  | **Sem**  **1-2** | **Sem**  **3-4** | **Sem**  **5** | **Sem**  **6** | **Sem**  **7** | **Sem**  **8** | **Sem 9-12** | **Nº Total de Repetições** |
| --- | --- | --- | --- | --- | --- | --- | --- | --- |
| **GAI** |  |  |  |  |  |  |  |  |
| **% de 1RM** | 55 | 65 | 75 | 75 | 85 | 85 | 90 |  |
| **Nº repetições** | 15 | 15 | 10 | 8 | 8 | 6 | 4 | 1.054 |
| **Nº de séries** | 3 | 3 | 3 | 4 | 4 | 4 | 5 |  |
| **GMI** |  |  |  |  |  |  |  |  |
| **% de 1RM** | 55 | 55 | 55 | 55 | 55 | 55 | 55 |  |
| **Nº repetições** | 17 | 15 | 12 | 10 | 8 | 7 | 6 | 1.053 |
| **Nº de séries** | 3 | 3 | 3 | 3 | 3 | 3 | 3 |  |

Sem, semana; GAI, grupo de alta intensidade; GMI, grupo de moderada intensidade; RM, Repetição máxima.

Além de exercícios específicos para o tríceps sural, ambos os grupos receberão exercícios de fortalecimento para músculos da cadeia cinética, sinergistas do tríceps sural na corrida, e/ou que frequentemente apresentam déficits de força em indivíduos com tendinopatia de Aquiles (HABETS et al., 2021). Esses exercícios serão realizados seguindo as recomendações para o treino de força para pessoas treinadas do *American College of Sports Medicine (ACSM)* (RATAMESS et al., 2009) e para garantir o ajuste da carga desses exercícios faremos uso da “regra do mais dois” onde a última série do exercício deve ser realizada com o máximo de repetições possíveis e caso o participante consiga realizar pelo menos duas repetições extras ao que foi definido, a carga deve ser aumentada na próxima sessão de treinamento (STENSRUD; ROOS; RISBERG, 2012).

Antes da execução de todos os exercícios, os participantes irão realizar uma série de aquecimento, realizando o próprio exercício sem carga, antes de iniciar a execução proposta pelo protocolo de intervenção. A cada duas semanas será realizado um teste submáximo de 10-RM para estimar 1 RM e ajustar as cargas de treinamento adequadamente (BRZYCKI, 1993; REYNOLDS et al 2006). Em exercícios onde a resistência se dará por faixa elástica, será estabelecido como carga inicial dois níveis de resistência elástica inferior à do 1RM e a progressão na carga se dará por meio do aumento de 1 nível de resistência elástica (BALDON et al., 2014).

O protocolo de exercícios de ambos os grupos será dividido em fases distintas, incluindo fortalecimentos progressivos lentos (3 segundos para a fase concêntrica e 3 segundos para a fase excêntrica), exercícios pliométricos e fortalecimentos específicos da corrida. A descrição das fases é apresentada a seguir.

**FASE 1 (do início da intervenção até o final da quarta semana)**

***Elevação do calcanhar na ponta dos pés bipodal sentado***

O exercício de elevação do calcanhar na ponta dos pés bipodal sentado inicia com o participante sentado, na ponta de uma cadeira, sem encostar no apoio para as costas. com o quadril e o joelho em 90° de flexão (**FIGURA 6**). Será solicitado que o participante realize a elevação do calcanhar máxima mantendo o apoio no antepé, em seguida descendo até a dorsiflexão máxima. A resistência será colocada na região anterior distal da coxa, acima da patela (BAXTER et al., 2021; CHIMENTI et al., 2023).


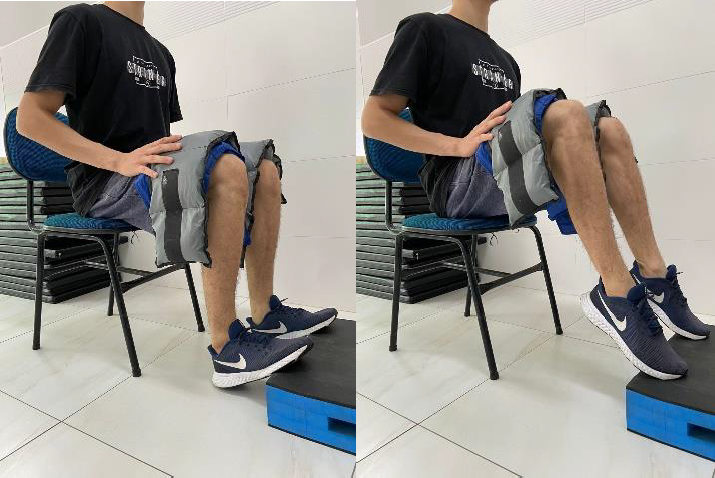


**a**

**b**

**Figura 5:** Posição inicial do exercício de elevação do calcanhar na ponta dos pés bipodal sentado saindo da dorsiflexão máxima do tornozelo (a); Posição final do exercício de elevação na ponta dos pés bipodal sentado terminando em flexão plantar máxima do tornozelo (b).

***Elevação do calcanhar na ponta dos pés bipodal***

O exercício de elevação do calcanhar na ponta dos pés bipodal inicia com o paciente de pé com o antepé na ponta de um degrau, podendo usar as mãos para apoiar em uma parede, evitando desequilíbrio durante a realização do exercício. Os pés devem estar separados de acordo com a largura do ombro (**FIGURA 7**). O participante será orientado a ficar na ponta dos pés elevando o calcanhar da máxima dorsiflexão até a máxima flexão plantar. A resistência será colocada na altura dos ombros por meio de um colete com peso (BAXTER et al., 2021; CHIMENTI et al., 2023).


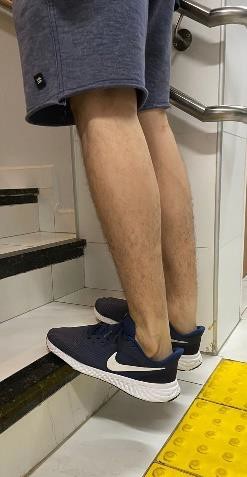

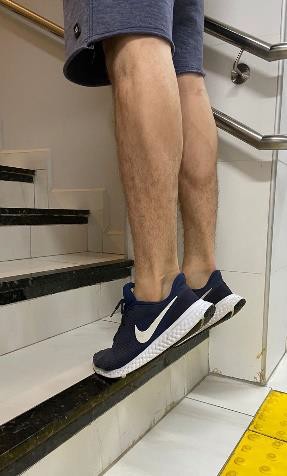


**a**

**b**

**Figura 6:** Elevação do calcanhar na pontas dos pés bipodal: posição inicial em uma dorsiflexão máxima (a); Posição final em uma flexão plantar máxima (b).

***Extensão de joelho na cadeira extensora bipodal***

O exercício de extensão de joelho na cadeira extensora bipodal inicia com o participante sentado em uma máquina de extensão de joelho (cadeira extensora), com os joelhos flexionados a 90° e os quadris flexionados a 100°. O braço de alavanca do equipamento será fixado imediatamente acima dos tornozelos (**FIGURA 8**). O participante será orientado a realizar a extensão total dos joelhos (0° de extensão) e depois retornar à posição inicial (90° de flexão) (JAKOBSEN et al., 2012).


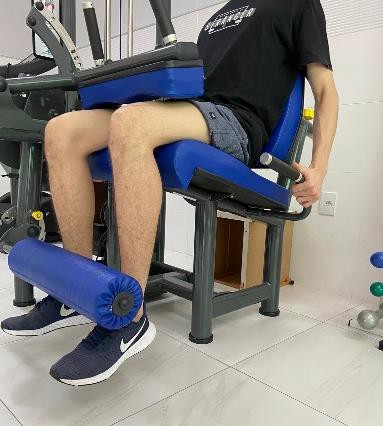

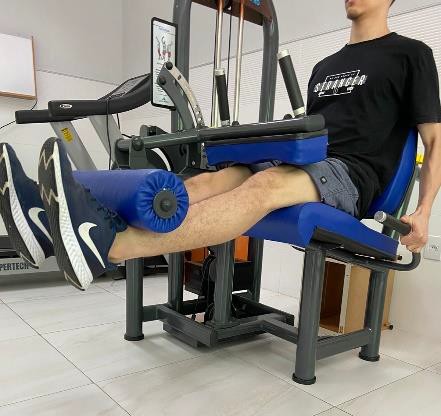


**a**

**b**

**Figura 7:** Posição inicial o exercício de extensão do joelho na cadeira extensora (a); Posição final do exercício de extensão do joelho na cadeira extensora (b).

***Exercício de ostra com 60° de flexão do quadril***

O exercício de ostra inicia com o paciente em decúbito lateral, com o quadril flexionado em 60°, os joelhos flexionados a 90°, com a resistência elástica na altura dos joelhos (**FIGURA 9**). Será solicitado que o participante realize um movimento de abdução com rotação lateral do quadril mantendo o contato dos calcanhares e as espinhas ilíacas anterossuperiores voltadas para a frente e, em seguida, retornar à posição inicial (BISHOP et al., 2018; DISTEFANO et al., 2009).


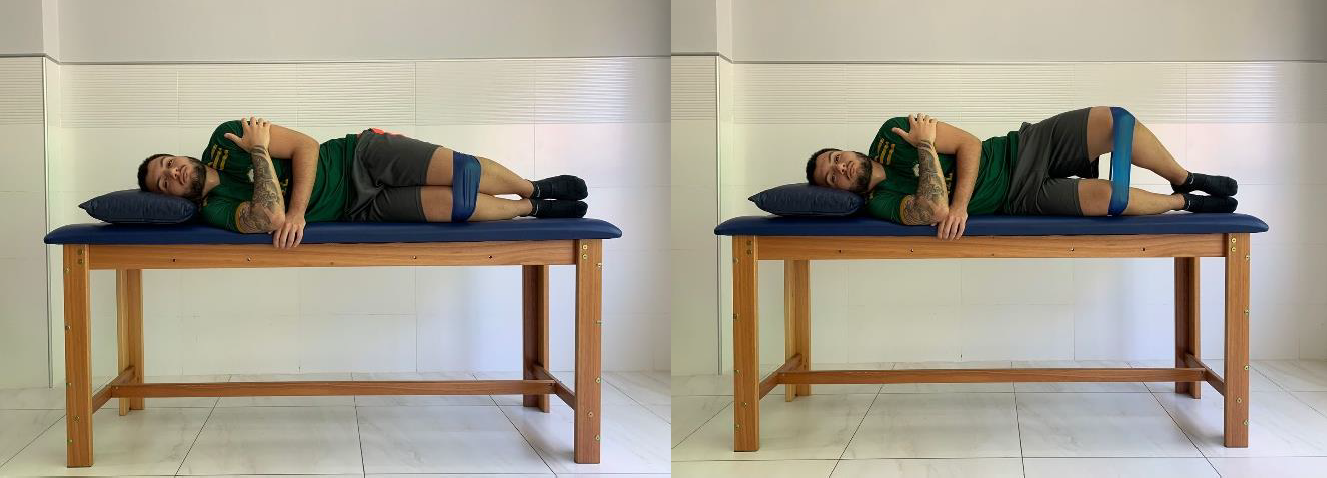


**a**

**b**

**Figura 8:** Posição inicial e final para o exercício de ostra com 60° de flexão do quadril (a); Posição intermediária para o exercício de ostra com 60° de flexão do quadril (b).

**FASE 2 (da 5ª semana até o final da 8ª semana)**

Na fase dois serão realizados os exercícios de elevação do calcanhar na ponta dos pés sentado unipodal e elevação do calcanhar na ponta dos pés unipodal (**FIGURA 10**). A execução desses dois exercícios é idêntica à da Fase 1, exceto por serem realizados em apoio unipodal (BAXTER et al., 2021; CHIMENTI et al., 2023).


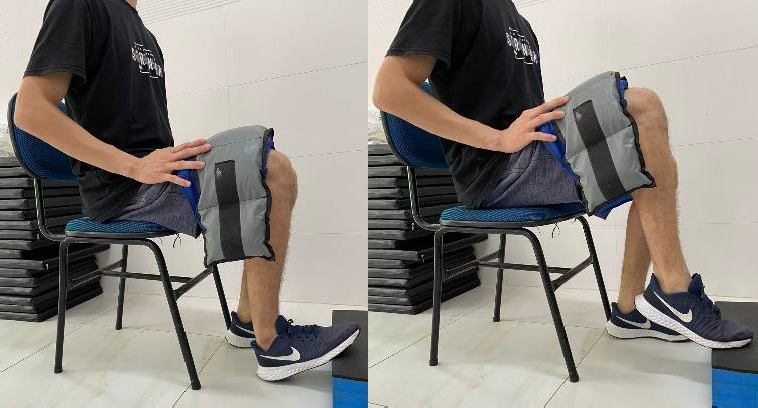

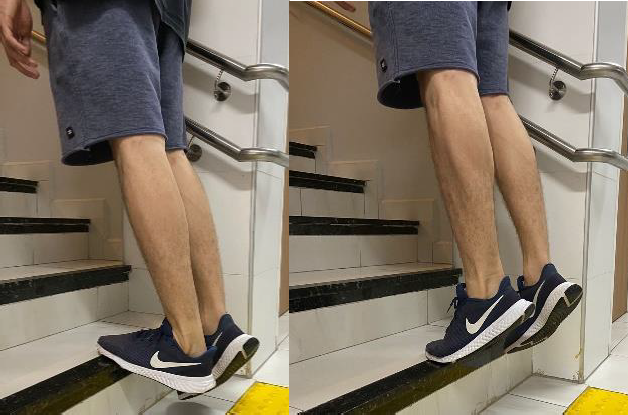


**a**

**b**

**c**

**d**

**Figura 9:** Elevação do calcanhar sentado unipodal: posição inicial em dorsiflexão máxima do tornozelo (a) e posição final em flexão plantar máxima do tornozelo (b). Elevação do calcanhar na ponta do pé unipodal: posição inicial em dorsiflexão máxima do tornozelo (c) e posição final em flexão plantar máxima do tornozelo (d).

***Extensão de joelho na cadeira extensora unipodal***

O exercício de extensão de joelho na cadeira extensora unipodal (**FIGURA 11**) inicia com o participante sentado em uma máquina de extensão de joelho (cadeira extensora), com o joelho flexionado a 90° e o quadril flexionado a 100°. O braço de alavanca do equipamento será fixado imediatamente acima do tornozelo. O participante será orientado a realizar a extensão total do joelho (0° de extensão) e depois retornar à posição inicial (90° de flexão) (JAKOBSEN et al., 2012).


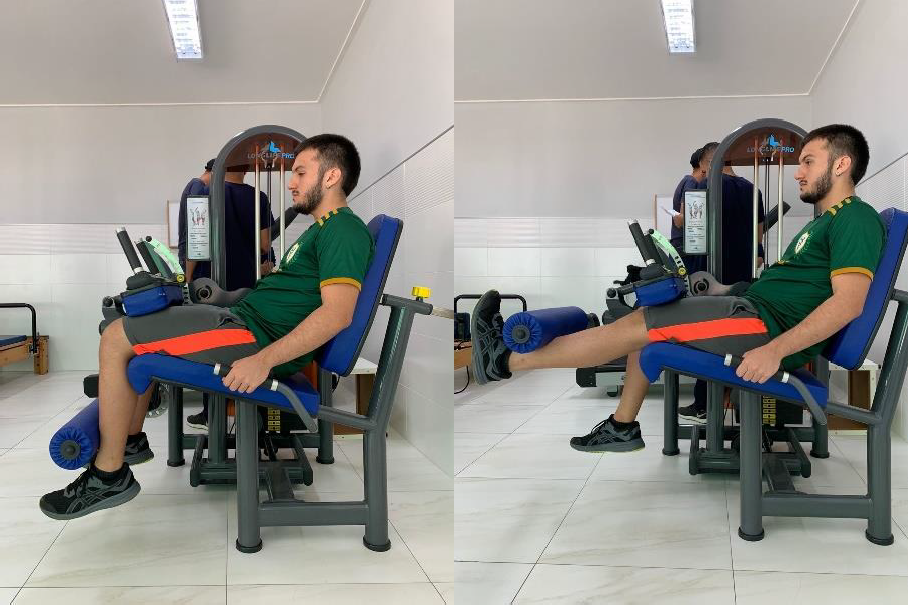


**a**

**b**

**Figura 10:** Posição inicial para o exercício de extensão do joelho na cadeira extensora (a). Posição final para o exercício de extensão do joelho na cadeira extensora (b).

***Deadlift unipodal***

O exercício de *deadlift* unipodal (**FIGURA 12**) inicia-se com o indivíduo apoiado no membro acometido com o joelho fletido a aproximadamente 30° e as mãos estarão segurando halteres que servirão de resistência. Em seguida, o indivíduo será orientado a flexionar o quadril e o tronco lentamente até alcançar 90° de flexão do quadril e depois retornar à posição inicial. Os participantes serão instruídos a manter o joelho flexionado a 30° ao alcançar o nível desejado, para permitir principalmente a flexão do tronco e do quadril e manter os joelhos sobre os artelhos (COLLINGS et al., 2023; DISTEFANO et al., 2009) .


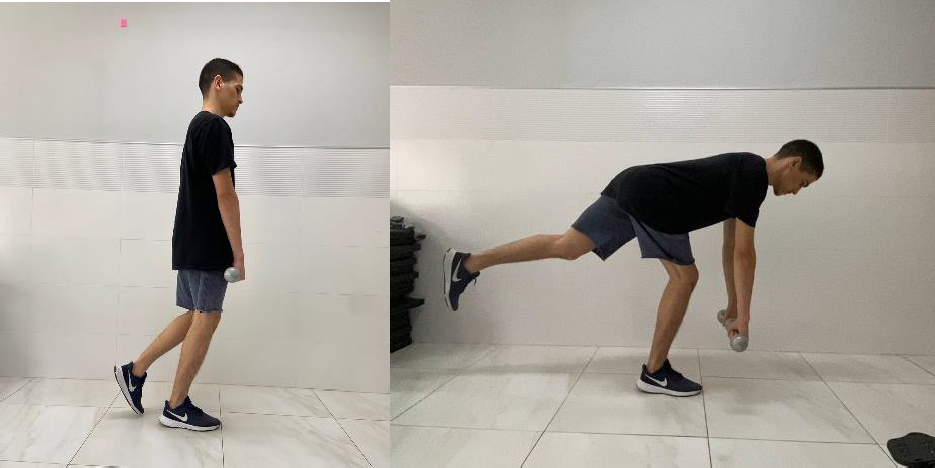


**a**

**b**

**Figura 11:** Exercício de *deadlift* unipodal: Posição inicial (a), posição final (b).

**FASE 3 (da 9ª semana até o final da 12ª)**

Na fase 3 os participantes realizarão exercícios pliométricos, descritos abaixo, no dia da sessão supervisionada e nas outras duas sessões semanais realizarão os mesmos exercícios da fase 2.

***Saltos bipodais rápidos***

O exercício de saltos bipodais rápidos (**FIGURA 13**) inicia com o paciente de pé, com os dois pés no chão afastados na largura dos quadris e membros superiores relaxados. Será solicitado que ele realize pequenos saltos rápidos, de forma que ele sempre retire os dois pés do chão e aterrisse, em seguida, também com os dois pés sem deixar o calcanhar tocar no chão e sem utilizar resistência externa. A resistência se dará apenas pelo próprio peso do corpo. Serão realizadas 3 séries de 20 segundos e serão acrescentados 5 segundos a cada sessão que o participante conseguir atingir a meta (CHIMENTI et al., 2023) .


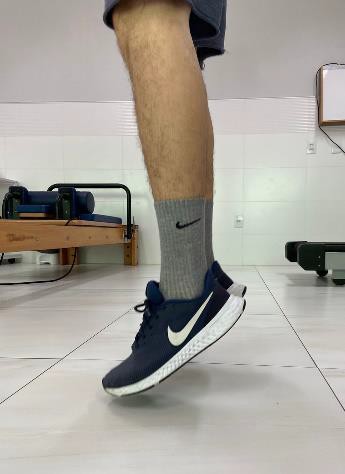


**Figura 12:** Saltos bipodais rápidos.

***Saltos unipodais alternados rápidos***

O exercício de saltos unipodais alternados rápidos (**FIGURA 14**) inicia com o paciente de pé, com os dois pés no chão afastados na largura dos quadris e membros superiores relaxados. Para esse exercício, será solicitado que ele realize pequenos saltos rápidos, de forma que ele retire os dois pés do chão e aterrisse com um pé de cada vez de forma alternada. Serão realizadas 3 séries de 20 segundos e serão acrescentados 5 segundos a cada sessão que o participante conseguir atingir essa meta (CHIMENTI et al., 2023).


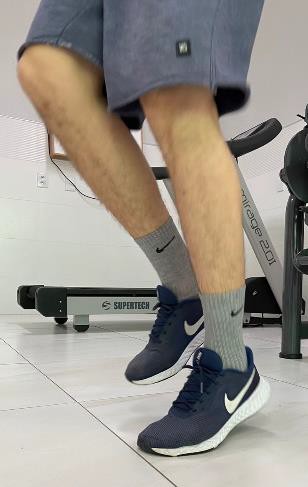


**Figura 13:** Saltos unipodais alternados rápidos.

***Drop vertical jump bipodal***

O exercício de *drop vertical jump* (**FIGURA 15**) inicia com o participante de pé em cima de uma caixa de 20 cm de altura. Eles serão orientados a se deixarem cair para o chão a frente da caixa e imediatamente saltar verticalmente o mais alto possível, aterrissando novamente no mesmo local. O exercício consistirá em 5 séries de 20 saltos, cada série com um minuto de descanso entre elas (ANDO et al., 2021; BAXTER et al., 2021).

***Drop vertical jump unipodal***

O exercício de *drop vertical jump* unipodal (**FIGURA 16**) inicia com o participante de pé em cima de uma caixa de 20 cm de altura. Ele será orientado a se deixar cair no chão a frente da caixa com apenas o pé acometido e imediatamente pular o mais alto possível, aterrissando novamente no mesmo local, com o pé acometido. O exercício consistirá em 5 séries de 20 saltos, cada série com um minuto de descanso entre elas (ANDO et al., 2021; BAXTER et al., 2021).

***Exercício de corrida na parede***

O exercício de corrida na parede (**FIGURA 17)** inicia com o participante de pé, com as mãos apoiadas em uma parede, na largura e na altura dos ombros, com o membro superior com o cotovelo em extensão total. Será pedido para que o participante dê um passo para trás, para que o tronco e o membro inferior fiquem num ângulo de 45° com o solo. Para o exercício, o participante será orientado a retirar um dos pés do solo e adotar a postura do membro inferior em flexão do quadril e joelho de aproximadamente 100°, mantendo o tornozelo que está no solo atrás do quadril em flexão plantar. Em seguida será solicitado que o participante alterne o contato dos pés no solo saltando de uma perna para outra 3 vezes, sem tocar o calcanhar no chão (LIEBENSON, 2009). O exercício é então repetido com a outra perna até que seja completada uma série de 10 repetições. Esse exercício foi selecionado por ser um exercício em que há um recrutamento maior do músculo sóleo durante a flexão plantar com extensão total do joelho, e por ser um movimento similar ao efetuada na corrida (SUZUKI; CHINO; FUKASHIRO, 2014).


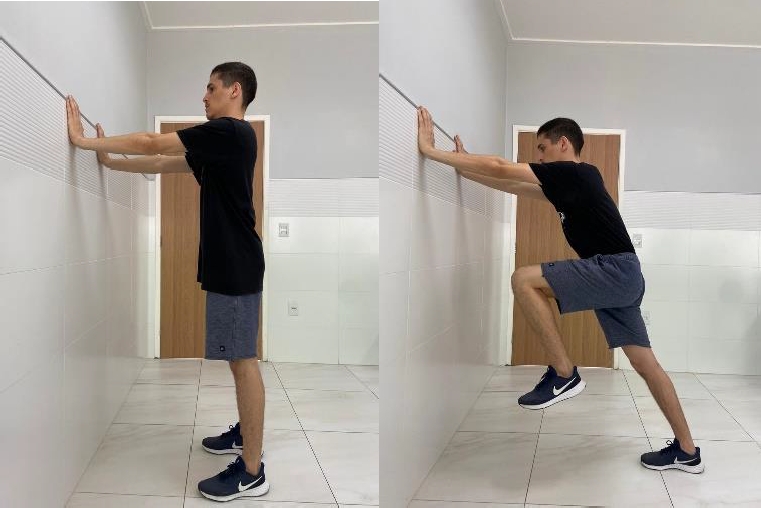


**a**

**b**

**Figura 14:** Exercício de corrida na parede: Posição inicial de pé (a); Posição para iniciar a alternância entre os pés de apoio.

**Monitoramento de efeitos adversos**

No caso de surgimento de efeitos adversos como o surgimento de dor ou edema durante ou após a intervenção, participante será orientado a entrar em contato com um dos fisioterapeutas responsáveis pela orientação da intervenção os quais irão decidir em conjunto sobre as possibilidades de ajustar, interromper temporariamente ou interromper definitivamente a intervenção.

Todos os participantes utilizarão um diário de treinamento para registros sobre as sessões. Os registros de treinamento incluirão informações sobre a intensidade do treinamento (repetições, séries e cargas), sessões, dor durante o treinamento (EVA) e desvios dos protocolos de intervenção planejados (AGERGAARD et al., 2021).

Os participantes serão orientados a não executar nenhum outro tipo de tratamento durante o período do estudo. Se algum medicamento para dor ou recurso analgésico for utilizado durante o período de intervenção, os participantes deverão registrar a quantidade e a dosagem de cada medicamento e os parâmetros do recurso analgésico, relatando essa informação nas avaliações pós-intervenção (DIAS ARAÚJO et al., 2022).

**Riscos e Benefícios**

Durante a realização da pesquisa poderão ocorrer eventuais desconfortos musculares e possíveis riscos como quedas durante a realização dos exercícios com salto ou no degrau. O risco de queda será diminuído com o uso de objetos que servirão de apoio, caso o participante se desequilibre. O pesquisador também estará próximo ao participante durante todas as avaliações e sessões de exercícios presenciais, para ajudá-lo a recuperar o equilíbrio, caso seja necessário. Além disso, há o risco de o participante sentir dor muscular no dia seguinte aos exercícios realizados. Esses riscos poderão ser minimizados pela progressão gradual nas cargas dos exercícios e pelo acompanhamento do fisioterapeuta que orientará o participante na realização adequada dos exercícios durante os encontros e nos materiais de apoio.

Como benefícios do estudo o participante obterá tratamento gratuito para a sua condição de saúde, oferecido por um fisioterapeuta experiente, além de receber informações coletadas como a força dos músculos da perna e resistência dos músculos da panturrilha, que serão disponibilizadas na forma de um relatório. O relatório será disponibilizado no final da pesquisa, após o processamento dos dados, e os resultados serão organizados e enviados para o participante em linguagem de fácil compreensão. Em caso de complicações ou danos à saúde que o participante possa ter relacionado com o estudo, compete ao pesquisador responsável garantir o direito à assistência integral e gratuita, devido a danos diretos ou indiretos, imediatos ou tardios, pelo tempo que for necessário, sendo assegurado pelo pesquisador responsável. Se o participante sofrer algum dano decorrente desta pesquisa terá o direito de solicitar indenização. Além disso, o participante também poderá solicitar reembolso caso haja algum gasto com o a participação na pesquisa.

**Análise de Dados**

Os dados serão analisados por um pesquisador que não terá conhecimentos de qual grupo o participante pertence. Este pesquisador ficará responsável apenas pelas avaliações dos desfechos e pela análise dos dados durante a pesquisa. Para a análise dos dados será utilizado o software *Statistical Package for the Social Science* (versão 17.0; SPSS Inc, Chicago, IL) para as análises dos resultados. Para a avaliação da normalidade ou não das distribuições dos dados e a homocedasticidade serão utilizados os testes de Shapiro-Wilk e Levene, respectivamente. As medidas de resultados serão analisadas usando análise de variância de modelo misto (ANOVA two-way) (grupo x tempo) com medidas repetidas com testes post hoc ajustados de Bonferroni quando apropriado. Os resultados serão apresentados como média ± erro padrão da medição (SEM) e intervalo de confiança (IC) de 95%. A esfericidade será verificada utilizando-se o teste de Mauchly e se a esfericidade for violada, a correção de Greenhouse-Geisser será utilizada.

Todos os participantes podem escolher abandonar o estudo a qualquer momento, caso isso aconteça os dados serão analisados utilizando-se o princípio de análise com intenção de tratar (KAMPER, 2021).

**Ética e Divulgação**

O projeto será submetido ao Comitê de Ética em Pesquisa da Universidade Federal do Rio Grande do Norte e os participantes assinarão o Termo de Consentimento Livre e Esclarecido. Os resultados serão divulgados através de publicações em revistas científicas, redes sociais e apresentações em eventos científicos.

**REFERÊNCIAS**

AGERGAARD, A. S. et al. Clinical Outcomes, Structure, and Function Improve With Both Heavy and Moderate Loads in the Treatment of Patellar Tendinopathy: A Randomized Clinical Trial. **American Journal of Sports Medicine**, v. 49, n. 4, p. 982–993, 1 mar. 2021.

ALFREDSON, H.; PIETILÄ, T.; LORENTZON, R. Heavy-Load Eccentric Calf Muscle Training For the Treatment of Chronic Achilles Tendinosis. **The American Journal of Sports Medicine**, v. 26, n. 3, p. 360–366, 1998.

ALGHAMDI, N. H. et al. The Impact of the Degree of Kinesiophobia on Recovery in Patients with Achilles Tendinopathy. **Physical Therapy**, v. 101, n. 11, 1 nov. 2021.

ANDO, R. et al. Relationship Between Drop Jump Training–Induced Changes in Passive Plantar Flexor Stiffness and Explosive Performance. **Frontiers in Physiology**, v. 12, p. 777268, 18 nov. 2021.

ARAMPATZIS, A.; KARAMANIDIS, K.; ALBRACHT, K. Adaptational responses of the human Achilles tendon by modulation of the applied cyclic strain magnitude. **Journal of Experimental Biology**, v. 210, n. 15, p. 2743–2753, ago. 2007.

ARAMPATZIS, A.; MERSMANN, F.; BOHM, S. Individualized Muscle-Tendon Assessment and Training. **Frontiers in Physiology**, v. 11, 26 jun. 2020.

BAXTER, J. R. et al. Exercise Progression to Incrementally Load the Achilles Tendon. **Medicine and Science in Sports and Exercise**, v. 53, n. 1, p. 124–130, 1 jan. 2021.

BEYER, R. et al. Heavy slow resistance versus eccentric training as treatment for achilles tendinopathy: A randomized controlled trial. **American Journal of Sports Medicine**, v. 43, n. 7, p. 1704–1711, 3 jul. 2015.

BISHOP, B. N. et al. Electromyographic Analysis of Gluteus Maximus, Gluteus Medius, and Tensor Fascia Latae During Therapeutic Exercises With and Without Elastic Resistance. **International Journal of Sports Physical Therapy**, v. 13, n. 4,

p. 668–675, ago. 2018.

BOHM, S. et al. Human Achilles tendon plasticity in response to cyclic strain: Effect of rate and duration. **Journal of Experimental Biology**, v. 217, n. 22, p. 4010–4017, 1 nov. 2014.

BREDA, S. J. et al. Effectiveness of progressive tendon-loading exercise therapy in patients with patellar tendinopathy: a randomised clinical trial. **British Journal of Sports Medicine**, v. 55, n. 9, p. 501–509, 1 maio 2021.

BRZYCKI, M. Strength Testing-Predicting a One-Rep Max from Reps-to-Fatigue.

**JOPERD**, v. 64, p. 88–90, jan. 1993.

CAMALIER, A. A. **AVALIAÇÃO DA QUALIDADE DE VIDA RELACIONADA À SAÚDE EM PACIENTES COM DPOC: ESTUDO DE BASE POPULACIONAL COM**

**O SF-12**. São Paulo: Unifesp, 2004.

CHAN, A. W. et al. SPIRIT 2013 Statement: Defining Standard Protocol Items for Clinical Trials. **Annals of internal medicine**, v. 158, n. 3, p. 200, 5 fev. 2013.

CHEN, W. et al. Epidemiology of insertional and midportion Achilles tendinopathy in runners: A prospective cohort study. **Journal of Sport and Health Science**, 23 mar. 2023.

CHIMENTI, R. L. et al. Kinesiophobia Severity Categories and Clinically Meaningful Symptom Change in Persons With Achilles Tendinopathy in a Cross-Sectional Study: Implications for Assessment and Willingness to Exercise. **Frontiers in Pain Research**, v. 2, p. 739051, 1 set. 2021.

CHIMENTI, R. L. et al. The effects of pain science education plus exercise on pain and function in chronic Achilles tendinopathy: a blinded, placebo-controlled, explanatory, randomized trial. **Pain**, v. 164, p. 47–65, 17 jun. 2023.

COLLINGS, T. J. et al. Gluteal Muscle Forces during Hip-Focused Injury Prevention and Rehabilitation Exercises. **Medicine & Science in Sports & Exercise**, v. 55, n. 4,

p. 650–660, abr. 2023.

DE JONGE, S. et al. Incidence of midportion Achilles tendinopathy in the general population. **British Journal of Sports Medicine**, v. 45, n. 13, p. 1026–1028, 1 out. 2011.

BALDON, R. M. et al. Effects of functional stabilization training on pain, function, and lower extremity biomechanics in women with patellofemoral pain: A randomized clinical trial. **Journal of Orthopaedic and Sports Physical Therapy**, v. 44, n. 4, p. 240–251, 2014.

DE MESQUITA, G. N. et al. Cross-cultural adaptation and measurement properties of the brazilian Portuguese version of the victorian institute of sport assessment-achilles (VISA-A) questionnaire. **Journal of Orthopaedic and Sports Physical Therapy**, v. 48, n. 7, p. 567–573, 1 jul. 2018.

DE VOS, R. J. et al. Dutch multidisciplinary guideline on Achilles tendinopathy.

**British Journal of Sports Medicine**, v. 55, n. 20, p. 1125–1134, 1 out. 2021.

DIAS ARAÚJO, E. H. et al. Intervention Treating Kinetic Chain Factors versus Heavy- Slow Resistance Training in Athletes with Patellar Tendinopathy: Protocol for a Randomized Blind Clinical Trial. **Muscle Ligaments and Tendons Journal**, v. 12, n. 03, p. 386, jul. 2022.

DISTEFANO, L. J. et al. Gluteal muscle activation during common therapeutic exercises. **Journal of Orthopaedic and Sports Physical Therapy**, v. 39, n. 7, p. 532–540, 2009.

ESCRICHE-ESCUDER, A.; CASANÃ, J.; CUESTA-VARGAS, A. I. Original research:

Load progression criteria in exercise programmes in lower limb tendinopathy: a systematic review. **BMJ Open**, v. 10, n. 11, 19 nov. 2020.

FERNANDEZ, M. R. et al. Concurrent validity and reliability of a mobile iOS application used to assess calf raise test kinematics. **Musculoskeletal Science and Practice**, v. 63, p. 102711, 1 fev. 2023.

HABETS, B. et al. No Difference in Clinical Effects When Comparing Alfredson Eccentric and Silbernagel Combined Concentric-Eccentric Loading in Achilles Tendinopathy: A Randomized Controlled Trial. **Orthopaedic Journal of Sports Medicine**, v. 9, n. 10, 2021.

HANLON, S. L.; POHLIG, R. T.; SILBERNAGEL, K. G. Beyond the diagnosis: Using patient characteristics and domains of tendon health to identify latent subgroups of achilles tendinopathy. **Journal of Orthopaedic and Sports Physical Therapy**, v. 51, n. 9, p. 440–450, 1 set. 2021.

HASANI, F. et al. Are Plantarflexor Muscle Impairments Present Among Individuals with Achilles Tendinopathy and Do They Change with Exercise? A Systematic Review with Meta-analysis. **Sports Medicine - Open**, v. 7, n. 1, 1 dez. 2021.

JAESCHKE, R.; SINGER, J.; GUYATT, G. H. Measurement of Health Status Ascertaining the Minimal Clinically Important Difference. **Controlled Clinical Trials**,

v. 10, p. 407–415, 1989.

JAKOBSEN, M. D. et al. Muscle activity during knee-extension strengthening exercise performed with elastic tubing and isotonic resistance. **International Journal of Sports Physical Therapy**, v. 7, n. 6, p. 606, dez. 2012.

JOHANNSEN, F.; JENSEN, S.; WETKE, E. 10-year follow-up after standardised treatment for Achilles tendinopathy. **BMJ Open Sport — Exercise Medicine**, v. 4, n. 1, p. 415, 1 out. 2018.

KAMPER, S. J. Per-protocol, intention-to-treat, and complier average causal effects analyses in randomized controlled trials: Linking evidence to practice. **Journal of Orthopaedic and Sports Physical Therapy**, v. 51, n. 6, p. 314–315, 1 jun. 2021.

KONGSGAARD, M. et al. Corticosteroid injections, eccentric decline squat training and heavy slow resistance training in patellar tendinopathy. **Scandinavian journal of Medicine & Science in Sports**, v. 19, p. 790–802, 2009.

KUJALA, U. M.; SARNA, S.; KAPRIO, J. Cumulative Incidence of Achilles Tendon Rupture and Tendinopathy in Male Former Elite Athletes. **Clin J Sport Med**, v. 15, n. 3, p. 133–135, 2005.

LAGAS, I. F. et al. Victorian institute of sport assessment-achilles (visa-a) questionnaire—minimal clinically important difference for active people with midportion achilles tendinopathy: A prospective cohort study. **Journal of Orthopaedic and Sports Physical Therapy**, v. 51, n. 10, p. 510–516, 1 out. 2021.

LAGAS, I. F. et al. One fifth of patients with Achilles tendinopathy have symptoms after 10 years: A prospective cohort study. **Journal of Sports Sciences**, v. 40, p. 2475–2483, 23 dez. 2022.

LAZARCZUK, S. L. et al. Mechanical, Material and Morphological Adaptations of Healthy Lower Limb Tendons to Mechanical Loading: A Systematic Review and Meta-Analysis. **Sports Medicine**, v. 52, n. 10, p. 2405–2429, 1 out. 2022.

LEWIS, T.; COOK, J. Fluoroquinolones and tendinopathy: A guide for athletes and sports clinicians and a systematic review of the literature. **Journal of Athletic Training**, v. 49, n. 3, p. 422–427, 2014.

LIEBENSON, C. Training for speed. **Journal of Bodywork and Movement Therapies**, v. 13, n. 4, p. 362–363, out. 2009.

MALLIARAS, P. Physiotherapy management of Achilles tendinopathy. **Journal of Physiotherapy**, v. 68, n. 4, p. 221–237, 1 out. 2022.

MARTIN, R. L. et al. Achilles pain, stiffness, and muscle power deficits: Midportion achilles tendinopathy revision 2018. **Journal of Orthopaedic and Sports Physical Therapy**, v. 48, n. 5, p. A1–A38, 1 maio 2018.

MATSUDO, S. et al. Questionario internacional de ativi dade fisica (i paq): estudo de vall dade e reprodutibilidade no brasil international physical activity questionnaire (lpaq): study of validity and reliability in brazil. **Atividade física e saúde**, v. 6, n. 2, p. 05–18, 2001.

MCAULIFFE, S. et al. Altered Strength Profile in Achilles Tendinopathy: A Systematic Review and Meta-Analysis. **Journal of Athletic Training**, v. 54, n. 8, p. 889–900, 1 ago. 2019.

MCCORMACK, J. et al. The minimum clinically important difference on the visa‐a and lefs for patients with insertional achilles tendinopathy. **International Journal of Sports Physical Therapy**, v. 10, n. 5, p. 639, out. 2015.

O’NEILL, S. et al. Acute sensory and motor response to 45-s heavy isometric holds for the plantar flexors in patients with Achilles tendinopathy. **Knee Surgery, Sports Traumatology, Arthroscopy**, v. 27, n. 9, p. 2765–2773, 1 set. 2019.

PINTO GUEDES, D.; CORREA LOPES, C.; ELISABETE RIBEIRO PINTO GUEDES,

J. Reprodutibilidade e validade do Questionário Internacional de Atividade Física em adolescentes ARTIGO ORIGINAL. **Rev Bras Med Esporte**, v. 11, n. 2, p. 151–158, 2005.

PRICE, D. D. et al. The Validation of Visual Analogue Scales as Ratio Scale Measures for Chronic and Experimental Pain. **Pain**, v. 17, p. 45–56, 1983.

RABUSIN, C. L. et al. Efficacy of heel lifts versus calf muscle eccentric exercise for mid-portion Achilles tendinopathy (HEALTHY): a randomised trial. **British Journal of Sports Medicine**, v. 55, n. 9, p. 486–492, 1 maio 2021.

RATAMESS, N. A. et al. Progression models in resistance training for healthy adults. **Medicine and Science in Sports and Exercise**, v. 41, n. 3, p. 687–708, mar. 2009. REYNOLDS, J. M.; GORDON, T. J.; ROBERGS, R. A. **PREDICTION OF ONE REPETITION MAXIMUM STRENGTH FROM MULTIPLE REPETITION MAXIMUM**

**TESTING AND ANTHROPOMETRYJournal of Strength and Conditioning Research**. [s.l: s.n.].

ROBINSON, J. M. et al. The VISA-A questionnaire: a valid and reliable index of the clinical severity of Achilles tendinopathy. **British Journal of Sports Medicine**, v. 35,

n. 5, p. 335, 2001.

SCATTONE SILVA, R. et al. Lower limb strength and flexibility in athletes with and without patellar tendinopathy. **Physical Theraoy in Sport**, v. 20, p. 19–25, 2016.

SCHULZ, K. F.; ALTMAN, D. G.; MOHER, D. CONSORT 2010 Statement: updated

guidelines for reporting parallel group randomised trials. **BMC Medicine**, v. 8, p. 18, 24 mar. 2010.

SILBERNAGEL, K.; BRORSSON, A.; LUNDBERG, M. The majority of patients with Achilles tendinopathy recover fully when treated with exercise alone: A 5-year follow- up. **American Journal of Sports Medicine**, v. 39, n. 3, p. 607–613, 17 mar. 2011.

SILBERNAGEL, K. G. et al. Eccentric overload training for patients with chronic Achilles tendon pain-a randomised controlled study with reliability testing of the evaluation methods. **Scand J Med Sci Sports**, v. 11, p. 197–206, 2001.

SILBERNAGEL, K. G. et al. Evaluation of lower leg function in patients with Achilles tendinopathy. **Knee Surgery, Sports Traumatology, Arthroscopy**, v. 14, n. 11, p. 1207–1217, 21 nov. 2006.

SILBERNAGEL, K. G. et al. Continued sports activity, using a pain-monitoring model, during rehabilitation in patients with Achilles tendinopathy: a randomized controlled study. **The American Journal of Sports Medicine**, v. 35, n. 6, p. 897–907, 1 jun. 2007.

SILBERNAGEL, K. G.; CROSSLEY, K. M. A proposed return-to-sport program for patients with midportion achilles tendinopathy: Rationale and implementation. **Journal of Orthopaedic and Sports Physical Therapy**, v. 45, n. 11, p. 876–886, 1 nov. 2015.

SIQUEIRA, F. B.; TEIXEIRA-SAMELA, L. F.; MAGALÃES, L. DE C. Análise das

propriedades psicométricas da versão brasiliera da escala TAMPA de cinesiofobia Siqueira et al, 2007. **Acta Ortop Bras**, v. 15, n. 1, p. 19–24, 2007.

SLEESWIJK VISSER, T. S. O. et al. Impact of chronic Achilles tendinopathy on health-related quality of life, work performance, healthcare utilisation and costs. **BMJ Open Sport and Exercise Medicine**, v. 7, n. 1, 26 mar. 2021.

STENSRUD, S.; ROOS, E. M.; RISBERG, M. A. A 12-week exercise therapy program in middle-aged patients with degenerative meniscus tears: A case series with 1-year follow-up. **Journal of Orthopaedic and Sports Physical Therapy**, v. 42,

n. 11, p. 919–931, 2012.

STEVENS, M.; TAN, C. W. Effectiveness of the alfredson protocol compared with a lower repetition-volume protocol for midportion achilles tendinopathy: A randomized controlled trial. **Journal of Orthopaedic and Sports Physical Therapy**, v. 44, n. 2,

p. 59–67, 1 fev. 2014.

SUZUKI, T.; CHINO, K.; FUKASHIRO, S. Gastrocnemius and soleus are selectively activated when adding knee extensor activity to plantar flexion. **Human Movement Science**, v. 36, p. 35–45, 2014.

VAN DER PLAS, A. et al. A 5-year follow-up study of Alfredson’s heel-drop exercise programme in chronic midportion Achilles tendinopathy. **British Journal of Sports Medicine**, v. 46, n. 3, p. 214, 3 mar. 2012.

VAN DER VLIST, A. C. et al. Which treatment is most effective for patients with Achilles tendinopathy? A living systematic review with network meta-analysis of 29 randomised controlled trials. **British Journal of Sports Medicine**, v. 55, n. 5, p. 249, 1 mar. 2021.

VLAEYEN, J. W. S. et al. **Fear of movement/( re) injury in chronic low back pain and its relation to behavioral performancePain**. [s.l: s.n.].

WARE, J. E.; KOSINSKI, M.; KELLER, S. D. **A 12-Item Short-Form Health Survey: Construction of Scales and Preliminary Tests of Reliability and Validity**. Disponível em: <https://oce-ovid.ez18.periodicos.capes.gov.br/article/00005650- 199603000-00003/HTML>. Acesso em: 3 jul. 2022.

**ANEXOS**

ANEXO 1: *International Physical Activity Questionnaire (IPAQ-SF)* / versão traduzida e validada em português do Brasil.


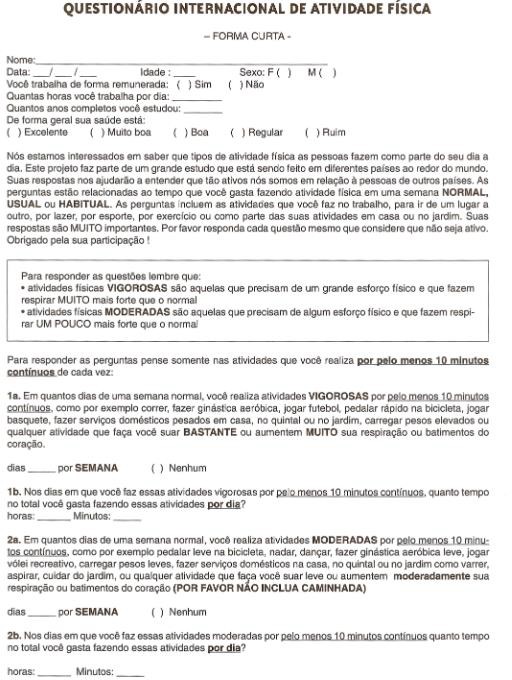


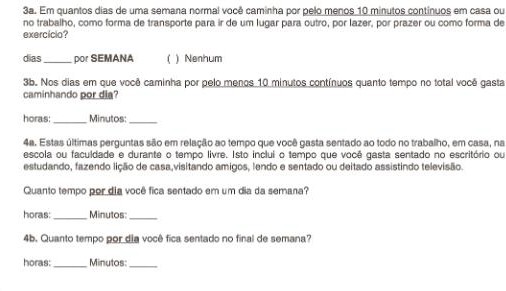


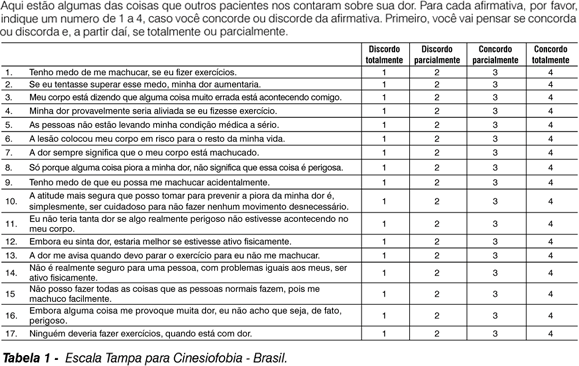
ANEXO 2: *Tampa Scale of Kinesiophobia (TSK)* / versão traduzida e validada em português do Brasil.

ANEXO 3: *Victorian Institute of Sport Assessment-Achilles* (VISA-A) / versão traduzida e validada em português do Brasil.


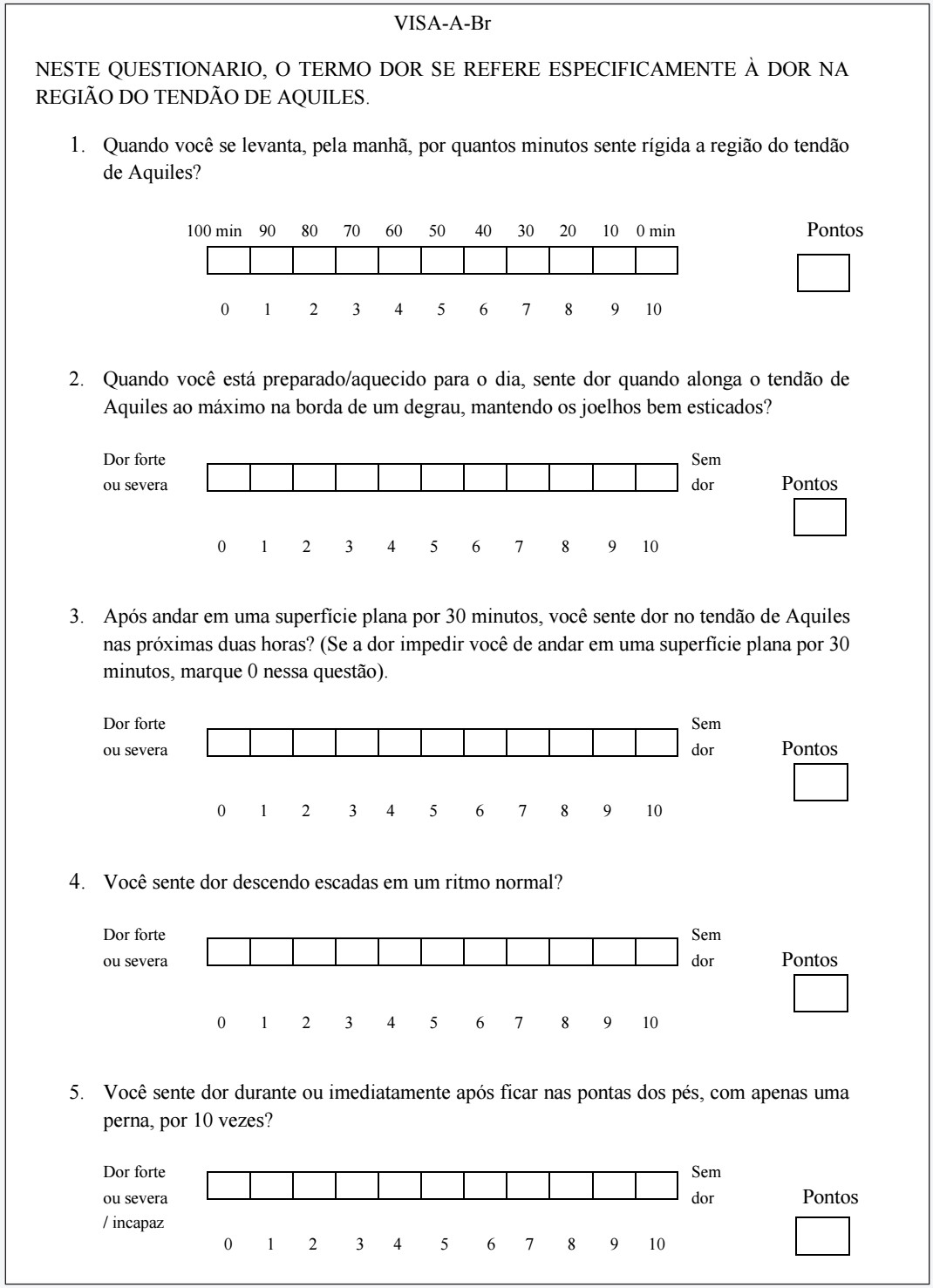


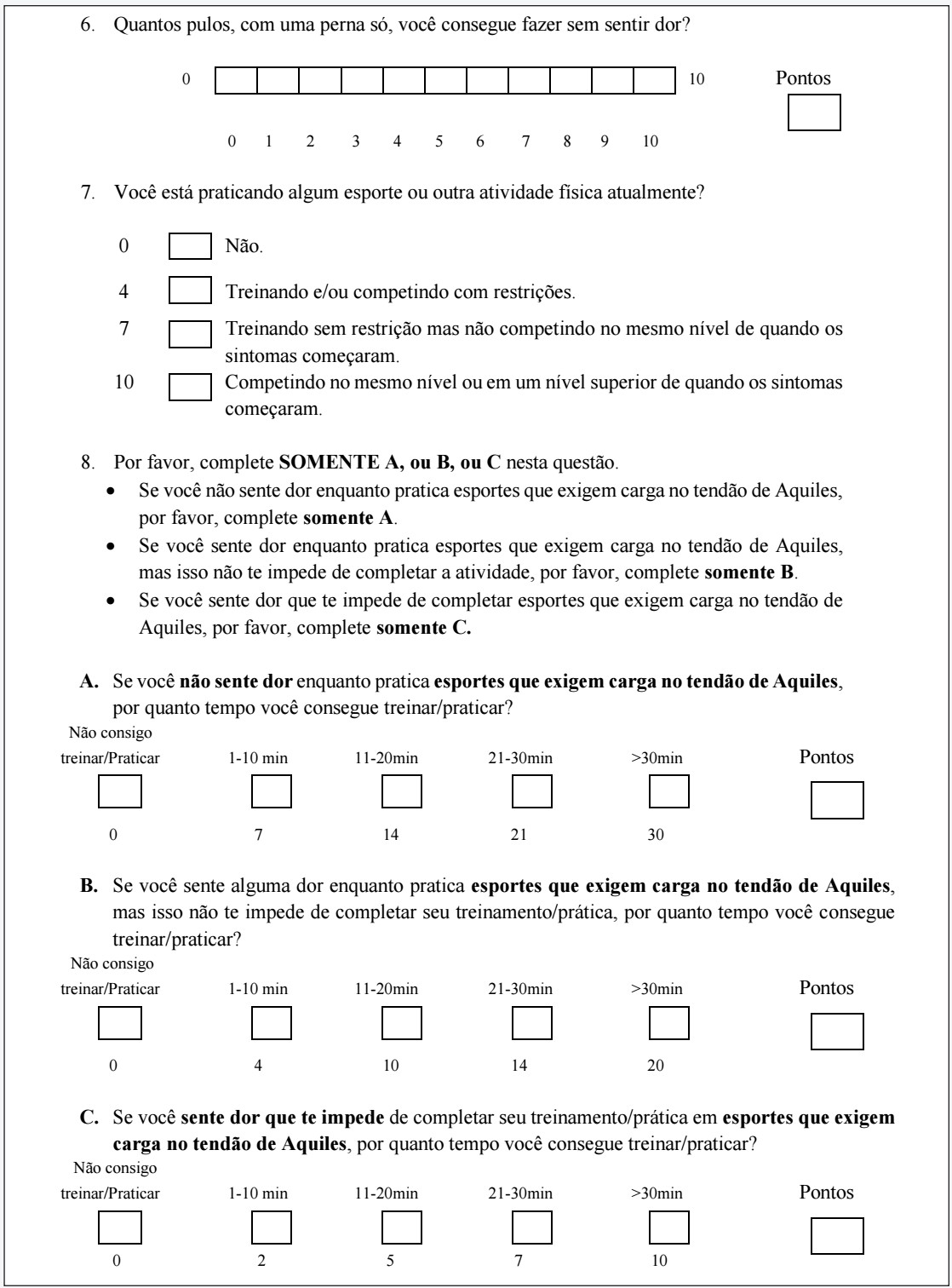


ANEXO 4: *Short-Form Health Survey (SF - 12) -* versão traduzida e validada em português do Brasil.


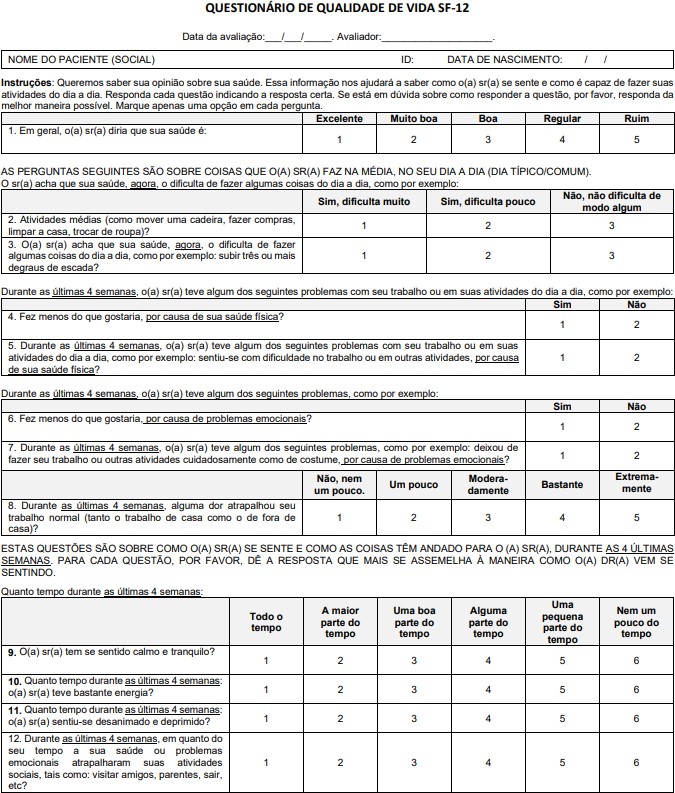


ANEXO 5: *Global Rating of Change* (GROC). Escala Global de Mudança Percebida

Nome: Data: / /

Com relação aos seus sintomas, você se sente:

+ 7: Extremamente melhor ( )

+ 6: ( )

+ 5: Muito melhor ( )

+ 4: ( )

+ 3: Melhor ( )

+2 : ( )

+ 1: Um pouco melhor ( ) 0: Sem mudanças ( )

- 1: Um pouco pior ( )

-2: ( )

- 3: Pior ( )

- 4: ( )

- 5: Muito pior ( )

- 6: ( )

- 7: Extremamente pior ( )

FEDERAL UNIVERSITY OF RIO GRANDE DO NORTE

POSTGRADUATE PROGRAM IN PHYSICAL THERAPY

## Comparison between moderate load resistance exercises and high load resistance exercises in the rehabilitation of runners with Achilles tendinopathy: Randomized and blind controlled trial

Author: Magno J. Moreno

Natal/RN 2023

Magno J Moreno

## Comparison between moderate load resistance exercises and high load resistance exercises in the rehabilitation of runners with Achilles tendinopathy: Randomized and blind controlled trial

Dissertation proposal as a requirement for obtaining

the title of doctor in physiotherapy in the sub-area of

​​assessment and intervention in the musculoskeletal

system

**Advisor:** Prof. Dr. Rodrigo Scattone Silva

Natal/RN 2023

LIST OF FIGURES

[**Figure 1**: Test position for assessing hip extension torque (a), knee extensor torque (b) and ankle plantar flexor torque (c)](#_bookmark10) [. …..1](#_bookmark10)4

[**Figure 2:** Single-leg heel rise test …..1](#_bookmark11)5

[**Figure 3:** Single-leg maximum drop jump test. Measurement of the distance from the ground to the fingertips of the participant’s raised upper limb (a); jump initiated from a 20 cm box (b); preparation for the vertical jump using only one foot (c); maximum vertical jump using only one foot (d) …..1](#_bookmark12)6

[**Figure 4:** Flowchart of SPIRIT – VISA-A, Victorian Institute of Sport Assessment-](#_bookmark13) [Achilles; VAS, Visual analogue scale; TSK, Tampa Scale of Kinesiophobia; IPAQ-](#_bookmark13) [SF, International Physical Activity Questionnaire; SF-36, Short-Form Health Survey;](#_bookmark13) [GROC, Global Rating of Change](#_bookmark13) ......17

[**Figure 5:** Initial position of the seated bilateral heel-raise exercise starting from maximum ankle dorsiflexion (a); Final position of the seated bilateral heel-raise exercise ending in maximum ankle plantar flexion (b) …..](#_bookmark18)19

[**Figure 6:** Standing bipodal heel raise: initial position in maximum dorsiflexion (a); final position in maximum plantar flexion (b) …..2](#_bookmark19)0

[**Figure 7:** Initial position of the knee extension exercise on the leg extension machine (a); final position of the knee extension exercise on the leg extension machine (b).. …..2](#_bookmark20)0

[**Figure 8:** Initial and final positions for the clam exercise with 60° of hip flexion (a); intermediate position for the clamshell exercise with 60° of hip flexion (b) …..2](#_bookmark21)1

[**Figure 9:** Seated single-leg heel raise: initial position in maximum ankle dorsiflexion (a) and final position in maximum ankle plantar flexion (b). Single-leg heel raise: initial position in maximum ankle dorsiflexion (c) and final position in maximum ankle plantar flexion (d)](#_bookmark23)………………………………………………………………………………………...21

[**Figure 10:** Initial position for the knee extension exercise on the leg extension machine (a). Final position for the knee extension exercise on the leg extension machine (b) …..2](#_bookmark24)2

[**Figure 11:** Single-leg deadlift exercise: Starting position(a), finishing position (b)……...2](#_bookmark25)2

[**Figure 12:** Fast two-leg jumps. …..2](#_bookmark27)3

[**Figure 13:** Fast alternating one-leg hops. …..2](#_bookmark28)4

[**Figure 14:** Wall running drill exercise: initial standing position (a); position to begin alternating support feet …. 2](#_bookmark31)5

LIST OF TABLES

[**TABLE** 1: Triceps surae exercise protocols for both groups……………………………..18](#_bookmark16)

LIST OF ABBREVIATIONS AND ACRONYMS

ACSM American College of Sports Medicine

CONSORT *Consolidated Standards of Reporting Trials*

VAS Visual Analogue Scale

HIG High-Intensity Group

MIG Moderate-Intensity Group

GROC Global Rating of Change

CI Confidence interval

IPAQ-SF International Physical Activity Questionnaire-Short Form

TSK Tampa Scale of Kinesiophobia

MCID Minimal Clinically Important Difference

ReBEC Brazilian Registry of Clinical Trials

RM Repetition Maximum

SEM Standard Error of the Mean

*SF-12 Short-Form Health Survey*

SPIRIT *Standard Protocol Items: Recommendations for Interventional Trials*

SPSS Statistical Package for the Social Science

UFRN Federal University of Rio Grande do Norte

VISA-A Victorian Institute of Sport Assessment-Achilles

# Summary

[ABSTRACT 8](#_Toc199341086)

[INTRODUCTION 9](#_Toc199341087)

[METHODS 11](#_Toc199341088)

[Study Design 11](#_Toc199341089)

[Eligibility Criteria 11](#_Toc199341090)

[Sample Size Calculation 12](#_Toc199341091)

[Randomization and Bias Minimization Strategies 12](#_Toc199341092)

[Assessments 13](#_Toc199341093)

[Interventions 17](#_Toc199341096)

[PHASE 1 (weeks 1–4) 19](#_Toc199341097)

[PHASE 2 (weeks 5–8) 21](#_Toc199341102)

[PHASE 3 (weeks 9–12) 23](#_Toc199341105)

[REFERENCES 2](#_Toc199341116)8

Supplementary Materials  [3](#_Toc199341119)5

# ABSTRACT

**Introduction:** Achilles tendinopathy has a high incidence in the general population and an even higher incidence in active individuals. Among runners, it is one of the most frequent health conditions, causing a great impact on the quality of life of these individuals. Despite its importance, the current treatment options lead to insufficient results in 5-10 year follow-ups. **Objective:** The primary objective of the study will be to investigate the short-term (6 and 12 weeks) and long-term (6 months) effects of a moderate load resistance exercise intervention [55% of 1 Repetition Maximum (55%1RM)] in comparison to high-load resistance exercise (90% of 1RM) in runners with Achilles tendinopathy in terms of pain and symptom severity. **Methods**: Sixty amateur runners will be randomly allocated into two groups: Group of high intensity exercises (GHI) and group of moderate intensity exercises (GMI). The GHI will start the treatment with triceps surae exercises with 55%1RM in the first and second week, 65%1RM in the third and fourth week, 75%1RM in the fifth and sixth week, 85%1RM in the seventh and eighth week and, from the ninth week until the end of the 12 weeks of treatment, you will perform the exercises with 90%1RM. The GMI will perform exercises with 55%1RM during the 12 weeks of intervention. The RM load will be reassessed every 2 weeks and the total training volume will be identical in both groups. Both groups will do strengthening exercises for quadriceps and gluteal muscles and will continue to perform physical/sports activities using the pain monitoring model strategy. Pain (VAS) and symptom severity (VISA-A), maximum isometric strength of ankle, knee, and hip muscles (handheld dynamometer), function of plantar flexors (functional tests), quality of life (SF-12) and perception of improvement (GROC). Primary outcomes (pain and symptom severity) will be evaluated before the intervention (baseline), at week 6, at week 12 (end of intervention) and 6 months after the intervention. The other outcomes will be evaluated at baseline and at the end of the intervention. Results will be analyzed using the intention-to-treat principle and will be compared by mixed-model analysis of variance (2-way ANOVA) with repeated measures (group x time) and Bonferroni post hoc.

**Keywords:** Achilles tendon; tendinitis; load; physiotherapy, biomechanics

# INTRODUCTION

Mid-portion Achilles tendinopathy presents an incidence of 2 to 3 cases per 1,000 individuals in the general population and is even more frequent among active individuals (DE JONGE et al., 2011). In a cohort study of 3,379 participants composed of recreational runners, the incidence of Achilles tendinopathy was 4.2%, of which 64% were classified as mid-portion tendinopathy (CHEN et al., 2023).

In addition to being considered the most prevalent health condition among runners, its lifetime cumulative incidence among former long-distance runners is 52% (KUJALA; SARNA; KAPRIO, 2005). The presence of pain (SLEESWIJK VISSER et al., 2021), disability and symptom severity (RABUSIN et al., 2021), impaired muscle function (HASANI et al., 2021; MCAULIFFE et al., 2019), and kinesiophobia (ALGHAMDI et al., 2021; CHIMENTI et al., 2021) are frequently observed in these patients, which may result in withdrawal from sports activities and impact quality of life (DE VOS et al., 2021; MALLIARAS, 2022; SLEESWIJK VISSER et al., 2021; VAN DER VLIST et al., 2021).

Currently, interventions involving progressive load exercises aiming at positive tendon adaptation are considered the first-line treatment for Achilles tendinopathy (DE VOS et al., 2021; ESCRICHE-ESCUDER; CASANÃ; CUESTA-VARGAS, 2020; VAN DER VLIST et al., 2021). Several variations of such interventions have been investigated over the past three decades in different types of lower limb tendinopathies, yielding positive outcomes (ESCRICHE-ESCUDER; CASANÃ; CUESTA-VARGAS, 2020). Notable interventions include eccentric exercises (ALFREDSON; PIETILÄ; LORENTZON, 1998), isometric exercises (O’NEILL et al., 2019), combined concentric-eccentric exercises (SILBERNAGEL et al., 2001), and heavy slow resistance training (HSRT) (BEYER et al., 2015; KONGSGAARD et al., 2009).

Despite the recognition of progressive exercise interventions as the gold standard for Achilles tendinopathy treatment, not all patients achieve full recovery, and studies have shown that 20–40% of patients report unsatisfactory outcomes after 10 years (JOHANNSEN; JENSEN; WETKE, 2018; LAGAS et al., 2022), and only 39% remain asymptomatic after 5 years (VAN DER PLAS et al., 2012). Therefore, alternative approaches aimed at promoting long-term rehabilitation outcomes are warranted.

A number of studies have evaluated variations in strength training parameters (AGERGAARD et al., 2021; BREDA et al., 2021; STEVENS; TAN, 2014), such as repetitions, sets, frequency, and load, in the pursuit of an optimal exercise dose and loading magnitude for patients with tendinopathy. Recently, Arampatzis et al. (2020) suggested that, when comparing programs with equal training volume but differing loads, only higher-load protocols promote tendon adaptations, including increased stiffness and cross-sectional area, in individuals with healthy Achilles tendons.

It is known that in individuals with healthy Achilles tendons, moderate load exercises [55% of 1 repetition maximum (1RM)] improve plantar flexion strength and induce hypertrophy in the triceps surae, but do not elicit adaptive changes in the tendon (ARAMPATZIS; KARAMANIDIS; ALBRACHT, 2007; ARAMPATZIS; MERSMANN; BOHM, 2020; BOHM et al., 2014). This is believed to occur because contractions at this intensity do not produce tendon strain within the range considered optimal (between 4.5% and 6%) to induce positive mechanical and morphological adaptations. Conversely, it has been demonstrated that high-load exercises (90% of 1RM) not only improve strength and induce hypertrophy, but also provide an effective mechanical stimulus to the Achilles tendon, promoting beneficial tissue adaptations and improved mechanical properties in healthy tendons (ARAMPATZIS; KARAMANIDIS; ALBRACHT, 2007; ARAMPATZIS; MERSMANN; BOHM, 2020; BOHM et al., 2014; LAZARCZUK et al., 2022). However, the effects of different loading magnitudes in individuals with Achilles tendinopathy remain unknown.

The primary objective of this study is to assess the short-term (6 and 12 weeks) and long-term (6 months) effects of moderate-load resistance exercises (55% 1RM) compared to high-load resistance exercises (90% 1RM) on pain and symptom severity in amateur runners with Achilles tendinopathy. The secondary objectives are to evaluate the effects of these interventions on muscle function and quality of life in this population.

# METHODS

## Study Design

This is a protocol for a randomized and blinded controlled trial with parallel groups (1:1 ratio), comparing two exercise programs in runners diagnosed with Achilles tendinopathy. The study will be conducted at the Department of Physical Therapy at the Federal University of Rio Grande do Norte (UFRN), following the Standard Protocol Items: Recommendations for Interventional Trials (SPIRIT) guidelines (CHAN et al., 2013). Participants will be randomly allocated into two groups: the High-Intensity Group (HIG) and the Moderate-Intensity Group (MIG). This clinical trial will be reported in accordance with the Consolidated Standards of Reporting Trials (CONSORT) guidelines (SCHULZ; ALTMAN; MOHER, 2010), and the protocol will be registered in the *Registro Brasileiro de Ensaions Clínicos* (ReBEC) platform.

## Eligibility Criteria

Participants will include male amateur runners aged 18 to 60 years with a clinical diagnosis of mid-portion Achilles tendinopathy. Diagnosis will be based on the following criteria: localized pain in the mid-portion of the Achilles Tendon (2 to 6 cm above the Achilles tendon insertion on the calcaneus), pain during running, tendon thickening, morning stiffness, and tenderness to palpation (DE VOS et al., 2021; HABETS et al., 2021).

Participants will be excluded if presenting: (1) exclusively insertional Achilles tendinopathy, (2) washout period from other treatments inferior to 4 weeks, (3) corticosteroid injection in the Achilles tendon region or use of fluoroquinolone antibiotics within the past 12 months (LEWIS; COOK, 2014), (4) other injuries to the affected lower limb in the last 3 months, (5) musculoskeletal surgery involving the spine or lower limbs in the past 12 months, (6) history of Achilles tendon rupture or (7) systemic conditions that may interfere with rehabilitation (e.g., rheumatoid arthritis or diabetes) (HABETS et al., 2021).

Participants will be recruited through active searches in running group registries in Natal, Rio Grande do Norte (Brazil). Advertisement will be made online (Instagram, WhatsApp, Telegram, Facebook) and in common running locations using banners. Runners reporting Achilles tendon pain will be invited for a clinical eligibility screening.

To characterize the sample, physical activity level of each participant will be assessed using the short version of the International Physical Activity Questionnaire-Short Form (IPAQ-SF). It is the Brazilian Portuguese version (PINTO et al, 2005) of a questionnaire which contains eight items estimating time spent in walking, moderate and high intensity activities, and sedentary, sitting behavior over a week (**Supplementary Material 1**). The product between the duration (minutes/day) and the frequency (days/week) of the activities reported by the participants will be performed (MATSUDO et al., 2001).

Furthermore, kinesiophobia will be assessed using the Brazilian version of the Tampa Scale of Kinesiophobia (TSK) (Siqueira; Teixeira-Samela; Magalhães, 2007). It consists of 17 items assessing fear of movement and symptom severity (**Supplementary Material2**). Total scores range from 17 to 68, with higher scores indicating greater kinesiophobia (SIQUEIRA; TEIXEIRA-SAMELA; MAGALHÃES, 2007; VLAEYEN et al., 1995).

## Sample Size Calculation

Sample size was calculated considering a 95% confidence level (α), 80% power (β), and a minimal clinically important difference (MCID) of 14 points in the Victorian Institute of Sport Assessment – Achilles (VISA-A) questionnaire (LAGAS et al., 2021). The standard deviation was based on a previous clinical trial that used the same primary outcome (HABETS et al., 2021). This calculation indicated a sample size of 54 participants, 27 per group. Accounting for a potential 10% dropout, the final sample size was increased to 60 participants (30 per group).

## Randomization and Bias Minimization Strategies

Participant randomization will be performed using a computer-generated block randomization (block size of four) from the website [www.randomization.com](http://www.randomization.com). The randomization list will be generated by an independent researcher who will not be involved in assessments, interventions, or data analysis. From this list, sequentially numbered, sealed, opaque envelopes will be prepared to ensure allocation concealment.

Interventions will take place at the Federal University of Rio Grande do Norte (UFRN) Department of Physical Therapy and/or local gyms and will be supervised by experienced physical therapists. The principal investigator will remain blinded to group allocation and will perform all study assessments. To ensure blinding, the investigator will not participate in intervention, and participants will be instructed not to disclose their assigned intervention group to either the assessor or other participants.

##

## Assessments

### Primary Outcomes

The primary outcomes will be pain and symptom severity. These will be assessed at baseline (pre-intervention), week 6 (midpoint), week 12 (end of intervention), and 6 months post-intervention.

Symptom severity and disability will be measured using the Brazilian Portuguese version of the Victorian Institute of Sport Assessment – Achilles questionnaire (VISA-A) (DE MESQUITA et al., 2018) (**Supplementary Material3**). VISA-A is a valid, reliable and easy-to-apply tool for measuring outcomes in Achilles tendon intervention studies (MARTIN et al., 2018; ROBINSON et al., 2001). The questionnaire consists of eight items assessing pain and function during daily and sports activities. Scores range from 0 to 100, with higher scores indicating less pain and disability. Improvements of more than 14 points are considered clinically significant (LAGAS et al., 2021).

Pain will be assessed using the Visual Analogue Scale (VAS), where 0 represents no pain and 10 indicates the worst imaginable pain (PRICE et al., 1983). Participants will report the worst pain experienced in the past week (HABETS et al., 2021), as well as pain during the single-leg heel raise test and single-leg vertical jump test (SILBERNAGEL et al., 2011; HANLON et al., 2021). A reduction of ≥3 points on the VAS will be considered clinically significant (CHIMENTI et al., 2021).

### Secondary Outcomes

Secondary outcomes will include maximum isometric strength of hip and knee extensors and ankle plantar flexors, plantar flexor muscle function, quality of life, and the participants’ perception of clinical improvement or worsening. These outcomes will be assessed at baseline and immediately at the end of the 12-week intervention.

To measure isometric strength of hip extension, knee extension, and ankle plantar flexion, a portable handheld dynamometer (Lafayette Instruments, IN, USA) will be used. Non-elastic straps will also be used to stabilize participants and secure the dynamometer, eliminating influence from the evaluator's force during testing (SCATTONE SILVA et al., 2016).

Hip extension strength will be assessed with the participant in prone position, hip in a neutral position in all three planes, and knee flexed at 90°. A stabilization strap will be positioned over the pelvis, securing it to the table. The dynamometer will be placed immediately proximal to the popliteal fossa of the tested limb and secured with a second non-elastic strap fixed to the table. Participants will be allowed to use their upper limbs to stabilize their trunk (SCATTONE SILVA et al., 2016). The participant will receive the verbal instruction: “Push your foot up towards the ceiling” (**FIGURE 1a**).

Knee extension strength will be assessed with the participant in supine position, knee flexed at 30°, supported by a non-deformable roll placed beneath the knee, at the popliteal fossa. A strap will be fixed around the malleoli to secure the dynamometer at the midpoint between them, on the anterior aspect of the ankle. Participants will cross their arms over their chest and be instructed to “Push forward as if trying to extend the knee” (**FIGURE 1b**) (SCATTONE SILVA et al., 2016).

Ankle plantar flexion strength will be assessed with the participant in prone position, with the tested limb in a neutral position. The dynamometer will be placed against the plantar surface at the metatarsal heads and secured with a strap around the table. The participant will receive the verbal instruction: “Push the tip of your foot down as hard as you can” (**FIGURE 1c**) (SCATTONE SILVA et al., 2016).

All isometric strength tests will consist of four repetitions. The first repetition will be for familiarization with the test, and the subsequent three will be used for data analysis. During each repetition, the participant will be instructed to hold maximum force for 5 seconds, with a 15-second rest between repetitions (SCATTONE SILVA et al., 2016).


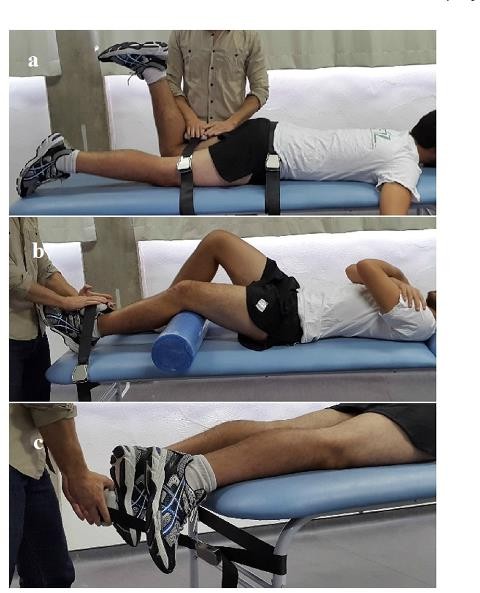


**Figure 1**: Test position for the assessment of hip extension torque (a), knee extensor torque (b), and ankle plantar flexor torque (c).

To assess the function of the plantar flexor muscles, two tests will be used, the single-leg heel rise test and the single-leg vertical drop jump test (SILBERNAGEL et al., 2006). The single-leg heel rise test evaluates the number of repetitions a participant can perform while standing on one leg (**FIGURE** **2**). The participant will stand on a ramp with a 10° incline, with a 24 mm marker positioned below the lateral malleolus. They will be instructed to use only two fingers against the wall at shoulder height for balance. A metronome will guide the pace at 30 heel raises per minute. The Calf Raise app will be used to estimate the total work performed during the activity (FERNANDEZ et al., 2023). Participants will be asked to lift their heel as high as possible and then return to the starting position, keeping knees extended and trunk upright. The test ends when the participant cannot continue, fails to keep the pace, or cannot complete a full heel raise (SILBERNAGEL et al., 2006).


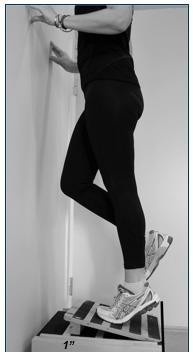


**Figure 2:** Single-leg heel rise test

The single-leg vertical drop jump test (**FIGURE 3**) begins by measuring the height from the floor to the fingertips, with the arm raised overhead (**FIGURE 3A**). The participant will hold a sticky note (post-it) and place it on the wall at the peak of their jump. To perform the test, the participant will stand on one leg on a 20 cm box, let themselves drop down, and then immediately perform a maximal vertical jump (**FIGURES 3B–3D**). The sticky note marks the highest point reached during the jump (SILBERNAGEL et al., 2011).


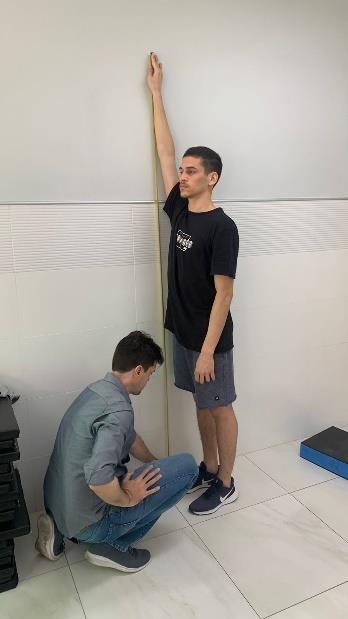

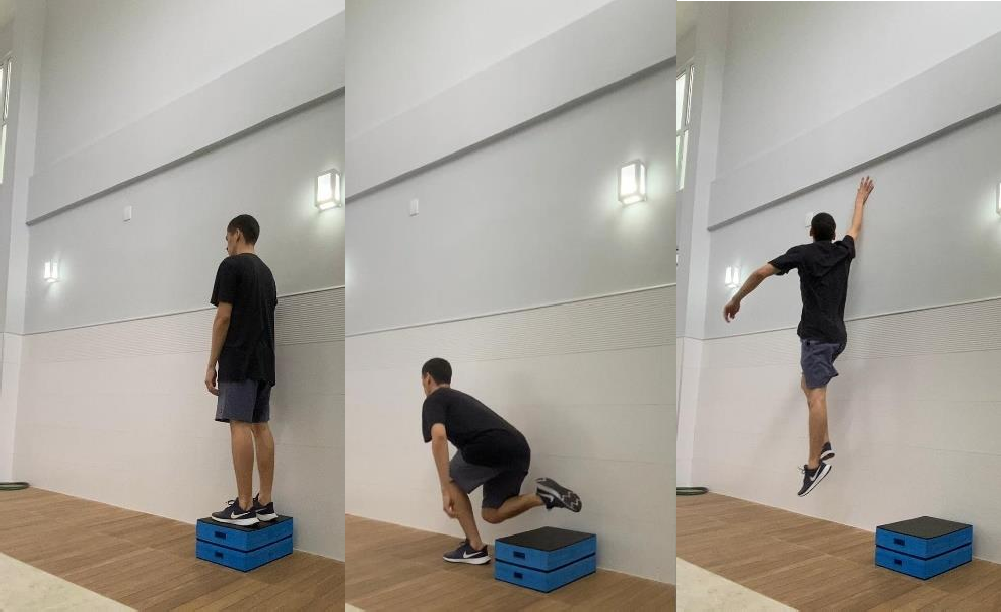


**a**

**b**

**c**

**d**

**Figure 3:** Single-leg maximum drop jump test. Measurement of the distance from the ground to the fingertips of the participant’s raised upper limb (a); jump initiated from a 20 cm box (b); preparation for the vertical jump using only one foot (c); maximum vertical jump using only one foot (d).

To evaluate the **impact of Achilles tendinopathy on quality of life**, the validated Brazilian Portuguese version of the **Short-Form Health Survey (SF-12)** will be used (CAMALIER, 2004), which consists of 12 items that represent the 8 domains of the Form Health Survey and generates a summary score ranging from 0 to 100, where 0 represents the worst general health status and 100 the best (WARE; KOSINSKI; KELLER, 1996).

The Global Rating of Change (GROC) will be used to assess participants’ perceived improvement or worsening after the intervention (JAESCHKE; SINGER; GUYATT, 1989). This is a 15-point Likert scale ranging from -7 (a very great deal worse) to +7 (a very great deal better), with 0 indicating no change (**Supplementary Material5**). Changes of 4 points or more are considered clinically important in patients with Achilles tendon pain (MCCORMACK et al., 2015). The SPIRIT flowchart (**FIGURE 4**) illustrates the sequence of activities that will be performed during the research.

|  | **STUDY PERIOD** | | | | | | |
| --- | --- | --- | --- | --- | --- | --- | --- |
|  | **Recruitment** | **Allocation** | **Post-allocation** | | | | **Follow up** |
| **PERIOD (WEEKS)** | **-1** | **0** | **w1/w5** | **w6** | **w7/w11** | **w12** | **w26** |
| **Recruitment** | X |  |  |  |  |  |  |
| **Screening and eligibility** | X |  |  |  |  |  |  |
| **ICF** |  | X |  |  |  |  |  |
| **Listo of other procedures – baseline measures** |  | X |  |  |  |  |  |
| **Allocation** |  | X |  |  |  |  |  |
| **IPAQ-SF** |  | X |  |  |  |  |  |
| **TSK** |  | X |  |  |  | X |  |
| **INTERVENTION:** |  |  |  |  |  |  |  |
| **High Load Group (HLG)** |  |  | ♦ |  |  | ♦ |  |
| **Moderate Load Group (MLG)** |  |  | ♦ |  |  | ♦ |  |
| **EVALUATIONS:** |  |  |  |  |  |  |  |
| **Diagnosis of Achilles tendinopathy** | X |  |  |  |  |  |  |
| **Baseline Assessments** |  | X |  |  |  |  |  |
| **VISA-A** |  | X |  | X |  | X | X |
| **VAS** |  | X |  | X |  | X | X |
| **Maximum isometric strength** |  | X |  |  |  | X |  |
| **Function of the plantar flexors** |  | X |  |  |  | X |  |
| **SF-12** |  | X |  |  |  | X |  |
| **GROC** |  |  |  |  |  | X |  |

**Figure 4: Flowchart of SPIRIT** – **ICF**, informed consent form; **IPAQ-SF**, International Physical Activity Questionnaire; **TSK**, Tampa Scale of Kinesiophobia; **VISA-A**, Victorian Institute of Sport Assessment-Achilles; **VAS**, Visual analogue scale; **SF-12**, Short-Form Health Survey; **GROC**, Global Rating of Change

## Interventions

The intervention period will last for 12 weeks, with sessions performed three times per week. At least one weekly session will be supervised individually by a trained physiotherapist with expertise in orthopedics, who will undergo specific training on the activities performed in the clinical trial. For the other two sessions, participants will receive detailed written instructions on exercise execution without supervision and pain monitoring procedures.

During the first three weeks, participants will be instructed not to engage in physical activities that overload the Achilles tendon (Habets et al., 2021). After this period, they will be allowed to resume sports activities guided by the pain-monitoring model proposed by Silbernagel et al. (2007). This model allows pain levels up to 5 on a 0–10 scale during daily, occupational, or athletic activities, provided that symptoms decrease within 24 hours after activity (SILBERNAGEL; CROSSLEY, 2015). Participants will be advised to avoid activities that cause pain above this 5/10 threshold (HABETS et al., 2021; SILBERNAGEL et al., 2007; SILBERNAGEL; CROSSLEY, 2015).

Participants will be allocated into two groups with an equivalent total exercise volume targeting the triceps surae and Achilles tendon: the high intensity group (HIG), which will begin triceps surae exercises at 55% of 1RM in weeks 1–2, progressing to 65% in weeks 3-4, 75% in weeks 5-6, 85% in weeks 7–8, and 90% in weeks 9–12; and the moderate intensity group (MIG), which will perform all triceps surae exercises at a constant 55% of 1RM throughout the 12-week period (**Table 1**):

**Table 1: Triceps surae exercise protocols for both groups.**

|  | Weeks  1-2 | Weeks  3-4 | Week  5 | Week  6 | Week  7 | Week  8 | Weeks  9-12 | Total number of repetitions |
| --- | --- | --- | --- | --- | --- | --- | --- | --- |
| HIG | | | | | | | | |
| % of 1RM | 55 | 65 | 75 | 75 | 85 | 85 | 90 | 1,054 |
| Repetitions | 15 | 15 | 10 | 8 | 8 | 6 | 4 |  |
| Sets | 3 | 3 | 3 | 4 | 4 | 4 | 5 |  |
| MIG | | | | | | | | |
| % of 1RM | 55 | 55 | 55 | 55 | 55 | 55 | 55 | 1,053 |
| Repetitions | 17 | 15 | 12 | 10 | 8 | 7 | 6 |  |
| Sets | 3 | 3 | 3 | 3 | 3 | 3 | 3 |  |

HIG, high-intensity group; MIG, moderate-intensity group; 1RM, one-repetition maximum.

In addition to triceps surae-specific exercises, both groups will perform strengthening exercises targeting the kinetic chain, synergistic muscles of the triceps surae in running, and/or muscles that are often weak in individuals with Achilles tendinopathy (HABETS et al., 2021). These exercises will follow the American College of Sports Medicine (ACSM) recommendations for resistance training in trained individuals (RATAMESS et al., 2009) and, to ensure proper progression, the “plus two rule” will be applied: in the final set, if the participant can complete at least two additional repetitions beyond the prescribed number, the load will be increased in the next session (STENSRUD; ROOS; RISBERG, 2012).

Before all resistance exercises, a warm-up set will be performed with no load. Every two weeks, a 10-RM submaximal test will be used to estimate the current 1RM and adequately adjust the training loads (BRZYCKI, 1993; REYNOLDS et al., 2006). In exercises using elastic bands, resistance will start two levels below the estimated 1RM and progress by increasing one resistance level (BALDON et al., 2014).

The exercise protocol for both groups will be divided into distinct phases, including slow progressive strengthening (3 seconds for the concentric phase and 3 seconds for the eccentric phase), plyometric exercises and running-specific strengthening. The phases are described below.

## PHASE 1 (weeks 1–4)

### Seated bipodal heel raise

This exercise is performed with the participant seated on the edge of a chair, avoiding contact with the backrest, with hips and knees flexed at 90° (**FIGURE 5**). The participant is instructed to raise their heels maximally while maintaining forefoot contacts, then slowly lower it into maximum dorsiflexion. Resistance is applied to the distal anterior thigh, just above the patella (BAXTER et al., 2021; CHIMENTI et al., 2023).


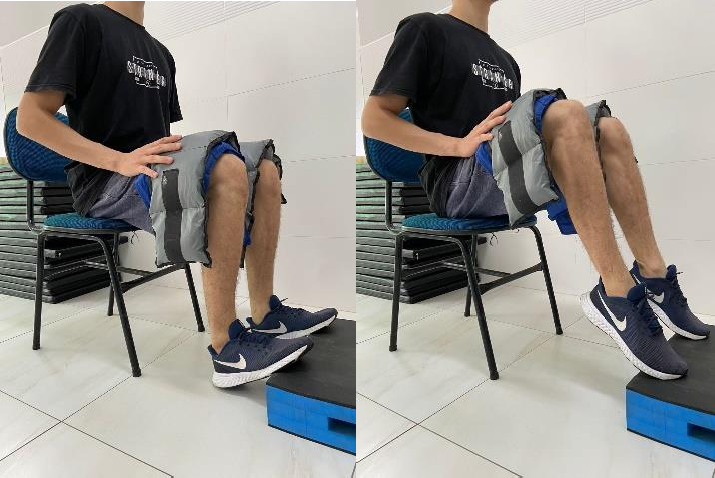


**a**

**b**

**Figure 5:** Initial position of the seated bilateral heel-raise exercise starting from maximum ankle dorsiflexion (a); Final position of the seated bilateral heel-raise exercise ending in maximum ankle plantar flexion (b).

### Standing bipodal heel raise

The participant stands on the balls of both feet at the edge of a step, using hand support on a wall for balance. Feet should be shoulder-width apart (**FIGURE 6**). The participant is instructed to elevate the heels from maximum dorsiflexion to maximum plantarflexion. Load is applied via a weighted vest worn at shoulder level (BAXTER et al., 2021; CHIMENTI et al., 2023).

**Figure 6:** Standing bipodal heel raise: initial position in maximum dorsiflexion (a); final position in maximum plantar flexion (b).


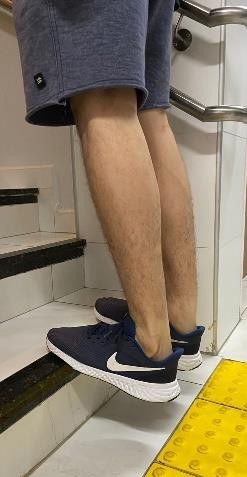

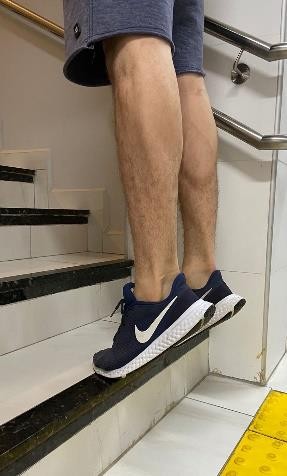


**a**

**b**

###

### Seated double-leg knee extension on machine

This exercise is performed on a standard knee extension machine. The participant sits with knees flexed at 90° and hips at 100° of flexion. The machine’s lever arm is positioned immediately above the ankles (**FIGURE 7**). The participant is instructed to fully extend the knees to 0° and return to 90° of flexion (JAKOBSEN et al., 2012).


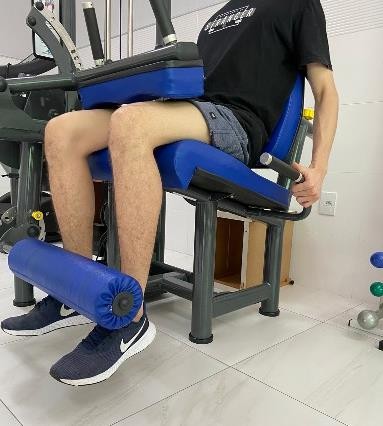

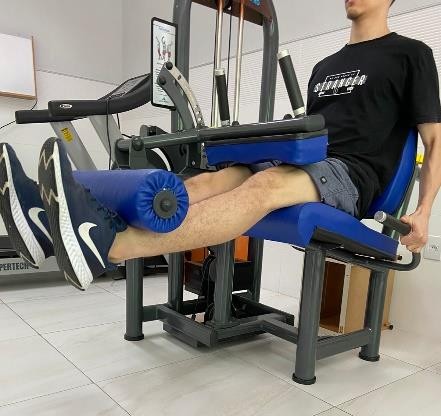


**a**

**b**

**Figure 7:** Initial position of the knee extension exercise on the leg extension machine (a); final position of the knee extension exercise on the leg extension machine (b).

### Clam exercise with 60º hip flexion

The participant lies in a side-lying position with the hip flexed to 60°, knees to 90°, and a resistance band placed above the knees (**FIGURE 8**). The task is to perform hip abduction and external rotation while maintaining heel contact and anterior superior iliac spines facing forward, and then to return to the starting position. (BISHOP et al., 2018; DISTEFANO et al., 2009).


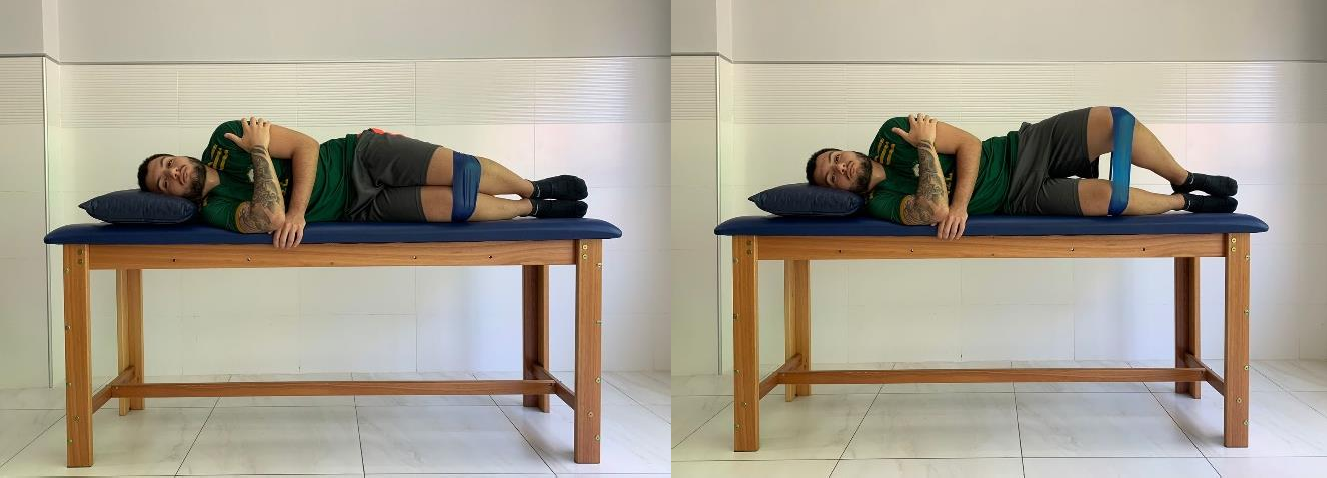


**a**

**b**

**Figure 8:** Initial and final positions for the clam exercise with 60° of hip flexion (a); intermediate position for the clamshell exercise with 60° of hip flexion (b).

## PHASE 2 (weeks 5–8)

In phase two, the exercises of seated unilateral heel raise and standing unilateral heel raise will be performed (**FIGURE 9**). The execution of these two exercises is identical to that of Phase 1, except that they are performed on one leg. (BAXTER et al., 2021; CHIMENTI et al., 2023).


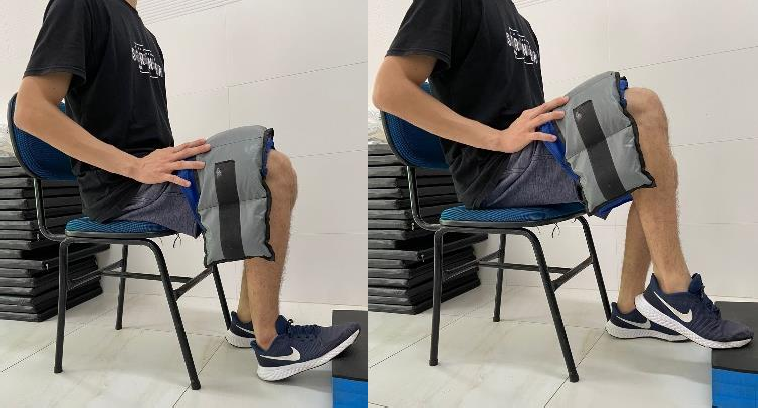

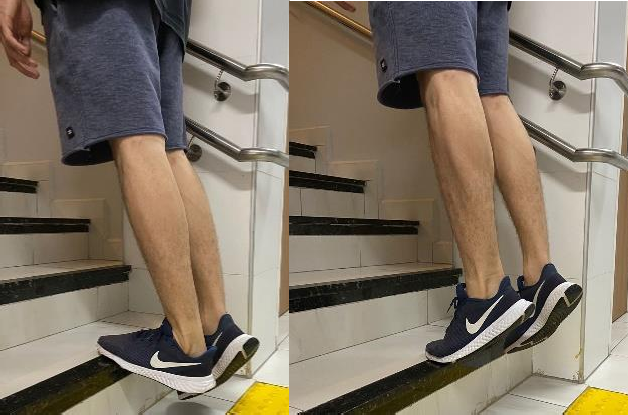


**a**

**b**

**c**

**d**

**Figure 9:** Seated single-leg heel raise: initial position in maximum ankle dorsiflexion (a) and final position in maximum ankle plantar flexion (b). Single-leg heel raise: initial position in maximum ankle dorsiflexion (c) and final position in maximum ankle plantar flexion (d).

###

### Unilateral knee extension on machine

This exercise is performed on a standard knee extension machine (**FIGURE 10**), while the participant is seated with the working knee at 90° and the hip at 100° flexion. The resistance arm is positioned immediately above the ankle. The participant is instructed to perform a full knee extension (0º) and return to the starting position (JAKOBSEN et al., 2012).


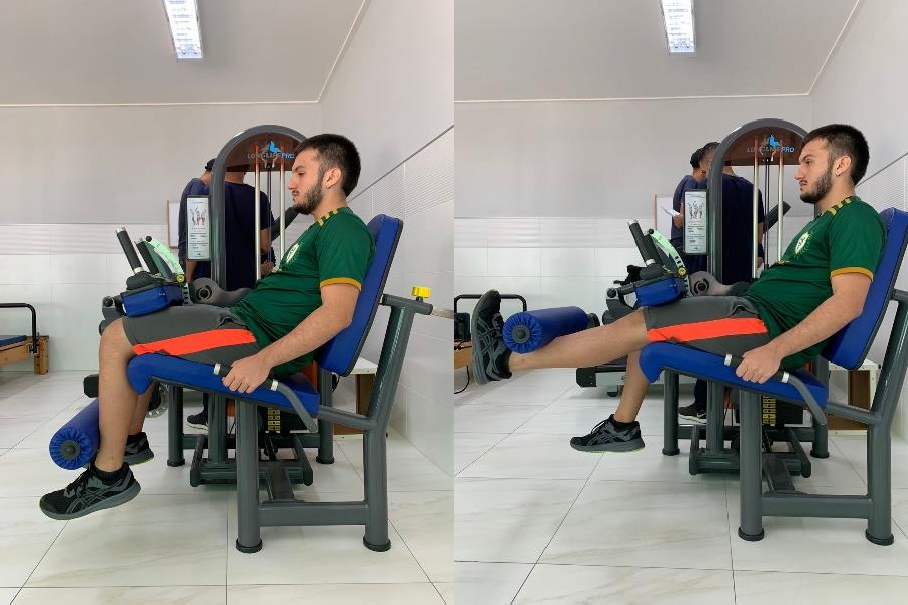


**a**

**b**

**Figure 10:** Initial position for the knee extension exercise on the leg extension machine (a). Final position for the knee extension exercise on the leg extension machine (b).

### Unilateral deadlift

In the unilateral deadlift (**FIGURE 11**), the participant stands on the affected leg, with the knee slightly flexed (30°) while holding dumbbells. The participant is instructed to flex the trunk and hip forward to about 90°, maintaining the 30° knee flexion, and then return to the starting position, all the while keeping the knee aligned over the toes (COLLINGS et al., 2023; DISTEFANO et al., 2009).


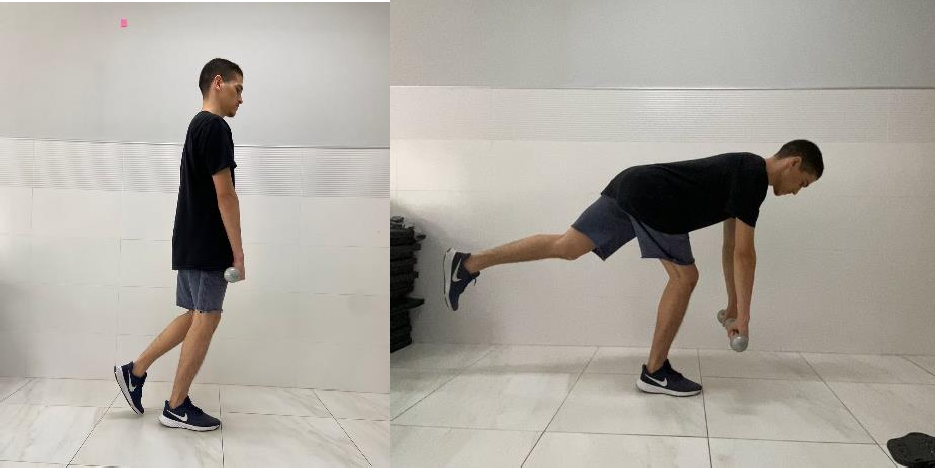


**a**

**b**

**Figure 11:** Single-leg deadlift exercise: initial position (a), final position (b).

##

## PHASE 3 (weeks 9–12)

In Phase 3, participants will perform plyometric exercises during the supervised weekly session. During the other two weekly sessions, they will perform the same exercises from Phase 2.

### Fast double-leg jumps

This exercise (**FIGURE 12**) starts with the participant standing upright, feet hip-width apart, and arms relaxed. The participant performs rapid, small jumps, lifting both feet off the ground simultaneously and landing without allowing the heels to touch the floor. No external resistance is used; bodyweight provides the load. The exercise consists of 3 sets of 20 seconds, with 5-second increments added each session if the participant completes the target duration (CHIMENTI et al., 2023).


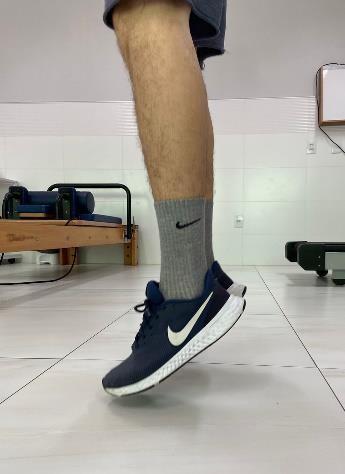


**Figure 12:** Fast double-leg jumps.

### Fast alternating one-leg hops

In this exercise (**FIGURE 13**), the participant stands with feet hip-width apart, and arms relaxed. The participant performs alternating hops, lifting both feet off the ground simultaneously and landing on one foot at a time. 3 sets of 20 seconds are performed, with 5-second increments added when the participant meets the target duration (CHIMENTI et al., 2023).


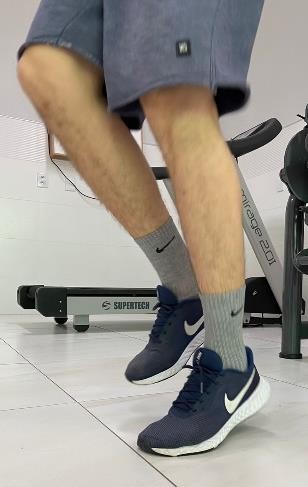


**Figure 13:** Fast alternating one-leg hops.

###

### Double-leg drop vertical jump

This exercise begins with the participant standing on a 20 cm box. The participant is instructed to drop down in from the box and then immediately perform a maximal vertical jump, landing at the same spot. The exercise consists of 5 sets of 20 repetitions, with 1-minute rest intervals between sets (ANDO et al., 2021; BAXTER et al., 2021).

###

### Drop vertical jump single-leg

This exercise begins with the participant standing on a 20 cm box. They are instructed to drop down in front of the box using only the affected leg and then immediately perform a single-leg vertical jump, landing on the same leg at the same spot. The exercise includes 5 sets of 20 jumps, with 1-minute rest between sets (ANDO et al., 2021; BAXTER et al., 2021).

### Wall running drill

In this exercise (**FIGURE 14**), the participant stands facing a wall, hands placed shoulder-width and shoulder-height apart, with elbows fully extended. They are instructed to take a step back so that the trunk and lower limbs form a 45° angle with the ground. One foot is raised to approximately 100° of hip and knee flexion, while the supporting ankle remains in plantarflexion, behind the hip. The participant is instructed to alternate foot contacts with quick jumps 3 times, keeping heels off the ground (LIEBENSON, 2009). One set consists of 10 repetitions per leg. This exercise was selected for its high soleus muscle recruitment during plantarflexion with the knee extended and for its biomechanical similarity to running (SUZUKI; CHINO; FUKASHIRO, 2014).


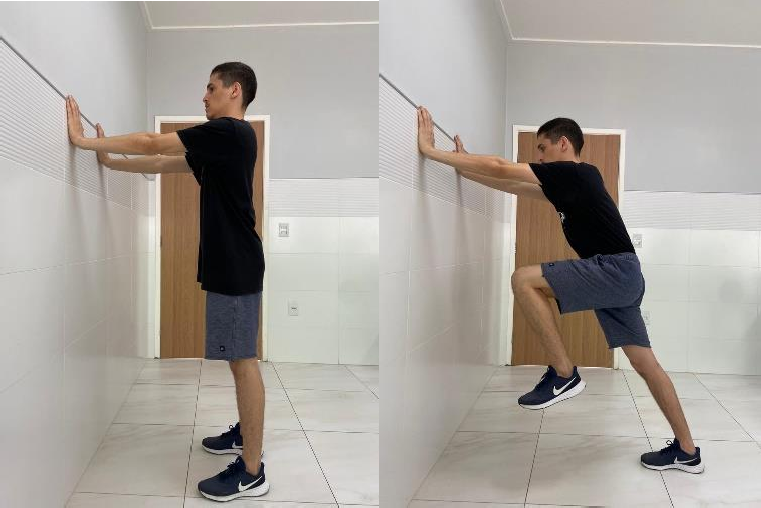


**a**

**b**

**Figure 14:** Wall running drill exercise: initial standing position (a); position to begin alternating support feet.

## Monitoring of Adverse Effects

In the event of any adverse effects, such as the onset of pain or swelling during or after the intervention, the participant will be instructed to contact one of the supervising physiotherapists, which will jointly determine whether the intervention should be adjusted, temporarily suspended, or permanently discontinued.

All participants will maintain a training diary to record their exercise sessions. These records will include information such as training intensity (repetitions, sets, and loads), session attendance, pain experienced during training (VAS), and any deviations from the prescribed intervention protocols (AGERGAARD et al., 2021).

Participants will be instructed not to undergo any other form of treatment during the study period. If any analgesic medication or pain-relief modality is used, participants must record the type, dosage, and parameters of use, and report this information during the post-intervention assessments (DIAS ARAÚJO et al., 2022).

## Risks and Benefits

During the study, participants may experience occasional muscle discomfort and potential risks such as falls during jumping or step exercises. The risk of falling will be mitigated through the use of support objects that participants can hold onto if they lose balance. Additionally, a researcher will remain in proximity during all evaluations and supervised sessions to assist the participant if necessary. There is also a risk of delayed-onset muscle soreness on the day following training sessions. These risks will be minimized through gradual load progression and supervision by a physiotherapist, who will guide participants in properly executing the exercises during both in-person sessions and based on the instructional materials provided.

As for the benefits, participants will receive free treatment for their condition, delivered by an experienced physiotherapist and will also be given access to data collected during the study, such as lower limb muscle strength and calf muscle endurance, which will be compiled into a comprehensive report. This report will be made available to participants at the end of the study, once data processing is complete, and will be written in easily understandable language. In the event of any complications or health damage related to the study, the principal investigator is responsible for ensuring the participant's right to complete and free medical assistance, covering any direct or indirect, immediate or delayed harm, for as long as necessary. Participants will also have the right to seek compensation for any injuries related to the research, and may request reimbursement for any expenses incurred from their participation.

## Data Analysis

Data will be analyzed by a researcher who will be blinded to group allocation. This researcher will be responsible solely for outcome assessments and data analysis during the trial. The software Statistical Package for the Social Sciences (version 17.0; SPSS Inc., Chicago, IL), will be used for statistical analyses. To verify the normality of data distributions and homoscedasticity, the Shapiro-Wilk test and Levene's test will be applied, respectively. Outcome measures will be analyzed using mixed-model analysis of variance (two-way ANOVA) with repeated measures (group × time). When appropriate, Bonferroni-adjusted post hoc tests will be applied. Results will be reported as mean ± standard error of the mean (SEM) along with 95% confidence intervals (CIs). Sphericity will be assessed using Mauchly’s test, and, if violated, the Greenhouse-Geisser correction will be employed.

All participants retain the right to withdraw from the study at any time. In such cases, their data will be included in the analysis using the intention-to-treat (ITT) principle (KAMPER, 2021).

## Ethics and Dissemination

This project will be submitted for review and approval by the Research Ethics Committee of the Federal University of Rio Grande do Norte (UFRN). All participants will be required to sign the Informed Consent Form prior to enrollment in the study. The results of the study will be disseminated through publications in peer-reviewed scientific journals, social media, and presentations at scientific conferences and academic events.

# REFERENCES

AGERGAARD, A. S. et al. Clinical Outcomes, Structure, and Function Improve With Both Heavy and Moderate Loads in the Treatment of Patellar Tendinopathy: A Randomized Clinical Trial. **American Journal of Sports Medicine**, v. 49, n. 4, p. 982–993, 1 mar. 2021.

ALFREDSON, H.; PIETILÄ, T.; LORENTZON, R. Heavy-Load Eccentric Calf Muscle Training For the Treatment of Chronic Achilles Tendinosis. **The American Journal of Sports Medicine**, v. 26, n. 3, p. 360–366, 1998.

ALGHAMDI, N. H. et al. The Impact of the Degree of Kinesiophobia on Recovery in Patients with Achilles Tendinopathy. **Physical Therapy**, v. 101, n. 11, 1 nov. 2021.

ANDO, R. et al. Relationship Between Drop Jump Training–Induced Changes in Passive Plantar Flexor Stiffness and Explosive Performance. **Frontiers in Physiology**, v. 12, p. 777268, 18 nov. 2021.

ARAMPATZIS, A.; KARAMANIDIS, K.; ALBRACHT, K. Adaptational responses of the human Achilles tendon by modulation of the applied cyclic strain magnitude. **Journal of Experimental Biology**, v. 210, n. 15, p. 2743–2753, ago. 2007.

ARAMPATZIS, A.; MERSMANN, F.; BOHM, S. Individualized Muscle-Tendon Assessment and Training. **Frontiers in Physiology**, v. 11, 26 jun. 2020.

BAXTER, J. R. et al. Exercise Progression to Incrementally Load the Achilles Tendon. **Medicine and Science in Sports and Exercise**, v. 53, n. 1, p. 124–130, 1 jan. 2021.

BEYER, R. et al. Heavy slow resistance versus eccentric training as treatment for achilles tendinopathy: A randomized controlled trial. **American Journal of Sports Medicine**, v. 43, n. 7, p. 1704–1711, 3 jul. 2015.

BISHOP, B. N. et al. Electromyographic Analysis of Gluteus Maximus, Gluteus Medius, and Tensor Fascia Latae During Therapeutic Exercises With and Without Elastic Resistance. **International Journal of Sports Physical Therapy**, v. 13, n. 4,

p. 668–675, ago. 2018.

BOHM, S. et al. Human Achilles tendon plasticity in response to cyclic strain: Effect of rate and duration. **Journal of Experimental Biology**, v. 217, n. 22, p. 4010–4017, 1 nov. 2014.

BREDA, S. J. et al. Effectiveness of progressive tendon-loading exercise therapy in patients with patellar tendinopathy: a randomised clinical trial. **British Journal of Sports Medicine**, v. 55, n. 9, p. 501–509, 1 maio 2021.

BRZYCKI, M. Strength Testing-Predicting a One-Rep Max from Reps-to-Fatigue.

**JOPERD**, v. 64, p. 88–90, jan. 1993.

# CAMALIER, A. A. AVALIAÇÃO DA QUALIDADE DE VIDA RELACIONADA À SAÚDE EM PACIENTES COM DPOC: ESTUDO DE BASE POPULACIONAL COM

**O SF-12**. São Paulo: Unifesp, 2004.

CHAN, A. W. et al. SPIRIT 2013 Statement: Defining Standard Protocol Items for Clinical Trials. **Annals of internal medicine**, v. 158, n. 3, p. 200, 5 fev. 2013.

CHEN, W. et al. Epidemiology of insertional and midportion Achilles tendinopathy in runners: A prospective cohort study. **Journal of Sport and Health Science**, 23 mar. 2023.

CHIMENTI, R. L. et al. Kinesiophobia Severity Categories and Clinically Meaningful Symptom Change in Persons With Achilles Tendinopathy in a Cross-Sectional Study: Implications for Assessment and Willingness to Exercise. **Frontiers in Pain Research**, v. 2, p. 739051, 1 set. 2021.

CHIMENTI, R. L. et al. The effects of pain science education plus exercise on pain and function in chronic Achilles tendinopathy: a blinded, placebo-controlled, explanatory, randomized trial. **Pain**, v. 164, p. 47–65, 17 jun. 2023.

COLLINGS, T. J. et al. Gluteal Muscle Forces during Hip-Focused Injury Prevention and Rehabilitation Exercises. **Medicine & Science in Sports & Exercise**, v. 55, n. 4,

p. 650–660, abr. 2023.

DE JONGE, S. et al. Incidence of midportion Achilles tendinopathy in the general population. **British Journal of Sports Medicine**, v. 45, n. 13, p. 1026–1028, 1 out. 2011.

BALDON, R. M. et al. Effects of functional stabilization training on pain, function, and lower extremity biomechanics in women with patellofemoral pain: A randomized clinical trial. **Journal of Orthopaedic and Sports Physical Therapy**, v. 44, n. 4, p. 240–251, 2014.

DE MESQUITA, G. N. et al. Cross-cultural adaptation and measurement properties of the brazilian Portuguese version of the victorian institute of sport assessment-achilles (VISA-A) questionnaire. **Journal of Orthopaedic and Sports Physical Therapy**, v. 48, n. 7, p. 567–573, 1 jul. 2018.

DE VOS, R. J. et al. Dutch multidisciplinary guideline on Achilles tendinopathy.

**British Journal of Sports Medicine**, v. 55, n. 20, p. 1125–1134, 1 out. 2021.

DIAS ARAÚJO, E. H. et al. Intervention Treating Kinetic Chain Factors versus Heavy- Slow Resistance Training in Athletes with Patellar Tendinopathy: Protocol for a Randomized Blind Clinical Trial. **Muscle Ligaments and Tendons Journal**, v. 12, n. 03, p. 386, jul. 2022.

DISTEFANO, L. J. et al. Gluteal muscle activation during common therapeutic exercises. **Journal of Orthopaedic and Sports Physical Therapy**, v. 39, n. 7, p. 532–540, 2009.

ESCRICHE-ESCUDER, A.; CASANÃ, J.; CUESTA-VARGAS, A. I. Original research:

Load progression criteria in exercise programmes in lower limb tendinopathy: a systematic review. **BMJ Open**, v. 10, n. 11, 19 nov. 2020.

FERNANDEZ, M. R. et al. Concurrent validity and reliability of a mobile iOS application used to assess calf raise test kinematics. **Musculoskeletal Science and Practice**, v. 63, p. 102711, 1 fev. 2023.

HABETS, B. et al. No Difference in Clinical Effects When Comparing Alfredson Eccentric and Silbernagel Combined Concentric-Eccentric Loading in Achilles Tendinopathy: A Randomized Controlled Trial. **Orthopaedic Journal of Sports Medicine**, v. 9, n. 10, 2021.

HANLON, S. L.; POHLIG, R. T.; SILBERNAGEL, K. G. Beyond the diagnosis: Using patient characteristics and domains of tendon health to identify latent subgroups of achilles tendinopathy. **Journal of Orthopaedic and Sports Physical Therapy**, v. 51, n. 9, p. 440–450, 1 set. 2021.

HASANI, F. et al. Are Plantarflexor Muscle Impairments Present Among Individuals with Achilles Tendinopathy and Do They Change with Exercise? A Systematic Review with Meta-analysis. **Sports Medicine - Open**, v. 7, n. 1, 1 dez. 2021.

JAESCHKE, R.; SINGER, J.; GUYATT, G. H. Measurement of Health Status Ascertaining the Minimal Clinically Important Difference. **Controlled Clinical Trials**,

v. 10, p. 407–415, 1989.

JAKOBSEN, M. D. et al. Muscle activity during knee-extension strengthening exercise performed with elastic tubing and isotonic resistance. **International Journal of Sports Physical Therapy**, v. 7, n. 6, p. 606, dez. 2012.

JOHANNSEN, F.; JENSEN, S.; WETKE, E. 10-year follow-up after standardised treatment for Achilles tendinopathy. **BMJ Open Sport — Exercise Medicine**, v. 4, n. 1, p. 415, 1 out. 2018.

KAMPER, S. J. Per-protocol, intention-to-treat, and complier average causal effects analyses in randomized controlled trials: Linking evidence to practice. **Journal of Orthopaedic and Sports Physical Therapy**, v. 51, n. 6, p. 314–315, 1 jun. 2021.

KONGSGAARD, M. et al. Corticosteroid injections, eccentric decline squat training and heavy slow resistance training in patellar tendinopathy. **Scandinavian journal of Medicine & Science in Sports**, v. 19, p. 790–802, 2009.

KUJALA, U. M.; SARNA, S.; KAPRIO, J. Cumulative Incidence of Achilles Tendon Rupture and Tendinopathy in Male Former Elite Athletes. **Clin J Sport Med**, v. 15, n. 3, p. 133–135, 2005.

LAGAS, I. F. et al. Victorian institute of sport assessment-achilles (visa-a) questionnaire—minimal clinically important difference for active people with midportion achilles tendinopathy: A prospective cohort study. **Journal of Orthopaedic and Sports Physical Therapy**, v. 51, n. 10, p. 510–516, 1 out. 2021.

LAGAS, I. F. et al. One fifth of patients with Achilles tendinopathy have symptoms after 10 years: A prospective cohort study. **Journal of Sports Sciences**, v. 40, p. 2475–2483, 23 dez. 2022.

LAZARCZUK, S. L. et al. Mechanical, Material and Morphological Adaptations of Healthy Lower Limb Tendons to Mechanical Loading: A Systematic Review and Meta-Analysis. **Sports Medicine**, v. 52, n. 10, p. 2405–2429, 1 out. 2022.

LEWIS, T.; COOK, J. Fluoroquinolones and tendinopathy: A guide for athletes and sports clinicians and a systematic review of the literature. **Journal of Athletic Training**, v. 49, n. 3, p. 422–427, 2014.

LIEBENSON, C. Training for speed. **Journal of Bodywork and Movement Therapies**, v. 13, n. 4, p. 362–363, out. 2009.

MALLIARAS, P. Physiotherapy management of Achilles tendinopathy. **Journal of Physiotherapy**, v. 68, n. 4, p. 221–237, 1 out. 2022.

MARTIN, R. L. et al. Achilles pain, stiffness, and muscle power deficits: Midportion achilles tendinopathy revision 2018. **Journal of Orthopaedic and Sports Physical Therapy**, v. 48, n. 5, p. A1–A38, 1 maio 2018.

MATSUDO, S. et al. Questionario internacional de ativi dade fisica (i paq): estudo de vall dade e reprodutibilidade no brasil international physical activity questionnaire

(lpaq): study of validity and reliability in brazil. **Atividade física e saúde**, v. 6, n. 2, p. 05–18, 2001.

MCAULIFFE, S. et al. Altered Strength Profile in Achilles Tendinopathy: A Systematic Review and Meta-Analysis. **Journal of Athletic Training**, v. 54, n. 8, p. 889–900, 1 ago. 2019.

MCCORMACK, J. et al. The minimum clinically important difference on the visa‐a and lefs for patients with insertional achilles tendinopathy. **International Journal of Sports Physical Therapy**, v. 10, n. 5, p. 639, out. 2015.

O’NEILL, S. et al. Acute sensory and motor response to 45-s heavy isometric holds for the plantar flexors in patients with Achilles tendinopathy. **Knee Surgery, Sports Traumatology, Arthroscopy**, v. 27, n. 9, p. 2765–2773, 1 set. 2019.

PINTO GUEDES, D.; CORREA LOPES, C.; ELISABETE RIBEIRO PINTO GUEDES,

J. Reprodutibilidade e validade do Questionário Internacional de Atividade Física em adolescentes ARTIGO ORIGINAL. **Rev Bras Med Esporte**, v. 11, n. 2, p. 151–158, 2005.

PRICE, D. D. et al. The Validation of Visual Analogue Scales as Ratio Scale Measures for Chronic and Experimental Pain. **Pain**, v. 17, p. 45–56, 1983.

RABUSIN, C. L. et al. Efficacy of heel lifts versus calf muscle eccentric exercise for mid-portion Achilles tendinopathy (HEALTHY): a randomised trial. **British Journal of Sports Medicine**, v. 55, n. 9, p. 486–492, 1 maio 2021.

RATAMESS, N. A. et al. Progression models in resistance training for healthy adults. **Medicine and Science in Sports and Exercise**, v. 41, n. 3, p. 687–708, mar. 2009. REYNOLDS, J. M.; GORDON, T. J.; ROBERGS, R. A. **PREDICTION OF ONE REPETITION MAXIMUM STRENGTH FROM MULTIPLE REPETITION MAXIMUM**

## TESTING AND ANTHROPOMETRYJournal of Strength and Conditioning Research. [s.l: s.n.].

ROBINSON, J. M. et al. The VISA-A questionnaire: a valid and reliable index of the clinical severity of Achilles tendinopathy. **British Journal of Sports Medicine**, v. 35,

n. 5, p. 335, 2001.

SCATTONE SILVA, R. et al. Lower limb strength and flexibility in athletes with and without patellar tendinopathy. **Physical Theraoy in Sport**, v. 20, p. 19–25, 2016.

SCHULZ, K. F.; ALTMAN, D. G.; MOHER, D. CONSORT 2010 Statement: updated

guidelines for reporting parallel group randomised trials. **BMC Medicine**, v. 8, p. 18, 24 mar. 2010.

SILBERNAGEL, K.; BRORSSON, A.; LUNDBERG, M. The majority of patients with Achilles tendinopathy recover fully when treated with exercise alone: A 5-year follow- up. **American Journal of Sports Medicine**, v. 39, n. 3, p. 607–613, 17 mar. 2011.

SILBERNAGEL, K. G. et al. Eccentric overload training for patients with chronic Achilles tendon pain-a randomised controlled study with reliability testing of the evaluation methods. **Scand J Med Sci Sports**, v. 11, p. 197–206, 2001.

SILBERNAGEL, K. G. et al. Evaluation of lower leg function in patients with Achilles tendinopathy. **Knee Surgery, Sports Traumatology, Arthroscopy**, v. 14, n. 11, p. 1207–1217, 21 nov. 2006.

SILBERNAGEL, K. G. et al. Continued sports activity, using a pain-monitoring model, during rehabilitation in patients with Achilles tendinopathy: a randomized controlled study. **The American Journal of Sports Medicine**, v. 35, n. 6, p. 897–907, 1 jun. 2007.

SILBERNAGEL, K. G.; CROSSLEY, K. M. A proposed return-to-sport program for patients with midportion achilles tendinopathy: Rationale and implementation. **Journal of Orthopaedic and Sports Physical Therapy**, v. 45, n. 11, p. 876–886, 1 nov. 2015.

SIQUEIRA, F. B.; TEIXEIRA-SAMELA, L. F.; MAGALÃES, L. DE C. Análise das

propriedades psicométricas da versão brasiliera da escala TAMPA de cinesiofobia Siqueira et al, 2007. **Acta Ortop Bras**, v. 15, n. 1, p. 19–24, 2007.

SLEESWIJK VISSER, T. S. O. et al. Impact of chronic Achilles tendinopathy on health-related quality of life, work performance, healthcare utilisation and costs. **BMJ Open Sport and Exercise Medicine**, v. 7, n. 1, 26 mar. 2021.

STENSRUD, S.; ROOS, E. M.; RISBERG, M. A. A 12-week exercise therapy program in middle-aged patients with degenerative meniscus tears: A case series with 1-year follow-up. **Journal of Orthopaedic and Sports Physical Therapy**, v. 42,

n. 11, p. 919–931, 2012.

STEVENS, M.; TAN, C. W. Effectiveness of the alfredson protocol compared with a lower repetition-volume protocol for midportion achilles tendinopathy: A randomized controlled trial. **Journal of Orthopaedic and Sports Physical Therapy**, v. 44, n. 2,

p. 59–67, 1 fev. 2014.

SUZUKI, T.; CHINO, K.; FUKASHIRO, S. Gastrocnemius and soleus are selectively activated when adding knee extensor activity to plantar flexion. **Human Movement Science**, v. 36, p. 35–45, 2014.

VAN DER PLAS, A. et al. A 5-year follow-up study of Alfredson’s heel-drop exercise programme in chronic midportion Achilles tendinopathy. **British Journal of Sports Medicine**, v. 46, n. 3, p. 214, 3 mar. 2012.

VAN DER VLIST, A. C. et al. Which treatment is most effective for patients with Achilles tendinopathy? A living systematic review with network meta-analysis of 29 randomised controlled trials. **British Journal of Sports Medicine**, v. 55, n. 5, p. 249, 1 mar. 2021.

VLAEYEN, J. W. S. et al. **Fear of movement/( re) injury in chronic low back pain and its relation to behavioral performancePain**. [s.l: s.n.].

WARE, J. E.; KOSINSKI, M.; KELLER, S. D. **A 12-Item Short-Form Health Survey: Construction of Scales and Preliminary Tests of Reliability and Validity**. Disponível em: <https://oce-ovid.ez18.periodicos.capes.gov.br/article/00005650- 199603000-00003/HTML>. Acesso em: 3 jul. 2022.

**Supplementary Materials**

**Supplementary Material**1: *International Physical Activity Questionnaire (IPAQ-SF)* / Translated and validated version in Brazilian Portuguese.
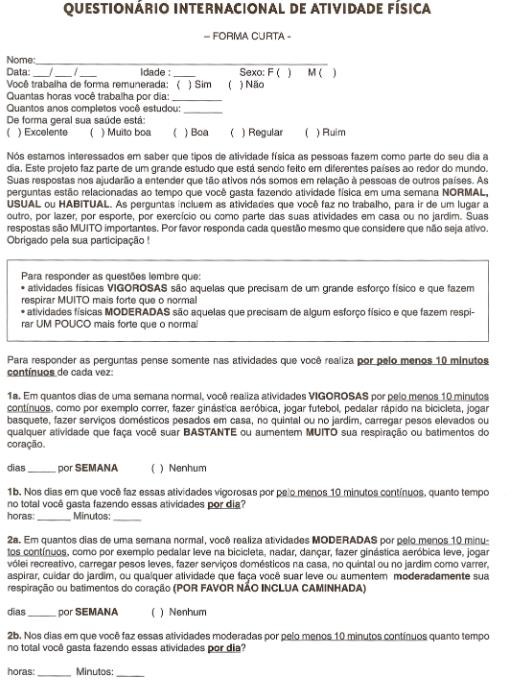


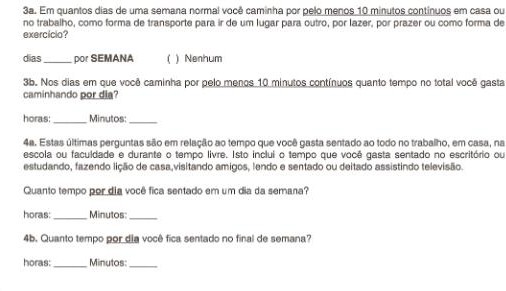


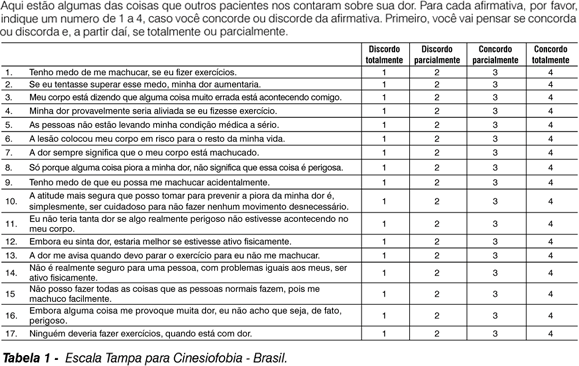
**Supplementary Material**2: *Tampa Scale of Kinesiophobia (TSK)* / Translated and validated version in Brazilian Portuguese.

**Supplementary Material**3: *Victorian Institute of Sport Assessment-Achilles* (VISA-A) / Translated and validated version in Brazilian Portuguese
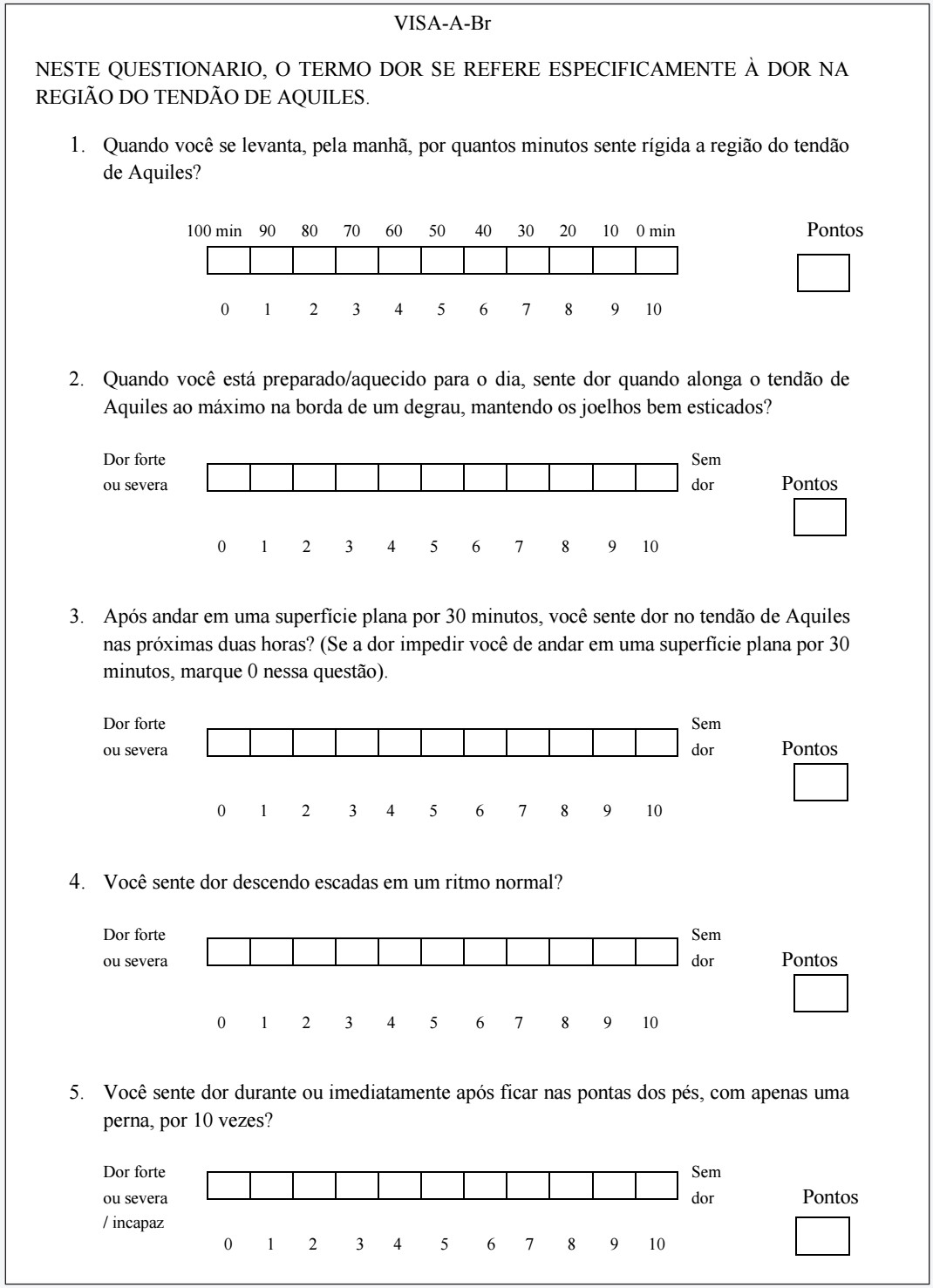


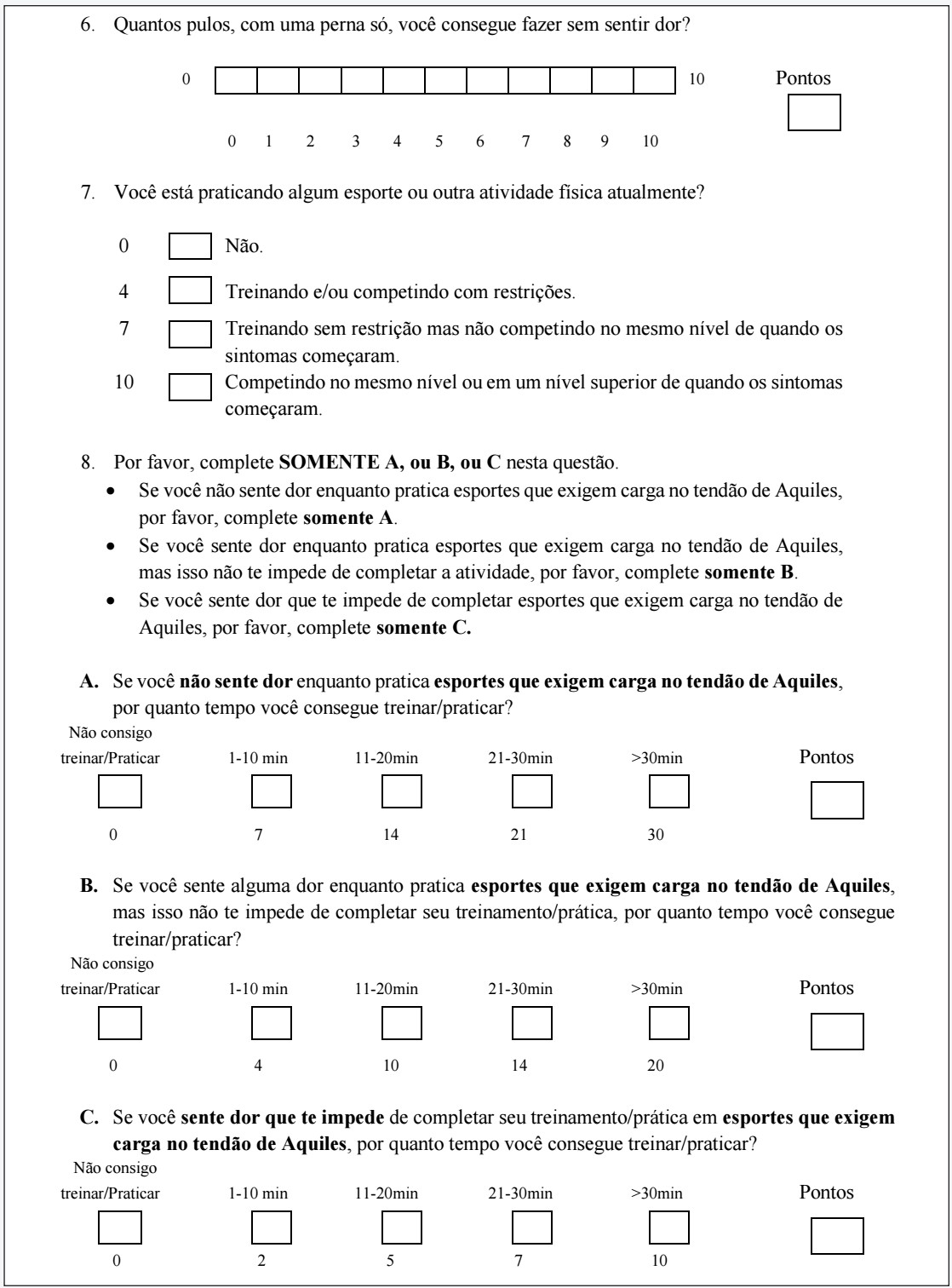


**Supplementary Material**4: *Short-Form Health Survey (SF - 12) -* Translated and validated version in Brazilian Portuguese


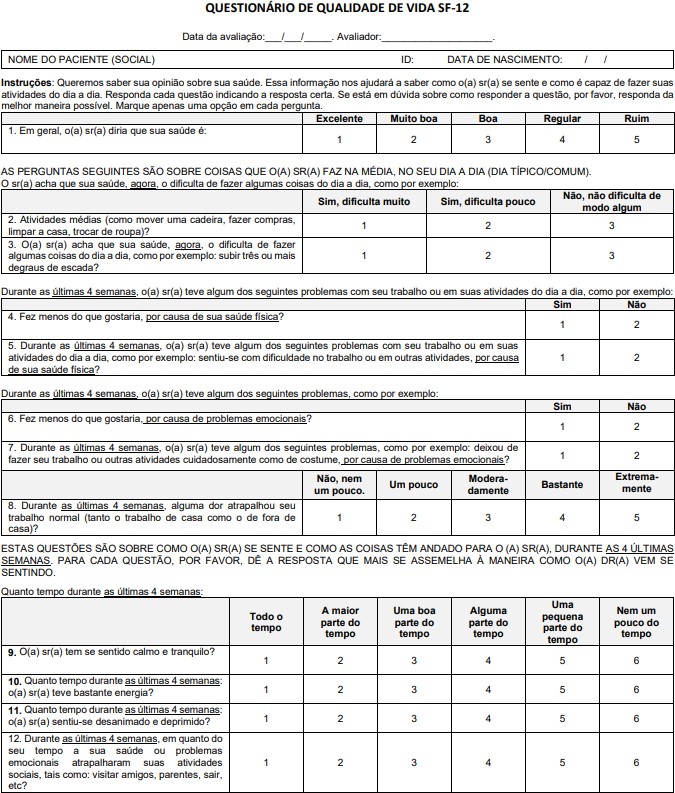


**Supplementary Material**5: *Global Rating of Change* (GROC).

Name: Date: / /

Regarding your symptoms, you feel:

+7: Extremely Better ( )

+6 : ( )

+5: Much better ( )

+4 :( )

+3: Better ( )

+2 : ( )

+1: Slightly better ( )

0: No change ( )

1. -1: A little worse ( )

-2: ( )

1. -3: Worst ( )

-4:( )

1. -5: Much worse ( )

-6:( )

1. -7: Extremely Worse ( )
