# Supplementary material for: Comparison between moderate-load and high-load exercises in the rehabilitation of runners with Achilles tendinopathy: Protocol for a blind randomized controlled trial
Source: PLoS One. 2026 Mar 2;21(3):e0342934. doi: 10.1371/journal.pone.0342934 (PMC12952620; doi:10.1371/journal.pone.0342934)
Supplement: S3 File — (DOCX) [file pone.0342934.s003.docx]

| ASSESSMENT OF ISOMETRIC STRENGTH | | | |
| --- | --- | --- | --- |
| Test | Positioning | Command | Illustration |
| HIP EXTENSION STRENGTH | Hip extension strength will be assessed with the participant in a prone position with the hip in a neutral position in all 3 planes and the knee at 90° flexion. An inelastic strap attached to the stretcher will go around the participant's pelvis for stabilization. The dynamometer will be positioned immediately proximal to the popliteal fossa of the evaluated limb and will be fixed by a second inelastic strap attached to the stretcher. | The participant will be instructed to “push trying to move the foot towards the ceiling” | 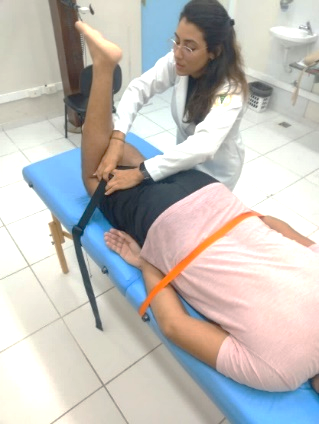 |
| ANKLE PLANTAR FLEXION STRENGTH | Ankle plantar flexion strength will be assessed with the participant in a prone position with the joints of the evaluated lower limb in a neutral position. The handheld dynamometer will be positioned on the plantar surface of the metatarsal head, fixed by an inelastic strap that will pass around the stretcher. | The participant will be instructed to “use maximum force to bring the toes down” | 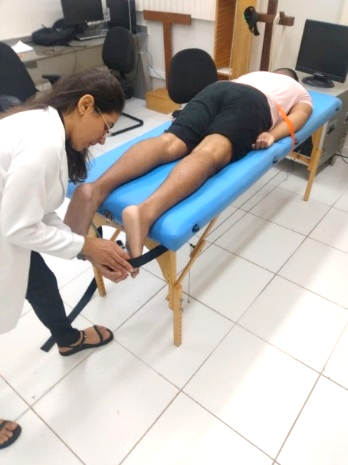 |
| KNEE EXTENSION STRENGTH | Knee extension strength will be assessed with the participant in a supine position with the knee flexed at 30°. The participant will be instructed to cross the upper limbs over the trunk. A non-deformable roller will be used below the knee at the level of the popliteal fossa to maintain this angulation. An inelastic strap passing around the stretcher will be used for stabilizing the handheld dynamometer, which will be positioned in front of the ankle, at the midpoint between the malleoli. | The participant will be instructed to “push trying to extend the knee” | 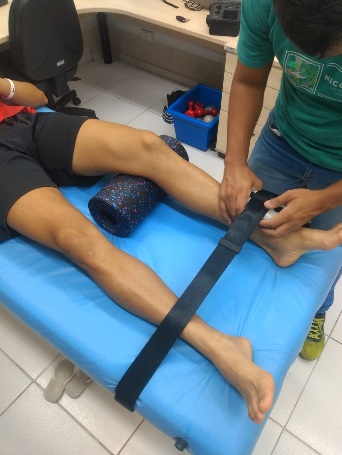 |

In all maximum isometric strength assessments, four repetitions will be performed. The first repetition will serve to familiarize the participant with the test, and the following three repetitions will be used for data analysis. In each repetition, the participant will be instructed to maintain maximum strength for 5 seconds, and between each repetition, there will be a 15-second rest. The action lengths for hip extension, knee extension, and ankle plantar flexion torques were defined, respectively, as the distance from the greater trochanter to the lateral femoral epicondyle; the lateral femoral epicondyle to the lateral malleolus; and the posterior aspect of the calcaneus to the first metatarsophalangeal joint.

| ASSESSMENT OF THE FUNCTION OF THE ANKLE PLANTAR FLEXORS | | | |
| --- | --- | --- | --- |
| Test | Description | Instructions | Illustration |
| SINGLE-LEG HEEL RISE TEST | The single-leg heel rise test evaluates the number of heel raises that the participant can perform in single-leg support. For this test, the participant will be positioned in single-leg support on a ramp with a 10° inclination with a 24mm black marker positioned immediately below the lateral malleolus. They will then be instructed to use the support of two fingers on the wall, at shoulder height, to maintain balance. A metronome will be used to maintain a frequency of 30 toe raises per minute (60Hz, one second to go up and one second to go down). The test will be recorded using a smartphone at a frequency of 60 Hz, which will be positioned on a device placed 30cm laterally to the decline board. To measure the total work of the activity, we will use the Calf Raise application. | Standing on one leg, the participant will be instructed to rise as high as possible on each heel raise and then lower the heel to the starting position while keeping the knee extended and the trunk straight. The test will be terminated when the participant is unable to continue, is unable to maintain the frequency, or is unable to perform a complete heel raise. | 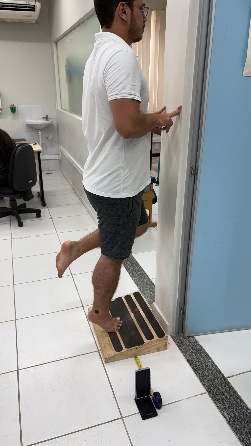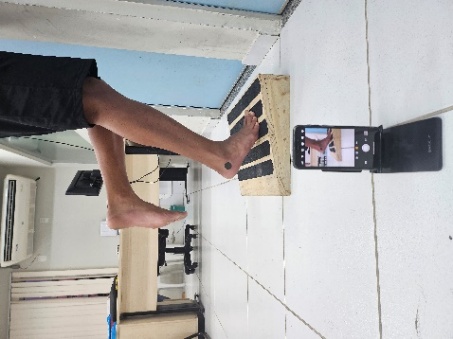 |
| SINGLE-LEG DROP VERTICAL JUMP TEST | The single-leg drop vertical jump test will begin with measuring the height from the ground to the fingertips of the participant's hands with the upper limb elevated, raising the hand as high as possible. After that, they will be positioned on a box with 20 centimeters of height, which will be positioned next to the wall. The participants will be given a piece of sticky paper (post-it) to hold with their fingers and, when performing the jump, place the paper at the highest point they can reach. | The participants will be instructed to let themselves fall from the box, land only with the evaluated limb, and jump as high as possible, placing the post-it on the wall. | 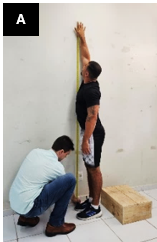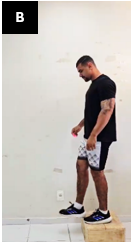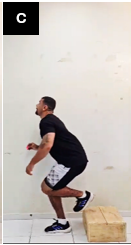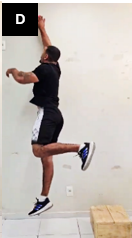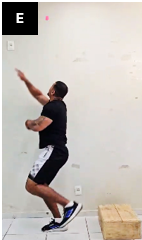 |
