# Supplementary material for: Comparison between moderate-load and high-load exercises in the rehabilitation of runners with Achilles tendinopathy: Protocol for a blind randomized controlled trial
Source: PLoS One. 2026 Mar 2;21(3):e0342934. doi: 10.1371/journal.pone.0342934 (PMC12952620; doi:10.1371/journal.pone.0342934)
Supplement: S4 File — (DOCX) [file pone.0342934.s004.docx]

**TREATMENT PROTOCOL**

- **Frequency of treatment sessions**: 3 times a week (one of them supervised).
- Both groups will perform the identical exercises.
- **Warm-up**: Before all exercises, participants will perform a series of the same exercise, without external load.
- **Rest**: 1 minute between each series for all exercises.
- **Load adjustments**: Every two weeks, adjustments to the load will be made.
- **All exercises** must be performed with 3 seconds for the concentric phase and 3 seconds for the eccentric phase. 1 repetition maximum (RM) testing will be conducted using the same tempo.
- Series and repetitions: For specific triceps surae exercises, we will follow **Table 1**. For the exercises of the kinetic chain muscles (quadriceps and gluteus maximus), the exercises will follow the guidelines of the American College of Sports Medicine for strength training:

**Table 1:** Triceps surae exercise protocols for both groups.

|  | W  1-2 | W  3-4 | W  5 | W  6 | W  7 | W  8 | W  9-12 | Total number of repetitions |
| --- | --- | --- | --- | --- | --- | --- | --- | --- |
| HLG |  |  |  |  |  |  |  |  |
| % of 1RM | 55 | 65 | 75 | 75 | 85 | 85 | 90 | 1.054 |
| Repetitions | 15 | 15 | 10 | 8 | 8 | 6 | 4 |  |
| Sets | 3 | 3 | 3 | 4 | 4 | 4 | 5 |  |
| MLG |  |  |  |  |  |  |  |  |
| % of 1RM | 55 | 55 | 55 | 55 | 55 | 55 | 55 | 1.053 |
| Repetitions | 17 | 15 | 12 | 10 | 8 | 7 | 6 |  |
| Sets | 3 | 3 | 3 | 3 | 3 | 3 | 3 |  |

**a) First phase (week 1 to 4)** - 3 sets of 12 repetitions at 60% of 1RM.

**b) Second phase (week 5 to 8)** - 3 sets of 10 repetitions at 70% of 1RM.

**c) Third phase (week 9 to 12)** - 3 sets of 8 repetitions at 80% of 1RM.

**1. PHASE 1 EXERCISES (1st to 4th WEEK)**

**Exercise 1 -** Seated heel raise (2-leg):


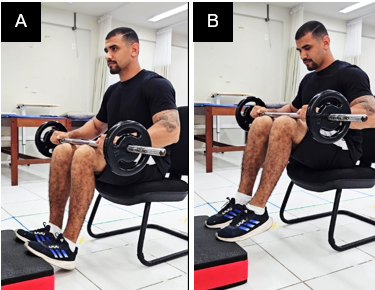


**High Load Group (HLG)**

- Sets: 3 x 15:
- 1st and 2nd week - 55% of 1RM.
  - 3rd and 4th week - 65% of 1RM.

**Moderate Load Group (MLG)**

- Sets: 3 x 17:
  - 1st and 2nd week - 55% of 1RM.
- Sets: 3 x 15:
  - 3rd and 4th week - 55% of 1RM.
- **Description**: Sitting on a chair, without resting your back, place the tips of both feet on a step and raise your heels as high as possible, contracting your calf muscles and then slowly lowering the heel with maximal range of motion.
- **Demonstration Video**: <https://encurtador.com.br/6e4VA>

**Exercise 2 -** Standing heel raise (2-leg):


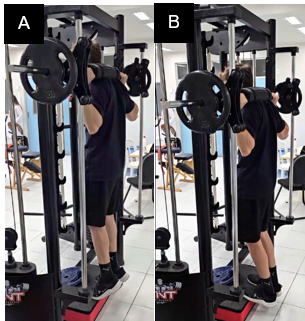


**HLG**

- Sets: 3 x 15:
- 1st and 2nd week - 55% of 1RM.
  - 3rd and 4th week - 65% of 1RM.

**MLG**

- Sets: 3 x 17:
  - 1st and 2nd week - 55% of 1RM.
- Sets: 3 x 15:
  - 3rd and 4th week - 55% of 1RM.
- **Description**: Position the bar of the Smith machine on your shoulders. Stand with your toes on a step, with the feet shoulder-width apart. Raise your heels as high as possible, and then slowly lower the heels with maximal range of motion.
- **Demonstration Video:** <https://encurtador.com.br/wJETC>

**Exercise 3 -** Knee extension machine (2-leg):


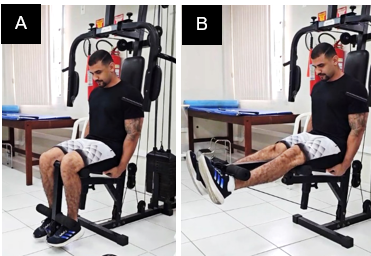


**HLG and MLG**

- Sets: 3 x 12 – 60% of 1RM.
- **Description**: Sitting on the leg extension machine, adjust the seat so that your knees are aligned with the machine's axis and your knee is at approximately 90º of flexion. Fully extend your knees, maintaining control of the movement, and return to the starting position in a controlled manner.
- **Demonstration Video**: <https://encurtador.com.br/DOSYV>

**Exercise 4 -** Clam with 60° of hip flexion:


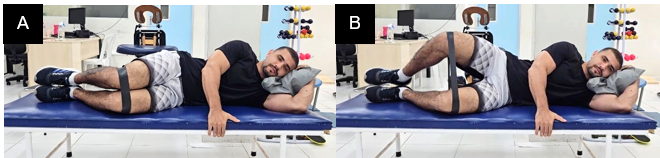


**HLG and MLG**

- Sets: 3 x 12 – 60% of 1RM.
- **Description**: Lie on your side with your knees bent at a 60º angle. Place the elastic band around your knees Raise your upper knee while keeping your feet in contact, as high as possible and then return to the starting position in a controlled manner.
- **Demonstration Video**: <https://encurtador.com.br/ManVQ>

**2. PHASE 2 EXERCISES (5th to 8th WEEK):**

**Exercise 1 -** Seated heel raise (1-leg):


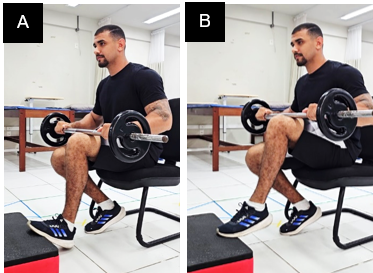


**HLG**

- 5th week - 3x10 with 75% of 1RM.
- 6th week - 4x8 with 75% of 1RM.
- 7th week - 4x8 with 85% of 1RM.
- 8th week - 4x6 with 85% of 1RM.

**MLG**

- 5th week - 3x12 with 55% of 1RM.
- 6th week - 3x10 with 55% of 1RM.
- 7th week - 3x8 with 55% of 1 RM.
- 8th week - 3x7 with 55% of 1RM.
- **Description:** Sitting on a chair, without resting your back, place the tip of one of your foot on a step and raise your heel as high as possible, contracting your calf muscles and then slowly lowering the heel with maximal range of motion.
- **Demonstration Video**: <https://encurtador.com.br/Atf6F>

**Exercise 2 -** Standing heel raise (1-leg):


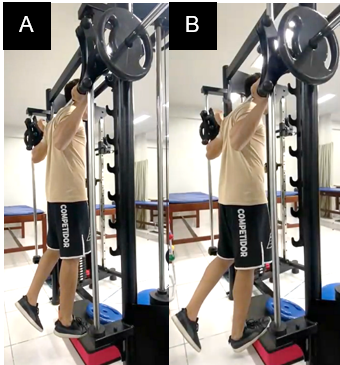


**HLG**

- 5th week - 3x10 with 75% of 1RM.
- 6th week - 4x8 with 75% of 1RM.
- 7th week - 4x8 with 85% of 1RM.
- 8th week - 4x6 with 85% of 1RM.

**MLG**

- 5th week - 3x12 with 55% of 1RM.
- 6th week - 3x10 with 55% of 1RM.
- 7th week - 3x8 with 55% of 1 RM.
- 8th week - 3x7 with 55% of 1RM.
- **Description**: Position the bar of the Smith machine on your shoulders. Stand with the tip of one foot on a step, raise your heel as high as possible, and then slowly lower the heel with maximal range of motion.
- **Demonstration Video**: <https://shre.ink/8kwF>

**Exercise 3 -** Knee extension in machine (1-leg):


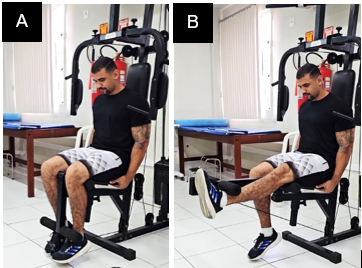


**HLG and MLG**

- Sets: 3 x 10 – 70% of 1RM.
- **Description**: Sitting on the leg extension machine, adjust the seat so that your knee is aligned with the machine's axis at approximately 90º of knee flexion. Fully extend your knee, maintaining control of the movement, and then return to the starting position in a controlled manner.
- **Demonstration Video**: <https://shre.ink/8kwX>

**Exercise 4 -** Single limb deadlift:

**
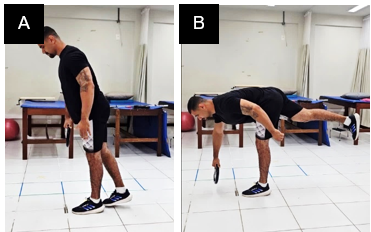
**

**HLG and MLG**

- Sets: 3 x 10 – 70% of 1RM.
- **Description**: Standing in one leg, hold the weight in opposite hand. Lean your torso forward, lifting the opposite leg back. Keep your spine straight and go down until your trunk is parallel to the ground and go back to the initial position.
- **Demonstration Video**: <https://shre.ink/8kwq>

**3. PHASE 3 EXERCISES (9^th^ to 12th WEEK):**

During Phase 3, in unsupervised sessions, all exercises from phase two will continue with adjustments to sets, number of repetitions and loads. In the supervised session we will perform a series of plyometric exercises.

**3.1 Unsupervised session**

**Exercise 1 -** Seated heel raise (1-leg):


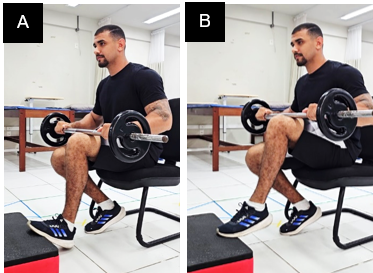


**HLG**

- 9th to 12th week - 4x5 with 90% of 1RM.

**MLG**

- 9th to 12th week - 3x6 with 55% of 1RM.
- **Description**: Sitting on a chair, without resting your back, place the tip of one of your foot on a step and raise your heel as high as possible, contracting your calf muscles and then slowly lowering the heel with maximal range of motion.
- **Demonstration Video**: <https://encurtador.com.br/Atf6F>

**Exercise 2 -** Standing heel raise (1-leg):


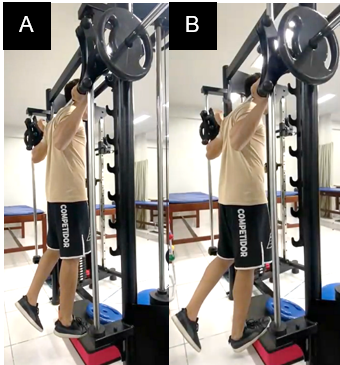


**HLG**

- 9th to 12th week - 4x5 with 90% of 1RM.

**MLG**

- 9th to 12th week - 3x6 with 55% of 1RM.
- **Description**: Position the bar of the Smith machine on your shoulders. Stand with the tip of one foot on a step, raise your heel as high as possible, and then slowly lower the heel with maximal range of motion.
- **Demonstration Video**: <https://shre.ink/8kwF>

**Exercise 3 -** Knee extension in machine (1-leg):


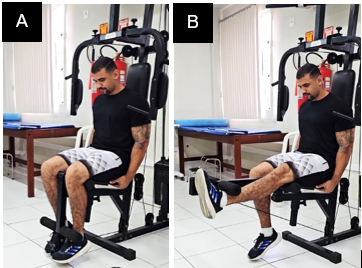


**HLG and MLG**

- Sets: 3 x 8 – 80% of 1RM.
- **Description**: Sitting on the leg extension machine, adjust the seat so that your knee is aligned with the machine's axis at approximately 90º of knee flexion. Fully extend your knee, maintaining control of the movement, and then return to the starting position in a controlled manner.
- **Demonstration Video**: <https://shre.ink/8kwX>

**Exercise 4 -** Single-limb deadlift:

**
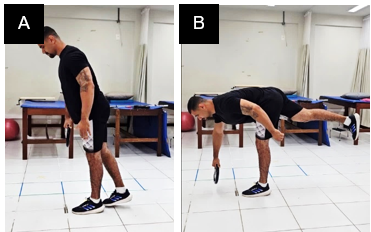
**

**HLG and MLG**

- Sets: 3 x 8 – 80% of 1RM.
- **Description**: Standing in one leg, hold the weight in opposite hand. Lean your torso forward, lifting the opposite leg back. Keep your spine straight and go down until your trunk is parallel to the ground and go back to the initial position.
- **Demonstration Video**: <https://shre.ink/8kwq>

**3.2. Phase 3: Supervised Session**

**Exercise 1 -** Fast two leg hops:


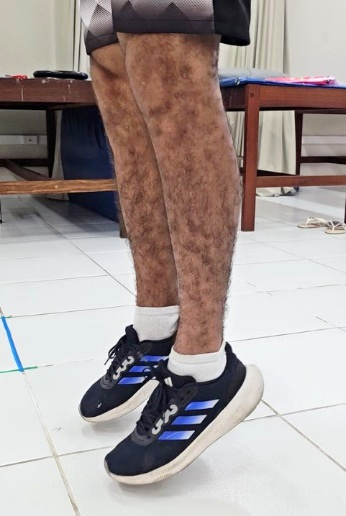


**HLG and MLG**

- Sets: 3x 20 seconds.
- 5 seconds will be added to each session.
- **Description**: Stand with your feet shoulder-width apart. Bend your knees slightly, then jump as high and quickly as possible. When landing, keep your knees slightly bent to absorb the impact. Repeat quickly.
- **Demonstration Video**: <https://encurtador.com.br/SsHp9>

**Exercise 2 -** Fast alternating one leg jumps:


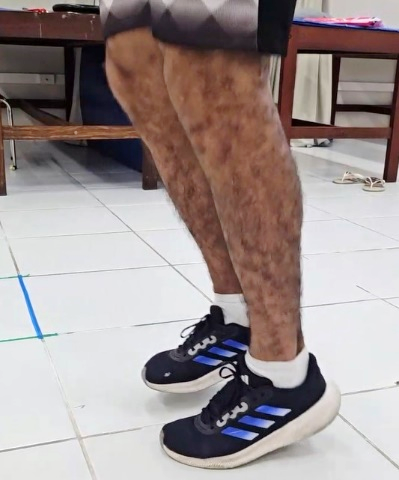


**HLG and MLG**

- Sets: 3x 20 seconds.
- 5 seconds will be added to each session.
- **Description**: Stand with your feet shoulder-width apart. Perform alternating jumps, changing the supporting leg with each jump. Keep the movement quick and controlled. Bend your knees when landing.
- **Demonstration Video**: <https://encurtador.com.br/c2EmX>

**Exercise 3 -** Double-leg drop vertical jump:


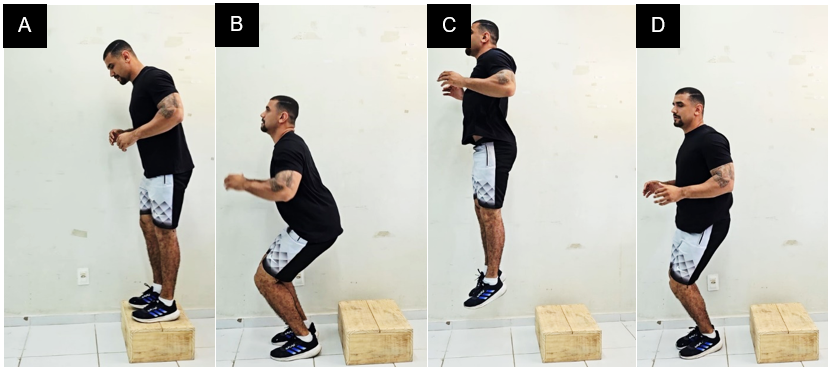


**HLG and MLG**

- Sets: 5x20 jumps.
- 9th and 10th week.
- **Description**: Climb onto a 20cm box. Let yourself fall, bending your knees and hips as your feet touch the floor. Then, jump as high as possible. When landing, absorb the impact by bending your knees.
- **Demonstration Video**: <https://abrir.link/XYatO>

**Exercise 4 -** Single-leg drop vertical jump:


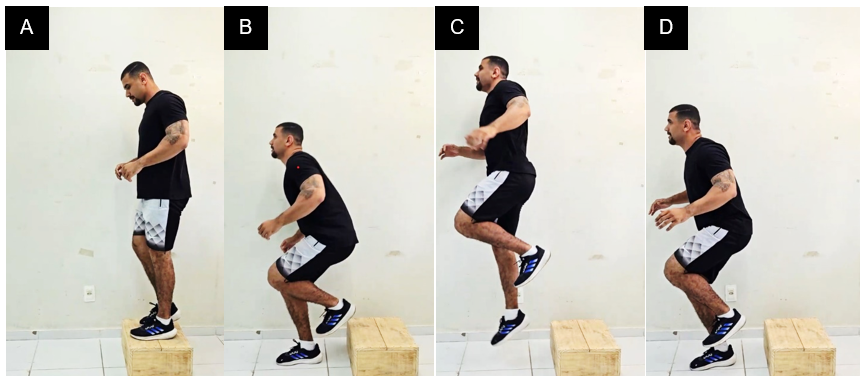


**HLG and MLG**

- Sets: 5x20 jumps.
- 9th and 10th week.
- **Description**: Climb onto a 20cm box. Let yourself fall, bending your knee and hip as you touch one foot to the floor. Then jump as high as possible with your supporting leg. When landing, absorb the impact by bending your knee. Repeat alternating your legs.
- **Demonstration Video**: <https://abrir.link/xsacM>

**Exercise 5 -** Wall running drill:

**
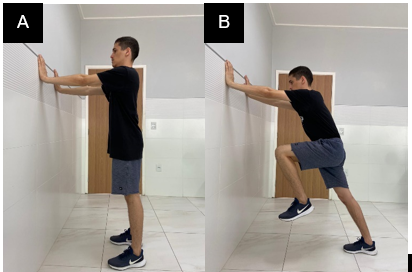
**

**HLG and MLG**

- Sets: 3 x 12 repetitions (alternating between 3 movements).
- **Description**: Start standing with your hands resting on the wall, at shoulder height, then take a step back, supporting only your back leg and both hands resting on the wall. Bend the knee and hip of the front leg. Perform a fast running-like movement, alternating your legs quickly. Keep your trunk stable and your arms firm against the wall to maximize the effectiveness of the exercise.
- **Demonstration Video**: <https://encurtador.com.br/GVE3A>

If the participant is unable to complete the prescribed sets or repetitions of the plyometric exercises due to fatigue or tendon pain, the number of repetitions will be reduced based on the participant’s self-reported pain. Pain during the exercises is acceptable if rated ≤5 on the Visual Analog Scale (VAS). However, pain or discomfort should not worsen after training. If exercise-induced pain persists for more than 3 to 4 hours post-session, the training load will be reduced in the following session.
